# Supplementary material for: Bio-inspired asymmetric Zn-N2O2 single-atom catalysts via natural skeleton for efficient N-alkylation of nitroarenes with alcohols
Source: Nat Commun. 2026 Mar 5;17:2242. doi: 10.1038/s41467-026-70172-1 (PMC12963498; doi:10.1038/s41467-026-70172-1)
Supplement: Supplementary file 1 — Supplementary Information [file 41467_2026_70172_MOESM1_ESM.pdf]

Supplementary Information

Bio-inspired Asymmetric Zn-N_2O_2 Single-atom Catalysts via Natural Skeleton for Efficient N-alkylation of Nitroarenes with Alcohols

Yu Huang[1], Yan Li[2], Xiaogang Yin[1,*], Qiudi Zhu[1], Mei He[1], Xueqin Chang[1], Xuefei Liu[1], Wu Li[2], Aiwen Lei[2,*] and Xianglin Pei[1,*]

[1] School of Chemistry and Materials Science, Guizhou Normal University, Guiyang 550025, China.

[2] College of Chemistry and Molecular Sciences, Wuhan University, Wuhan 430072, China.

* Corresponding Author: A. Lei, E-mail: aiwenlei@whu.edu.cn; X. Pei, E-mail: xianglinpei@163.com; X. Yin, E-mail: m13885115516@163.com.

Table of contents

S1. Experimental Section....................3

1.1 Materials....................3

1.2 Characterization....................3

Supplementary Figures....................5

Supplementary Tables....................26

S2. Computational Details....................31

S3. NMR Data of the Products....................32

S3.1 Scope of Nitroarenes....................32

S3.2 Scope of Alcohols....................35

S3.3 Scope of Nitroarenes and Alcohols....................38

S3.4 Substrates of Heterocycles....................41

S3.5 Scope of Drugs....................45

S4. NMR Image of the Products....................48

S5. Supplementary References....................85

S1. Experimental Section

1.1 Materials

Chitosan (degree of deacetylation 90%) was purchased from Ruji Biotechnology Co., Ltd., Zinc nitrate hexahydrate was obtained from damao (99.8%, $Zn(NO_3)_2 \cdot 6H_2O$, Tianjin, China), nano-Zn was purchased from Titan Technology Co., Ltd., (AR, Shanghai, China). Some substrates for alcohols and nitroarenes such as 3-chloronitrobenzene, 4-methoxybenzyl alcohol, 3-methoxybenzyl and 2-methoxybenzyl alcohol *etc.* were used as received. All other reagents, such as ethyl acetate, petroleum ether, ethanol, *etc.* were obtained from a variety of commercial sources and could be used without further purification.

1.2 Characterization

The morphology and EDX mapping of the samples were examined using a field emission scanning electron microscope (FESEM, Zeiss SUPRA 55 Sapphire, Germany) at an accelerating voltage of 5 kV. Nitrogen adsorption measurements were performed using a Micromeritics AsAp2020 (USA), and Brunauer-Emmett-Teller (BET) and Barrett-Joyner-Halenda (BJH) analyses were conducted automatically. The BJH analysis was performed from the desorption branch of the isotherm. X-ray diffraction (XRD) patterns were recorded using an X-ray powder diffractometer (Rigaku Miniflex600, Japan) with Cu Kα radiation (λ = 1.5406 Å). Infrared spectra were obtained using a Fourier-transform infrared spectrometer (FT-IR, PerkinElmer Corporation/model 1600, USA). X-ray photoelectron spectroscopy (XPS) was collected using a VG Multi Lab 2000 system equipped with a monochromatic Al Kα

X-ray source (XPS, VG Multi Lab 2000, USA). Transmission electron microscopy (TEM) images were collected using a JEM-2010 electron microscope (JEOL, Japan) at an accelerating voltage of 200 kV. HAADF-STEM and elemental mappings of HAADF-STEM images were investigated by a Titan Themis Z (FEI) operated at an acceleration voltage of 300 kV. The loading of Zn was performed on an ICP-OES (Prodigy 7, Leeman Labs Inc., U. S. A.). The X-ray absorption fine structure spectra (Zn K-edge) were collected at BL14W beamline in Shanghai Synchrotron Radiation Facility (SSRF). The storage ring of SSRF was operated at 3.5 GeV with a stable current of 200 mA. All spectra were collected in ambient conditions. The Zn K-edge XANES date were recorded in a transmission mode. Zn foil, ZnO and ZnPc were used as references. The acquired EXAFS date was extracted and processed according to the standard procedures using the ATHENA module implemented in the IFEFFIT software packages. Gas chromatography (GC) yields were determined using a Varian GC 3900 gas chromatograph (GC, Varian GC 3900, USA) with an FID detector. The ^{1}H NMR spectra were recorded at ambient temperature at 300.1 and 400.1 MHz on Bruker AVANCE 300 and 400 spectrometers, respectively, using $CDCl_3$ (7.26 ppm) as the internal standard. The ^{13}C NMR spectra were obtained at 75 or 100 MHz, referenced to the internal solvent signal ($CDCl_3$, central peak at 77.16 ppm). Chemical shifts and coupling constants (J) are reported in ppm and Hz, respectively. Peak multiplicities are denoted as follows: s (singlet), d (doublet), t (triplet), m (multiplet).

Supplementary Figures

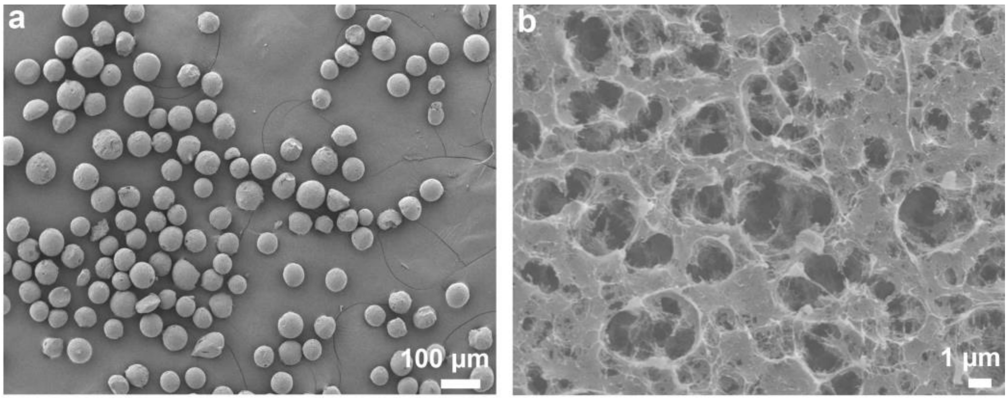

Figure S1. SEM images of the chitosan. SEM image of the initial/pure chitosan (a). Partial enlarged view of individual initial/pure chitosan (b).

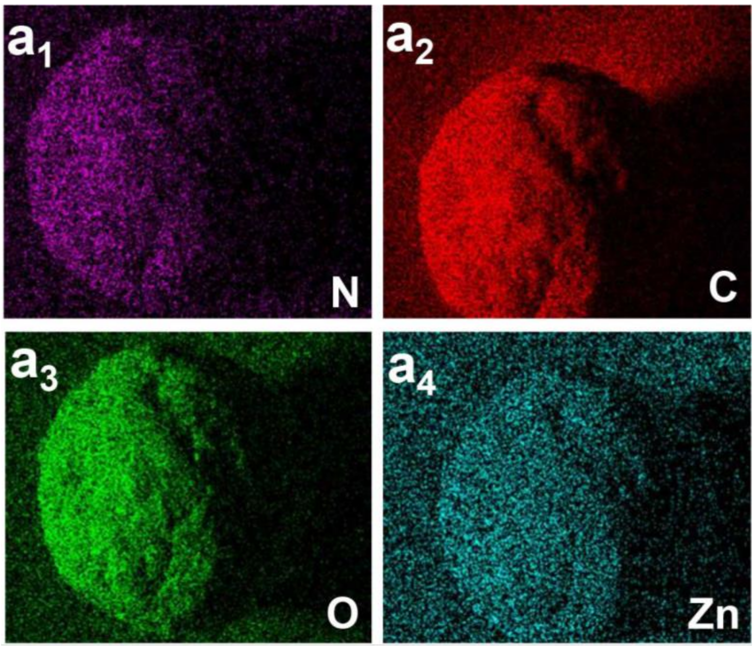

Figure S2. SEM-EDX mapping images of various elements in Zn/CS catalyst. N element (a_1), C element (a_2), O element (a_3), and the Zn element (a_4).

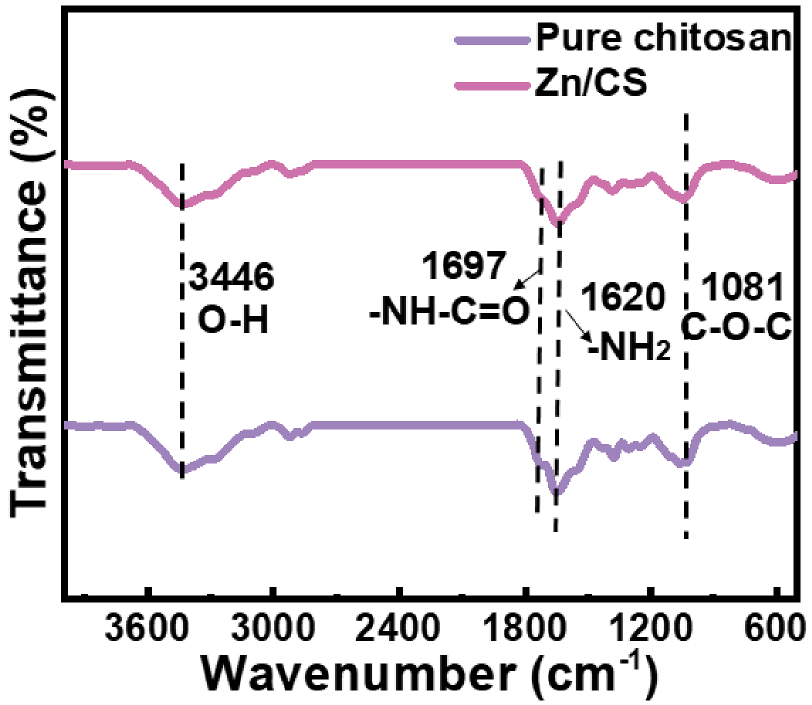

Figure S3. FT-IR spectra of the pure chitosan and Zn/CS.

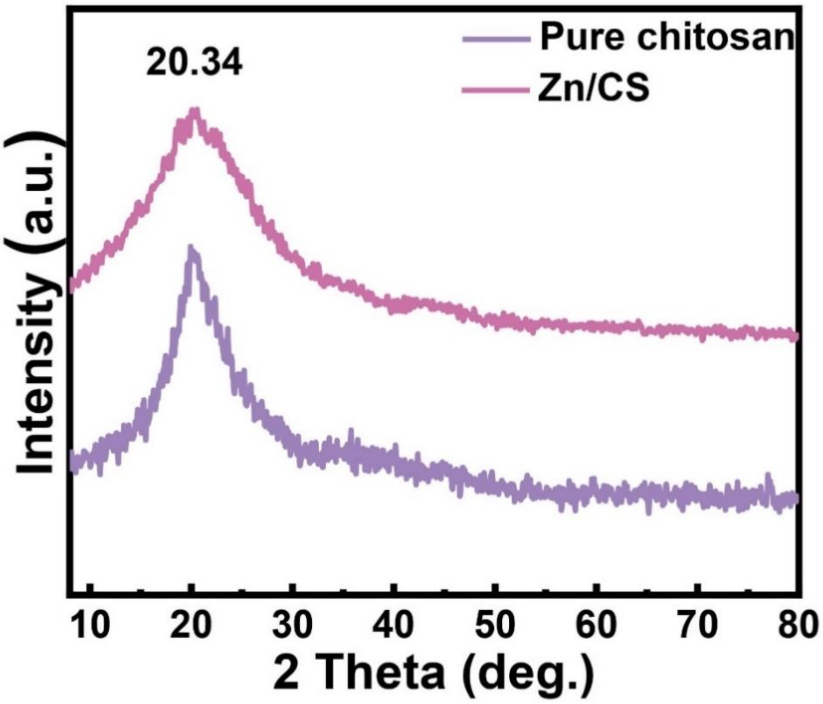

Figure S4. XRD spectra of the pure chitosan and Zn/CS.

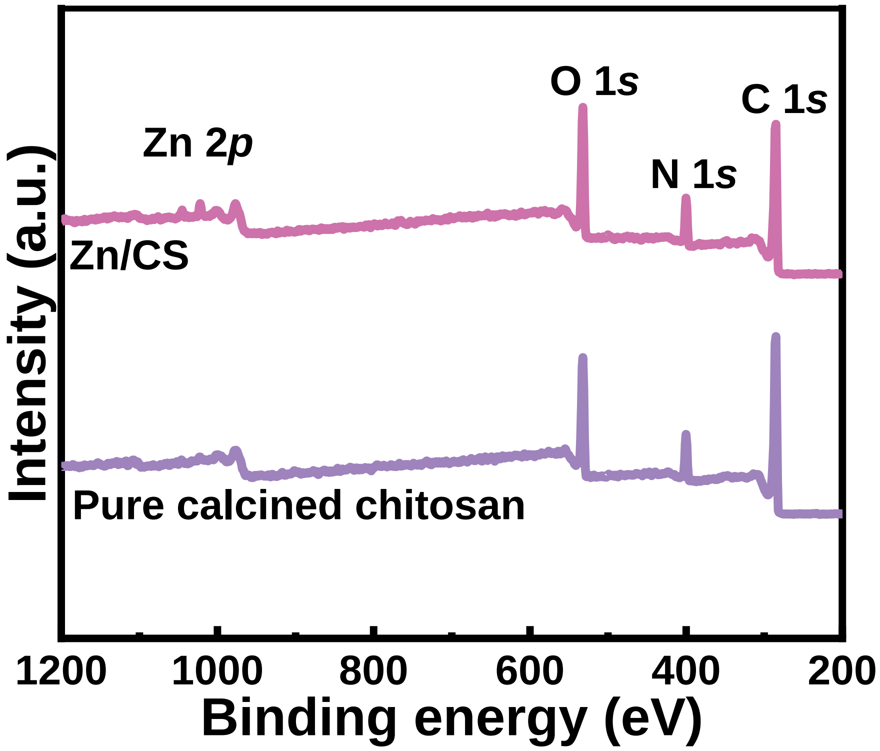

Figure S5. XPS full-size spectra of the pure calcined chitosan and Zn/CS.

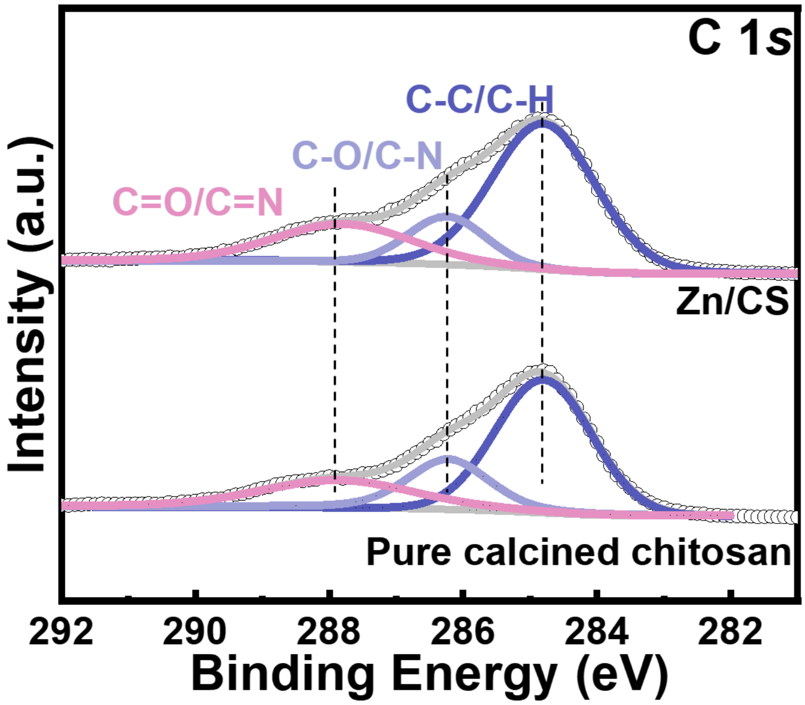

Figure S6. XPS of C1*s* spectra for the pure calcined chitosan and Zn/CS.

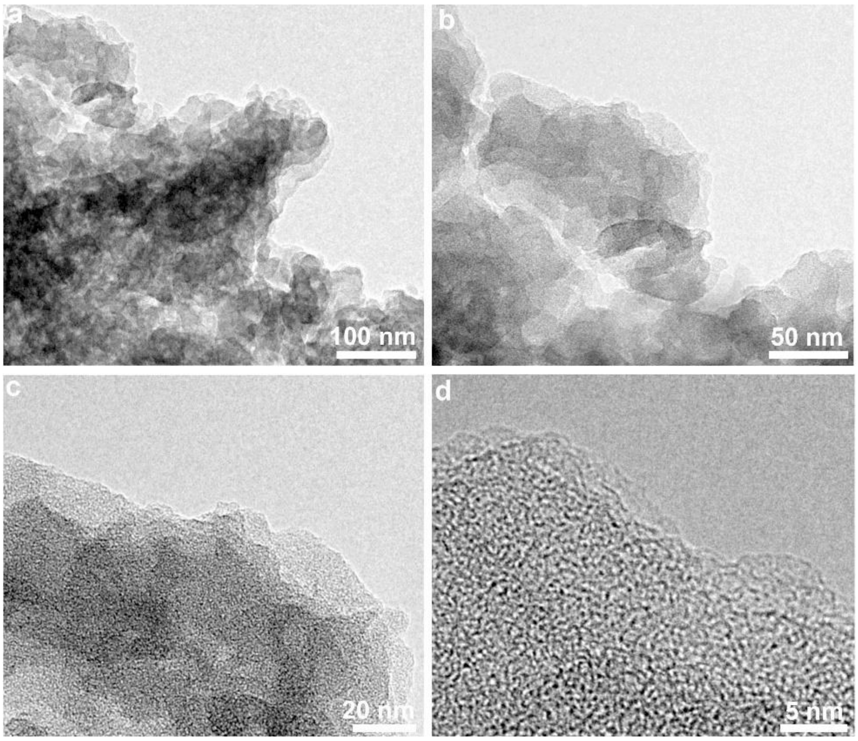

Figure S7. TEM images of Zn/CS at different magnifications. Scale bars: 100 nm (a), 50 nm (b), 20 nm (c), and 5 nm (d).

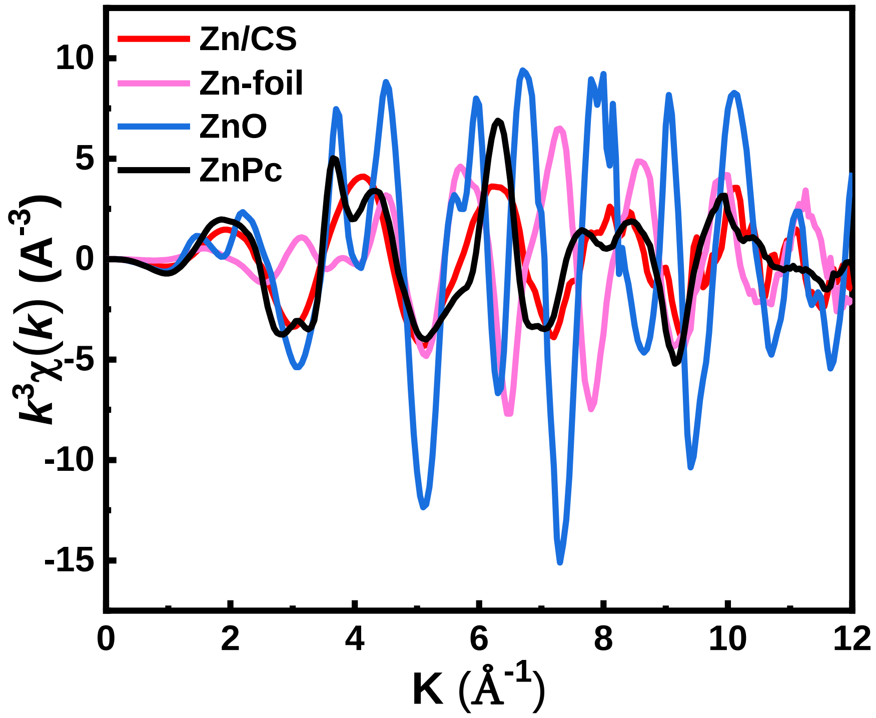

Figure S8. The k^2-weighted EXAFS in k-space at the Zn K-edge for the Zn/CS, Zn foil, ZnO and ZnPc.

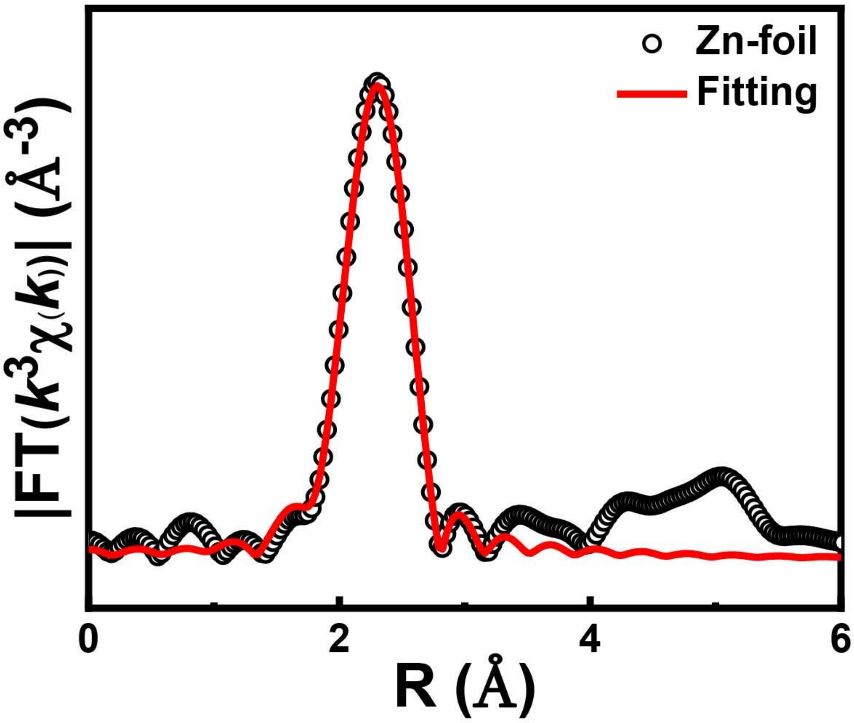

Figure S9. A typical fitting curve of the EXAFS signal in R-space for the adsorbed Zn foil.

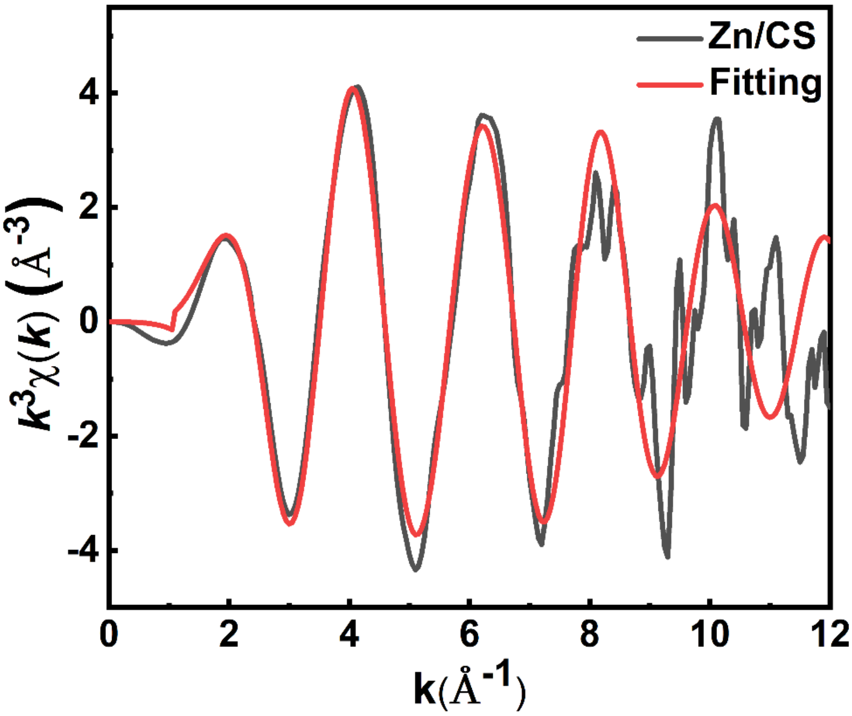

Figure S10. Zn K-edge EXAFS fitting curves of Zn/CS at K space.

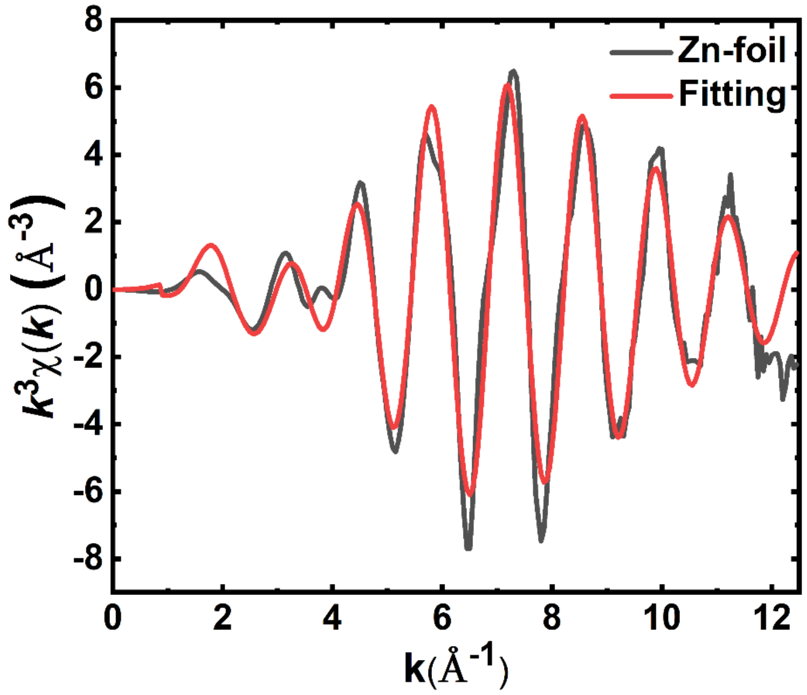

Figure S11. Zn K-edge EXAFS fitting curves of Zn foil at K space.

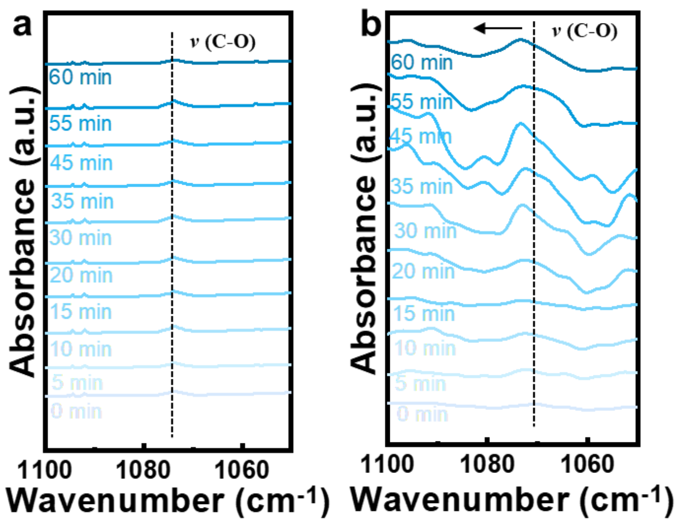

Figure S12. **Time-resolved DRIFTS spectra**. The benzyl alcohol oxidation process with the addition of Zn/CS catalyst without KOH (a), and Zn/CS catalyst with KOH (b).

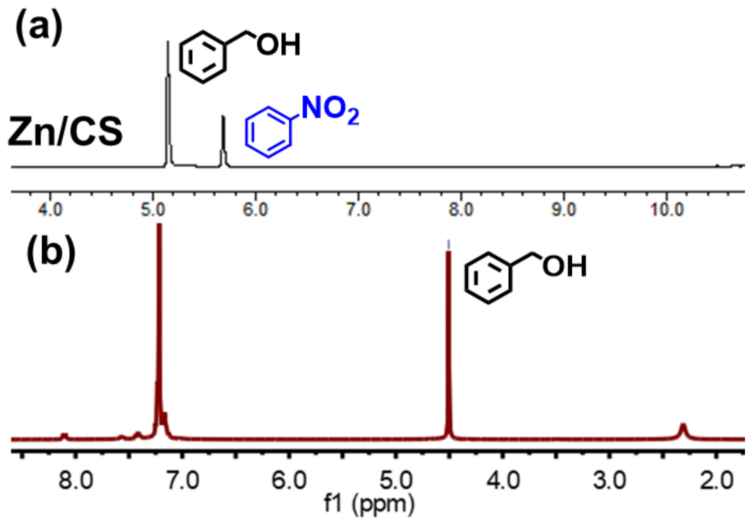

Figure S13. **GC and ^{1}H NMR spectra**. GC (a) and ^{1}H NMR (b) spectra of the borrowing hydrogen reaction between nitrobenzene and benzyl alcohol in the presence of Zn/CS catalyst without KOH.

Time-resolved diffuse reflectance infrared Fourier transform spectroscopy (DRIFTS) in Figure S12 showed that in the absence of KOH, the C-O stretching vibration peak of benzyl alcohol (1073 cm^{-1}) adsorbed on the Zn/CS catalyst showed negligible change over the course of 1 h. In contrast, the peak shifted significantly in the presence of KOH (Figure S12b), which was consistent with the formation of alkoxide intermediates (*J. Catal.* **2024**, *434*, 115537; *Appl. Catal. B Environ*. **2022**, *319*, 121904). This shift directly supports the hypothesis that the benzyl alcohol undergoes deprotonation under basic conditions. The comparison clearly demonstrates that the activation of benzyl alcohol is strongly dependent on the presence of an external base.

To further substantiate these findings, we conducted GC and ^{1}H NMR analyses. As shown in Figure S13, after 3 h of reaction, only the characteristic peaks of the starting substrates (benzyl alcohol and nitrobenzene) were detected, with no intermediate or oxidation product signals observed in the absence of KOH. These data confirm that the Zn/CS catalyst does not achieve the deprotonation and the subsequent conversion of benzyl alcohol in the absence of base.

Entry	Catalyst	Time (h)	Yield (%)
1	Zn/CS	21	98
2	Zn/C	21	8
3	Nano-Zn	21	8
4	$ZnCl_2$	21	7
5	$Zn(NO_3)_2 \cdot 6H_2O$	21	16
6	Chitosan	21	None

Figure S14. Summarized data of N-benzylaniline yield with reaction time in the borrowing hydrogen of nitrobenzene and benzyl alcohol for various catalysts.

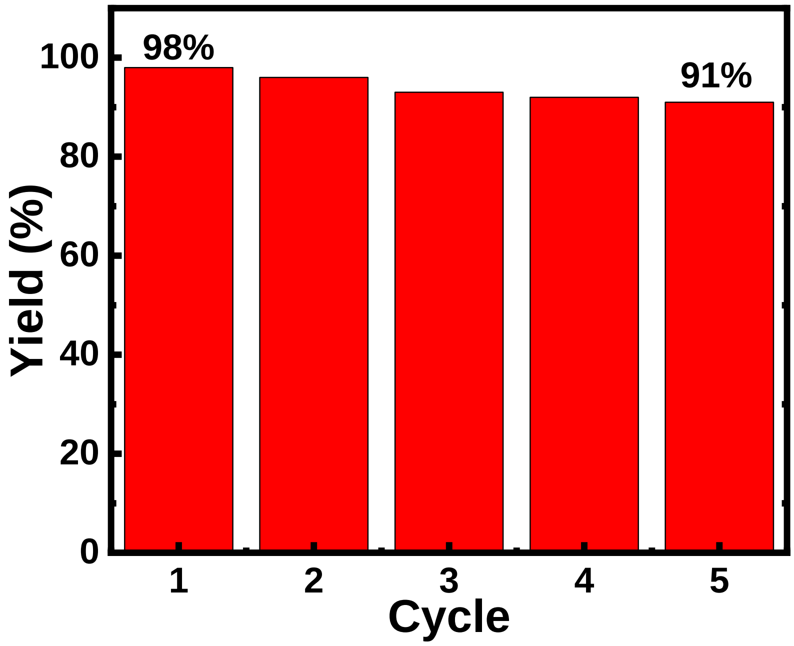

Figure S15. The yields of the target product for Zn/CS during 5 cycles.

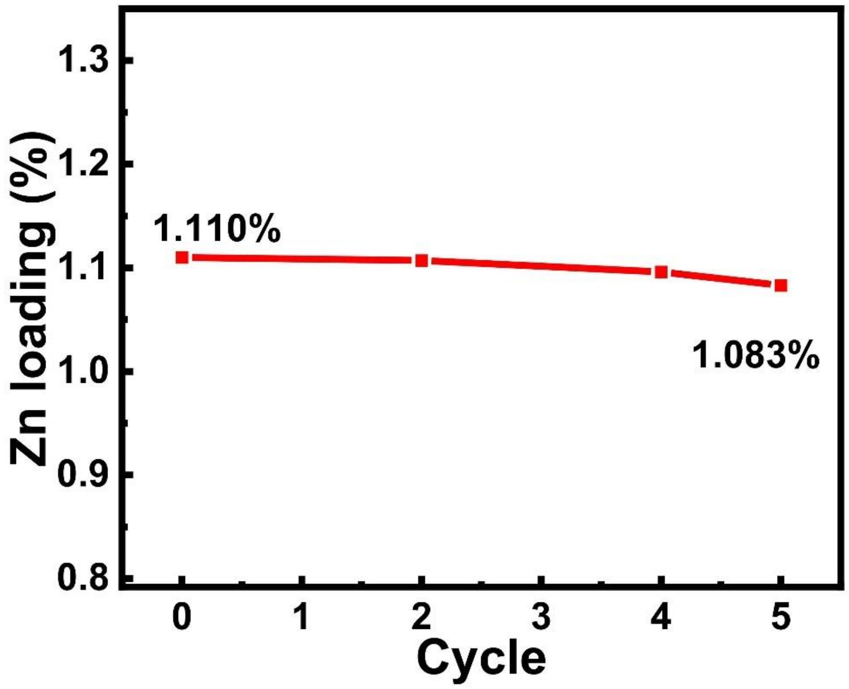

Figure S16. The Zn contents in Zn/CS during 5 cycles tested by ICP-OES.

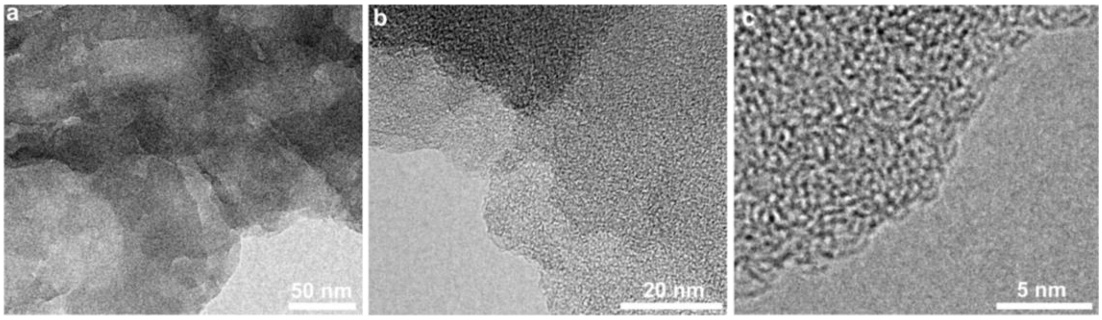

Figure S17. TEM images of the 5-reused Zn/CS catalyst. Scale bars: 50 nm (a), 20 nm (b), and 5 nm (c).

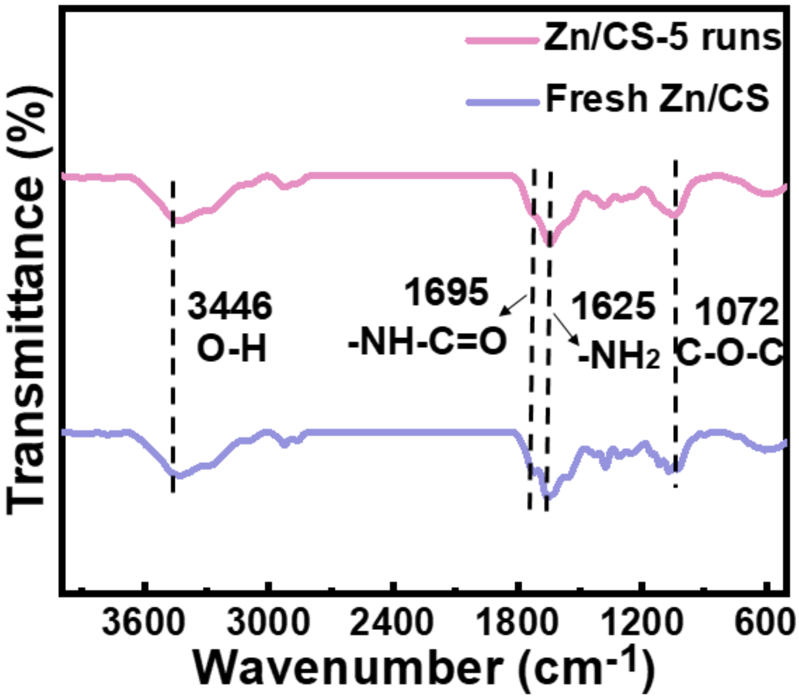

Figure S18. FT-IR spectra of the 5-reused Zn/CS catalyst.

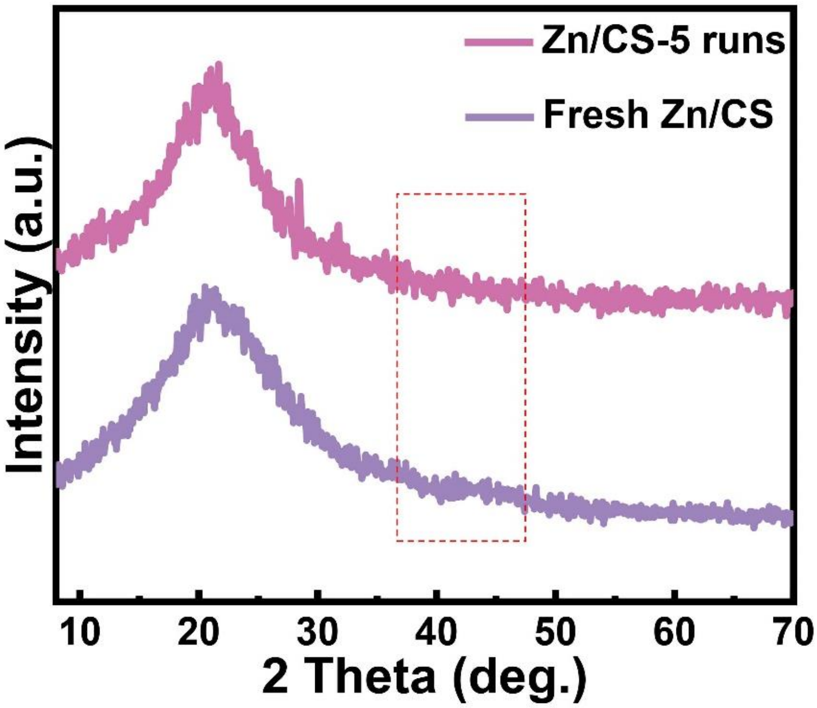

Figure S19. XRD patterns of the 5-reused Zn/CS catalyst.

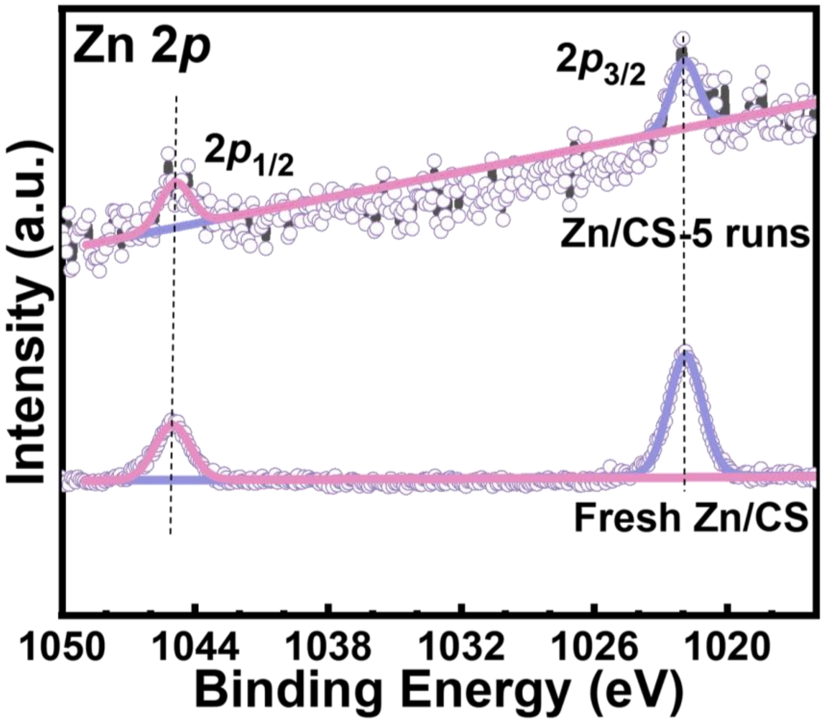

Figure S20. XPS spectra of the fresh Zn/CS and the 5-reused Zn/CS.

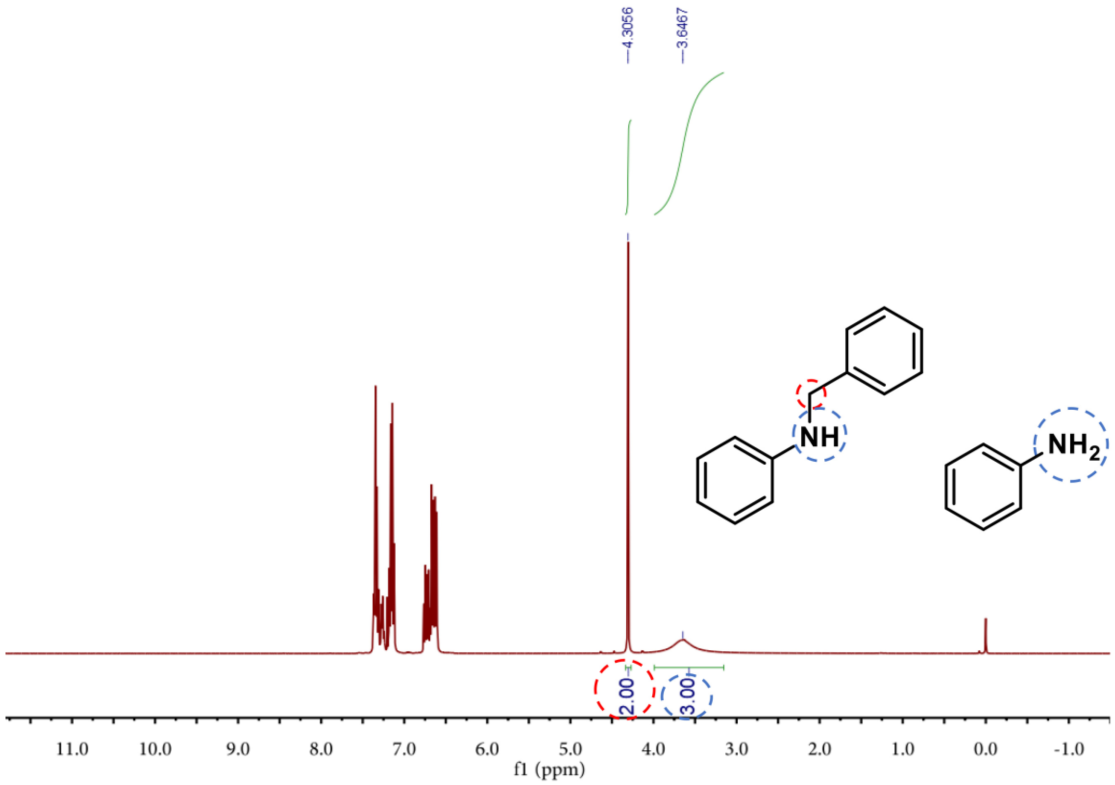

Figure S21. ^{1}H NMR spectrum of aniline and N-benzylaniline mixture in $CDCl_3$.

Deuterium labelling experiments

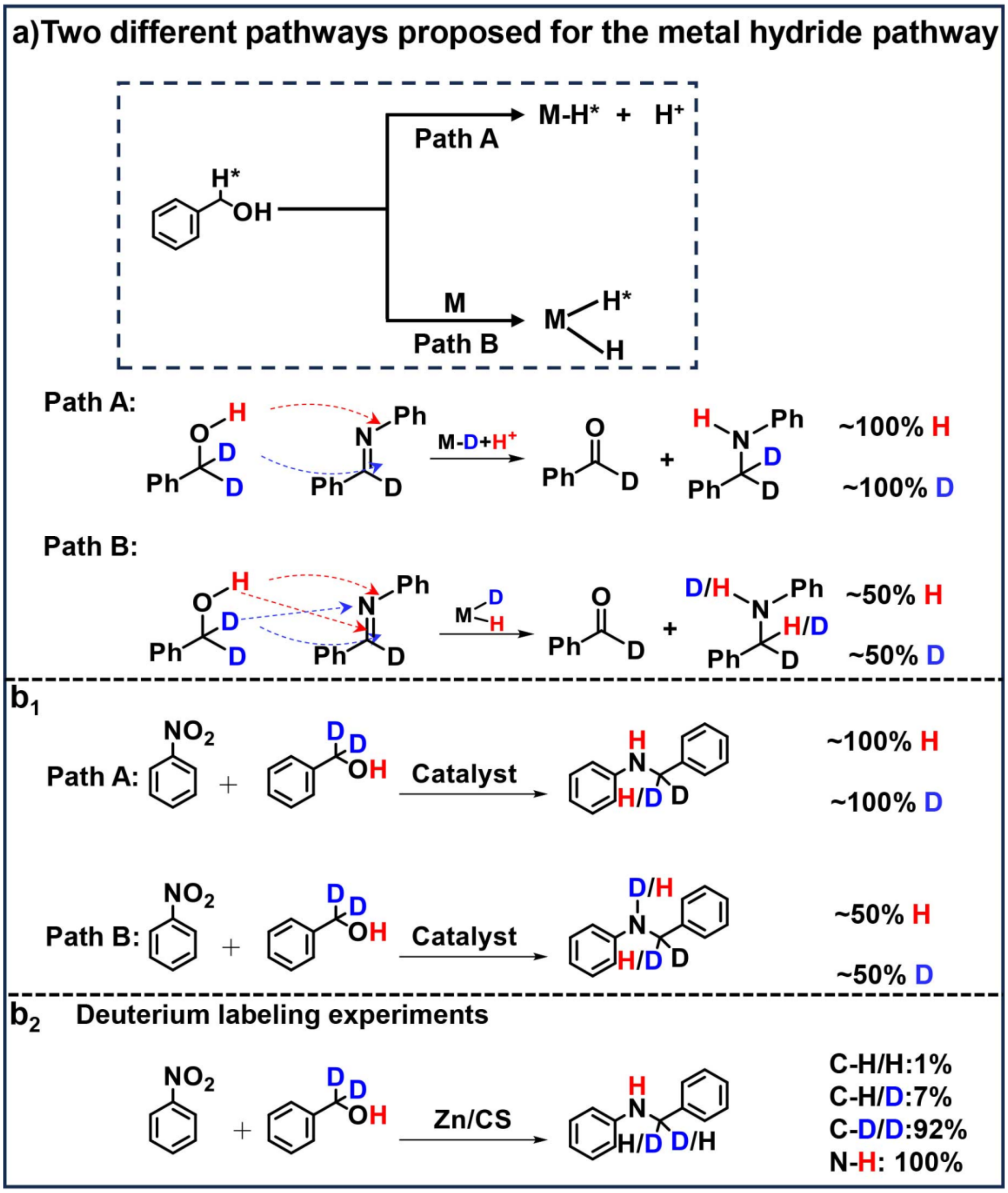

Figure S22. **Deuterium labelling experiments**. Two different pathways proposed for the metal hydride pathway (a). The possible reaction pathways for our Zn/CS catalyst (b_1). The real reaction pathway for our Zn/CS catalyst (b_2).

Based on the literature[1], two commonly reported mechanisms in hydrogen borrowing/hydrogen transfer reactions were proposed: the monohydride pathway (M-H, path A) and the dihydride pathway (M-H_2, path B) (Figure S22a). In path A, the benzyl alcohol underwent deprotonation in the presence of base (KOH) and then

connected with the metal particle. The metal particle was further connected with the α-C-H in Ar-CH_2-O^- to form M-H. In path A, the target product tended to be Ar-NH-CD/D-Ar. In contrast, Path B involved the O-H and α-C-H of the alcohol interacting with the metal sites to generate the M-H_2 species, which was more likely to produce the target product of Ar-NH/D-CH/D-Ar[2-4] (Figure S22b_1).

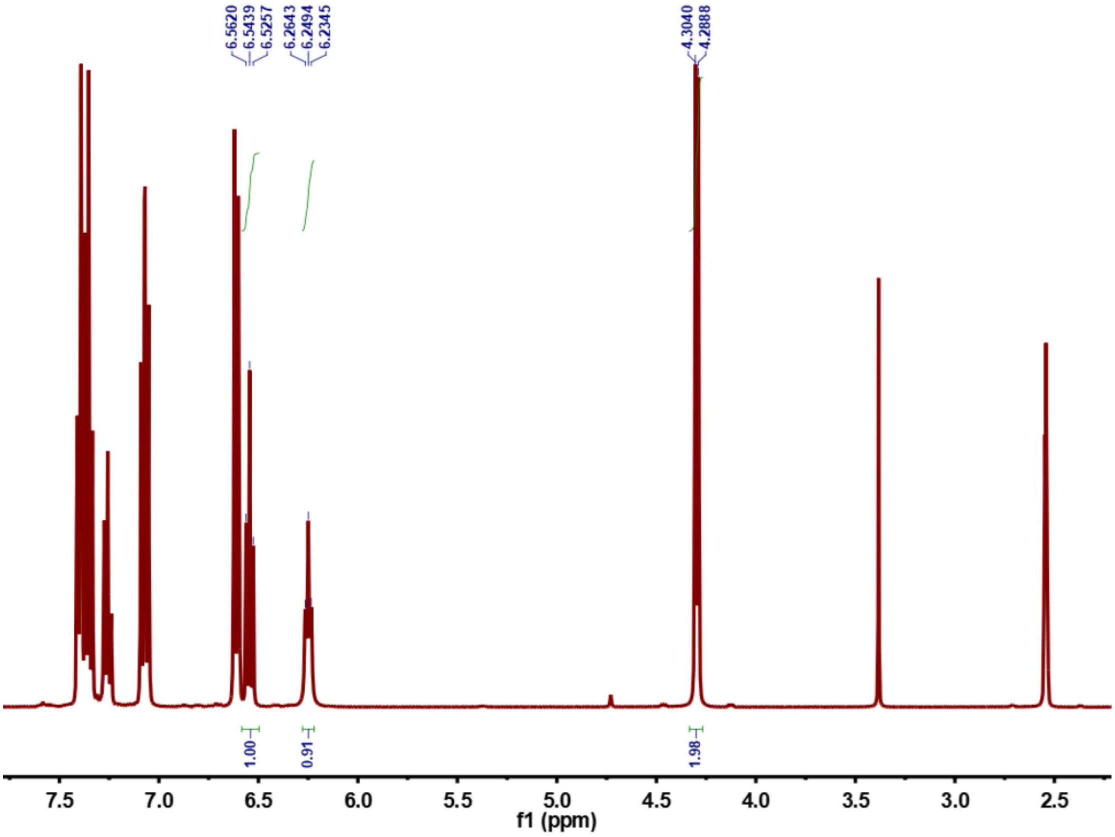

Figure S23. ^{1}H NMR spectrum of N-benzylaniline with type of C-H/H in DMSO.

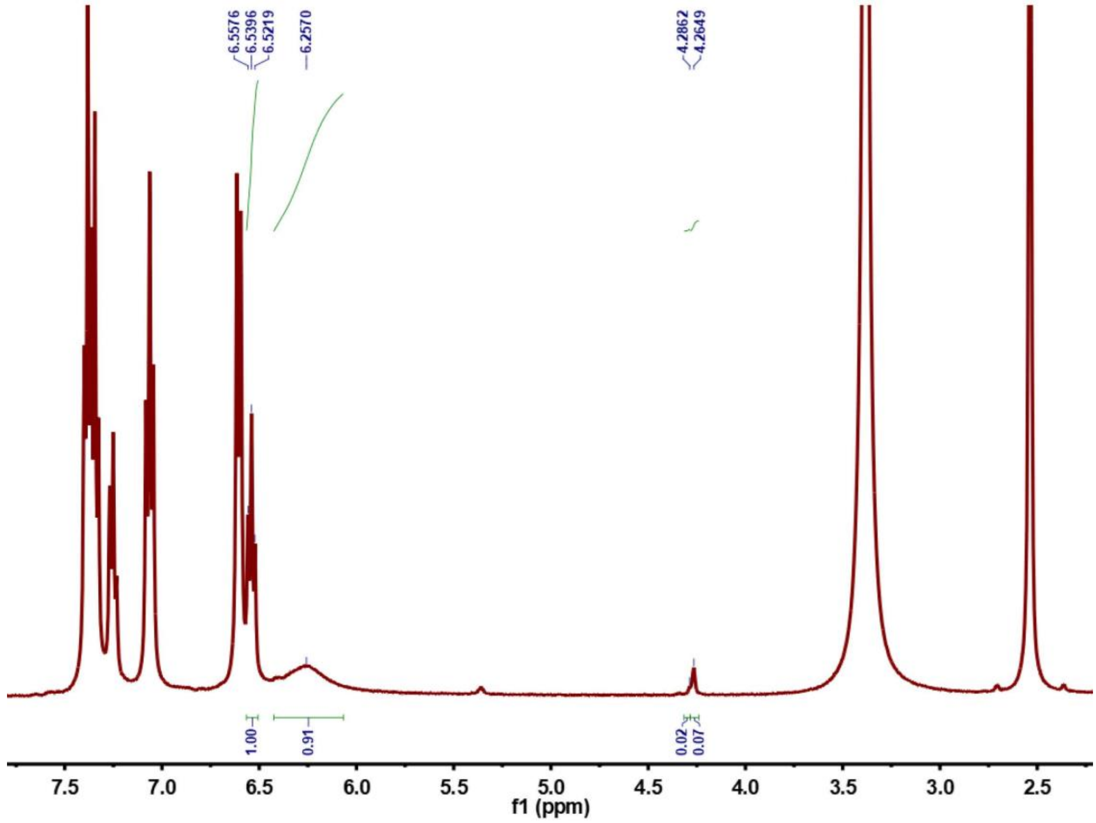

Figure S24. ^{1}H NMR spectrum of N-benzylaniline with types of C-H/H, C-H/D and C-D/D in DMSO.

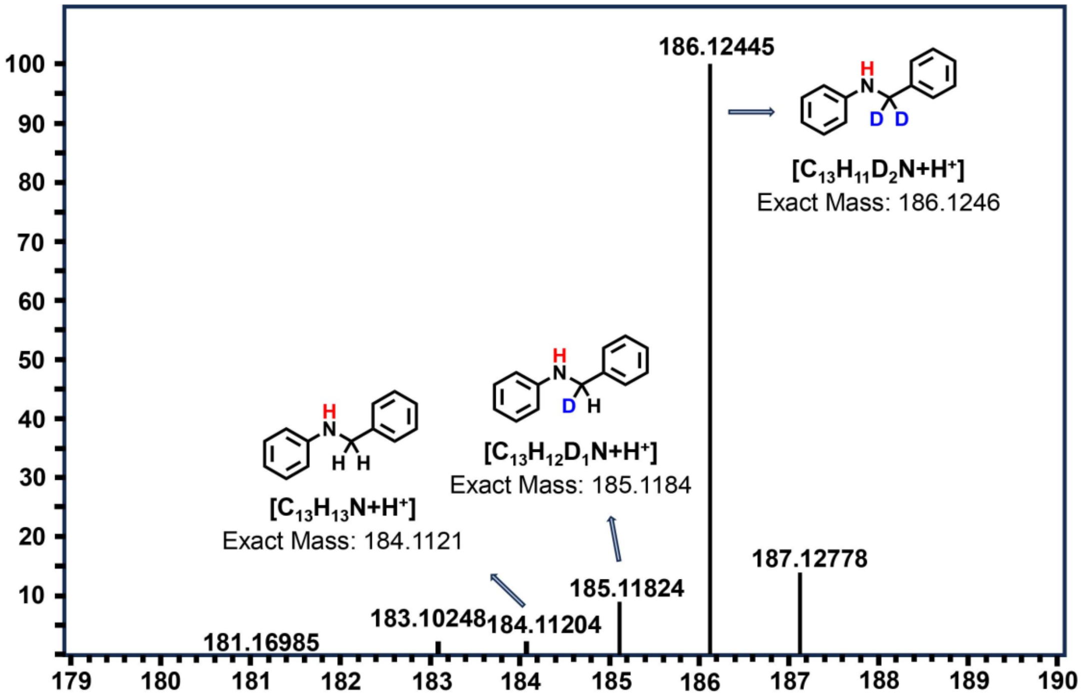

Figure S25. HR-MS spectrum for the deuterium labelling experiment by using α-C-H deuterated benzyl alcohol.

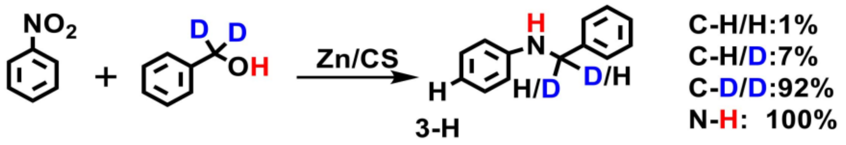

	3-H	C-H/H	C-H/D	C-D/D	N-H
Singnal δ	6.54	4.28	4.26	-	6.25
Integral value	1	0.02/1.98	0.07/1.98	-	0.91/0.91
Calculated ratio	-	1%	7%	92%	100%

Figure S26. Summary of the deuterium labelling experiments by using α-C-H deuterated benzyl alcohol.

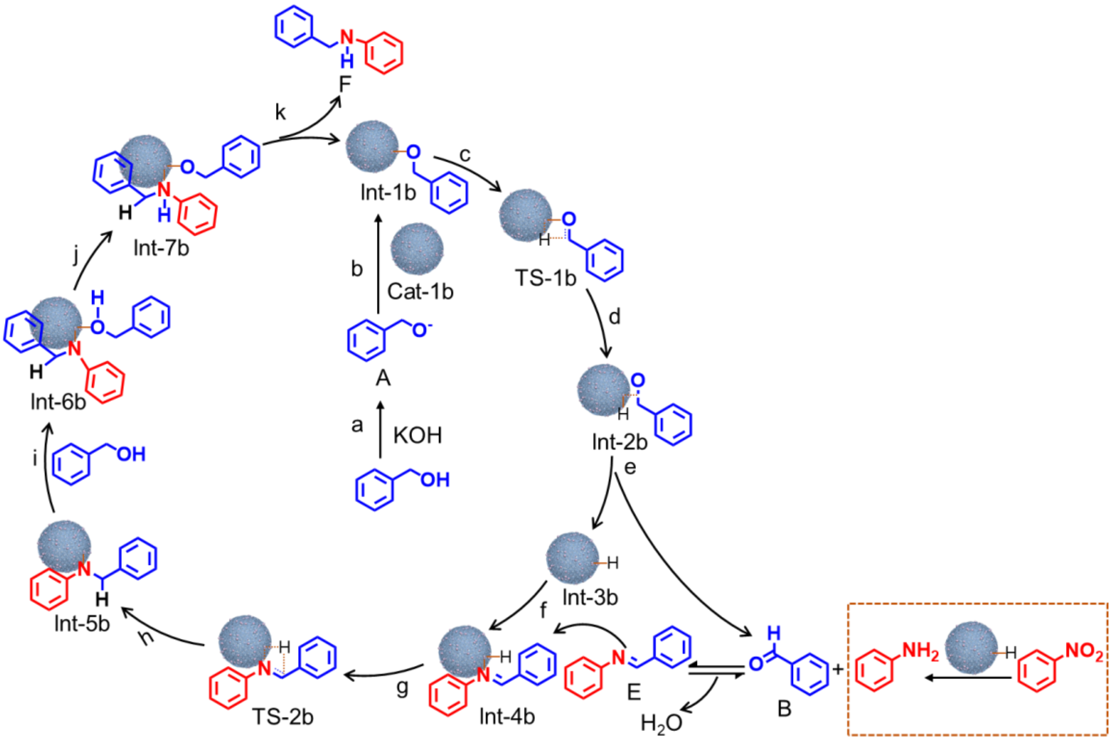

Figure S27. The proposed mechanism for the borrowing hydrogen reaction of nitroarenes with alcohols catalyzed by Zn/CS.

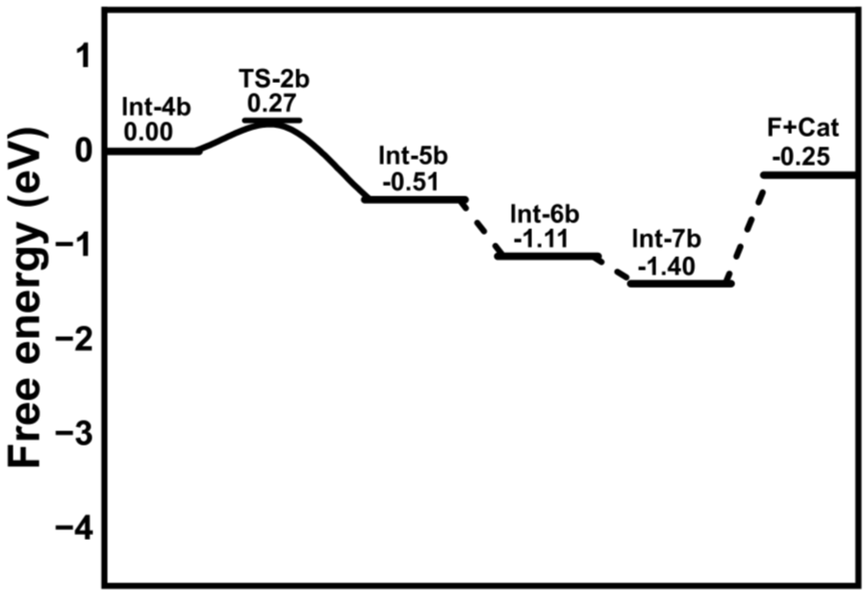

Figure S28. The reaction pathway and corresponding Gibbs free energy profiles for hydrogenation of imine by using the Zn/CS catalyst.

The changes in Gibbs free energy during the hydrogenation of imine were also be investigated. As shown in Figures S27-28, the imine (E) first adsorbed onto the intermediate of lnt-3b, forming the intermediate of lnt-4b (step f). Subsequently, the Zn-H in lnt-3b further combined with the C atom in C=N bond of imine to form the transition state of TS-2b, and the energy barrier that needed to be overcome was 0.27 eV (step g). Then, the TS-2b was hydrogenated to the intermediate of lnt-5b. In subsequent steps, lnt-5b continued to react with benzyl alcohol to form intermediates of lnt-6b and lnt-7b, ultimately producing the product of N-benzylaniline (F).

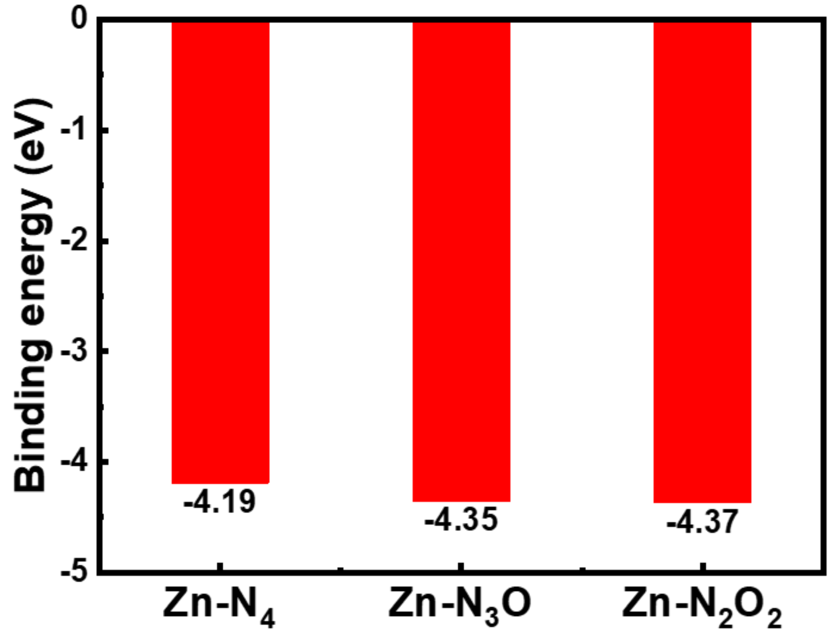

Figure S29. Coordination formation energy of ZnN_4-CS, ZnN_3O-CS, and ZnN_2O_2-CS.

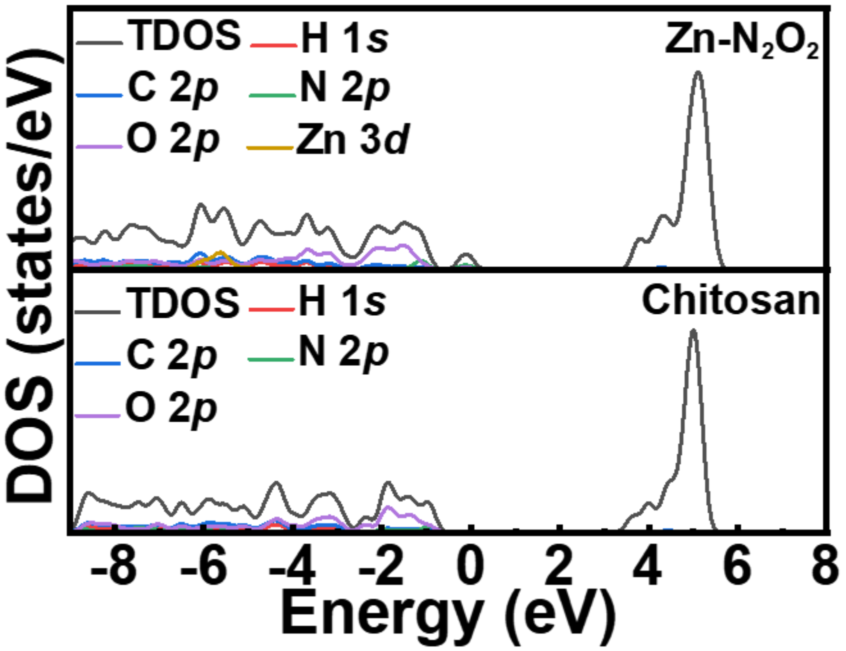

Figure S30. The enlarged image of density of states for the blank chitosan and ZnN_2O_2.

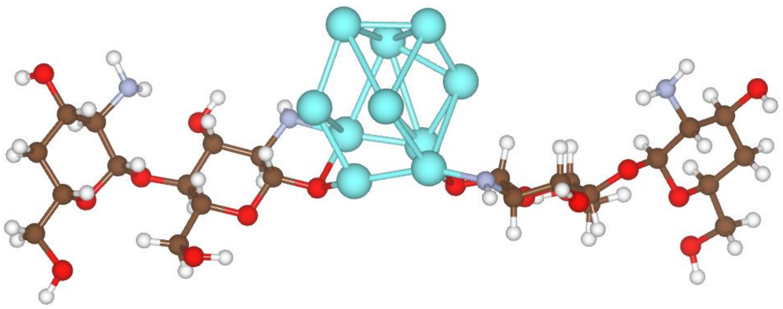

Figure S31. The optimal model of nano-Zn/CS clusters.

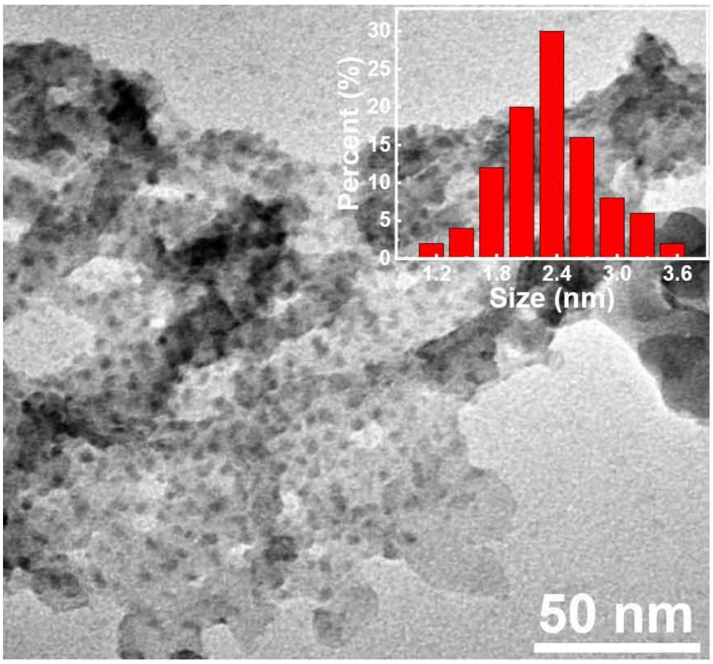

Figure S32. TEM image and particle size distribution of the nano-Zn/CS catalyst.

The nano-Zn/CS exhibited nanoscale structural feature, with an average Zn nanoparticle size of 2.36 nm. The preparation of the nano-Zn/CS catalyst was as follows: By employing the impregnation method, 28 mg of the $Zn(NO_3)_2 \cdot 6H_2O$ was dissolved in 5 mL of H_2O. Subsequently, 200 mg of the previously prepared chitosan microspheres were dispersed in 200 mL of H_2O and soaked for 15 min. The $Zn(NO_3)_2 \cdot 6H_2O$ solution was then added dropwise to the chitosan microsphere

suspension, which was stirred in an ice bath for 1 h, followed by continued stirring at room temperature for 3 h. Subsequently, the resulting mixture was reduced by excess $NaBH_4$ at room temperature, filtered and lyophilized for further use. Finally, the above catalyst was activated at 250 °C (with a heating rate of 2 °C/min) under an argon (Ar) atmosphere for 2 h to produce the nano-Zn/CS catalyst.

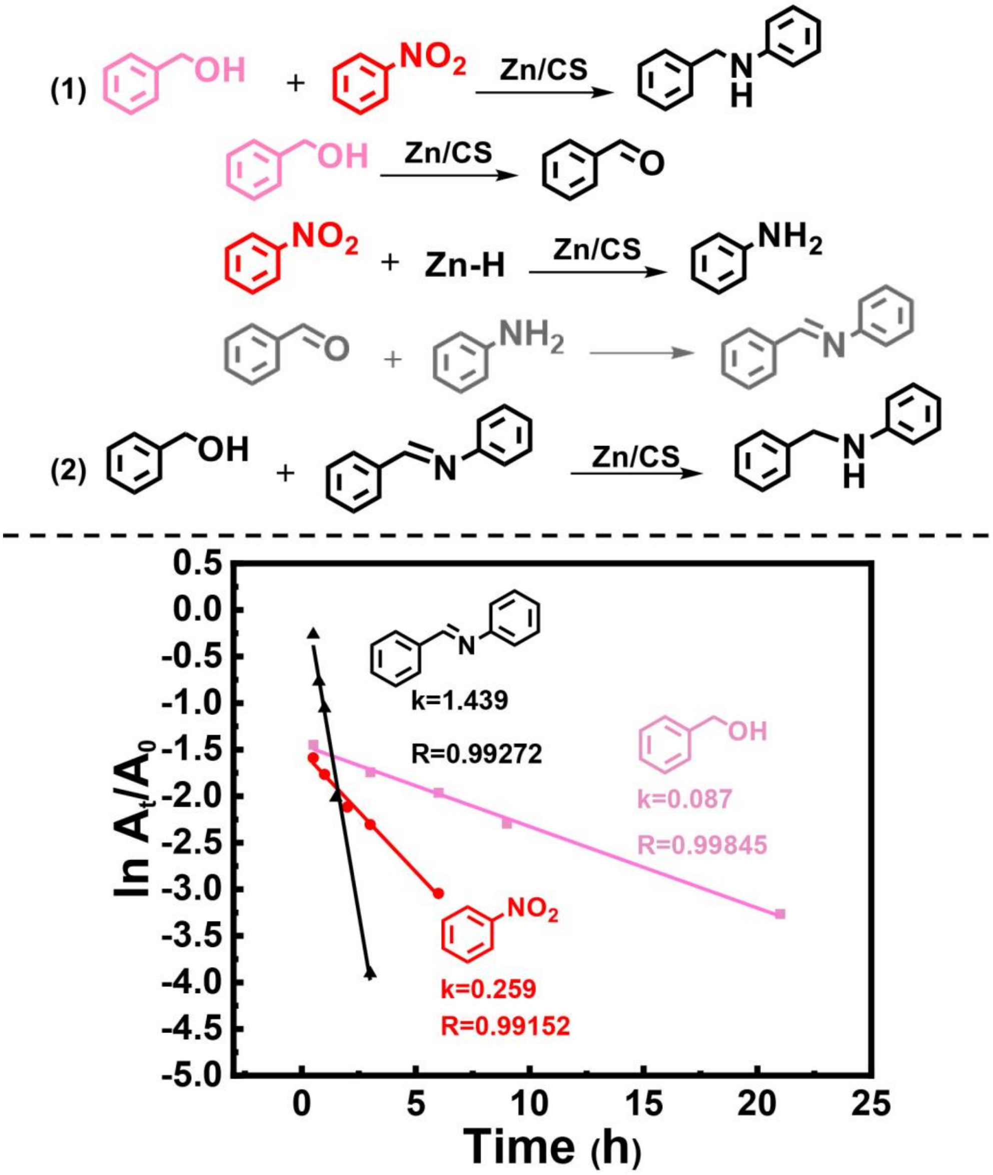

Figure S33. The kinetic plots for nitrobenzene, benzyl alcohol, and N-benzylideneaniline. A_0: original concentration of the substrate. A_t: concentration of the substrate at time t. k: rate constant.

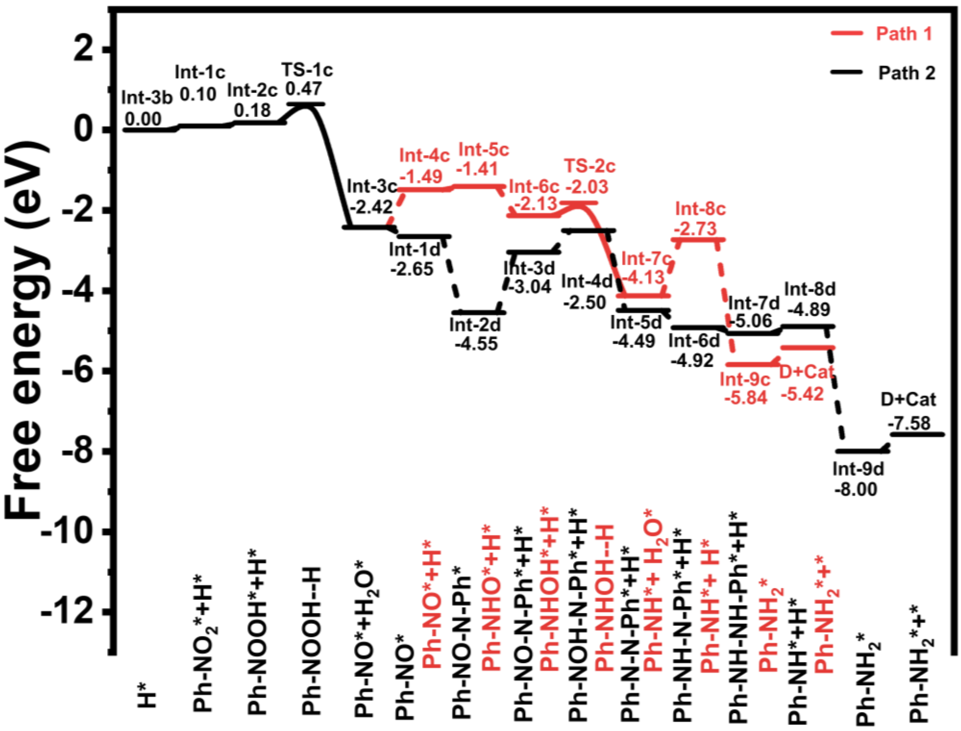

Figure S34. The proposed mechanism for the nitrobenzene reduction to aniline catalyzed by Zn/CS, and the corresponding changes of Gibbs free energy.

The changes in Gibbs free energy during the hydrogenation of nitrobenzene were also be investigated. As shown in above (Path 1), the H* represented the Zn/CS-H (i.e., the lnt-3b in our manuscript). Firstly, the $-NO_2$ in nitrobenzene connected with the Zn-H in lnt-3b to form intermediates lnt-1c and lnt-2c. Then, the H atom in Zn-H of lnt-3b was added to nitrobenzene to form the transition state of TS-1c, which required overcoming an energy barrier of 0.29 eV. Subsequently, after the dehydration reaction from TS-1c, intermediate of Ph-NO* (lnt-3c) was formed, which then connected with lnt-3b to form intermediate of lnt-4c. The intermediate of lnt-4c underwent hydrogenation with the H atom in Zn-H of lnt-3b to form intermediate of lnt-5c, then leading to the formation of intermediate Ph-NHOH* (lnt-6c). The intermediate of lnt-6c was then adsorbed onto Zn-H of lnt-3b to form the transition state of TS-2c (with overcoming an energy barrier of 0.10 eV), which was further

underwent dehydration to form the intermediate of lnt-7c. Finally, the lnt-7c connected with Zn-H in lnt-3b to form intermediate of lnt-8c, which was further hydrogenated to form intermediate of lnt-9c, ultimately yielding the product aniline.

Since we observed the presence of azobenzene (Ph-N=N-Ph) in the reaction mixture (Figure 4b in the manuscript) in the GC spectra, therefore, the proposed Path 2 was presented in below. The intermediates of lnt-3c led to the formation of the intermediate lnt-1d, accompanied by the elimination of water. Subsequently, the lnt-1d reacted with the N-phenylhydroxylamine* (Ph-NHOH*) intermediate to yield lnt-2d. The intermediate lnt-2d could bound with Zn-H in lnt-3b to form intermediate of lnt-3d and lnt-4d. The lnt-4d further underwent hydrogenation and dehydration to form intermediate of lnt-5d. The intermediate of lnt-5d was sequentially hydrogenated to form lnt-6d and lnt-7d, with lnt-7d further converting into lnt-8d. Finally, the lnt-8d was hydrogenated to yield the final product, aniline ($Ph-NH_2$).

It could be seen that although both paths can produce the target product, the ΔG (Gibbs free energy change) is more advantageous for Path 2, proving that Path 2 may be the dominant reaction pathway. Meanwhile, the ^{1}H NMR and GC kinetic spectra demonstrate the formation of substantial amounts of azobenzene (Ph-N=N-Ph) as the reaction progressed compared to the trace phenylhydroxylamine (Ph-NHOH) intermediate, further indicating that Path 2 is the main reaction pathway.

Supplementary Tables

Table S1. Pore volume, specific surface area and average pore size of pure chitosan and Zn/CS.

**Sample**	**Pore volume (cm^3/g)**	**Surface area (m^2/g)**	**Pore size (nm)**
Pure chitosan	0.717	186.36	3.657
Zn/CS	0.618	194.07	3.676

Table S2. EXAFS fitting parameters at the Zn K-edge of Zn/CS and Zn foil（S_0^2=0.98 from Zn-foil）

	shell	CN^a	R^b(Å)	σ^{2c}($Å^2$)	ΔE_0^d(eV)	R factor
Zn-foil	Zn-Zn	6	2.65±0.01	0.0107	2.78±0.63	0.00203
	Zn-Zn	6	2.80±0.1	0.0235		
Zn/CS	Zn-N/O	4.42±0.26	1.99±0.02	0.00494	4.4±1.5	0.00713

^{a}*CN*: coordination numbers; ^{b}*R*: bond distance; $^c\sigma^2$: Debye-Waller factors; $^d\Delta E_0$: the inner potential correction. R factor: goodness of fit. Error bounds that characterize the structural parameters obtained by EXAFS spectroscopy were estimated as CN±20%; R ± 1%; σ^2 ± 20%.

Table S3. Conditional screening of nitrobenzene and benzyl alcohol [a]

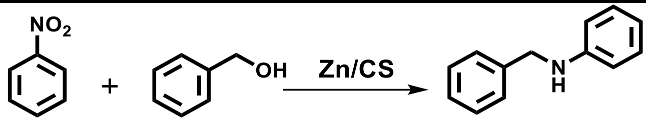

Entry	Solvent	Temperature (°C)	Base	Time (h)	Yield (%) [b]
1	DMF	120	KOH	24	NR
2	DMSO	120	KOH	24	NR
3	EtOH	120	KOH	24	Trace
4	H_2O	120	KOH	24	NR
5	CH_3CN	120	KOH	24	NR
6	EtOAc	120	KOH	24	NR
7	DCM	120	KOH	24	4
8	Toluene	120	KOH	24	32
9	*N*-heptane	120	KOH	24	62
10	*N*-hexane	120	KOH	24	94
11	Petroleum ether	120	KOH	24	95
12	Petroleum ether	120	LiOH	24	75%
13	Petroleum ether	120	Cs_2CO_3	24	Trace
14	Petroleum ether	120	KOtBu	24	28
15	Petroleum ether	120	Et_3N	24	NR
16	Petroleum ether	120	K_2CO_3	24	Trace
17	Petroleum ether	120	NaOH	24	37
18	Petroleum ether	120	$NaHCO_3$	24	Trace
19	Petroleum ether	120	KOH	15	87
20	Petroleum ether	120	KOH	18	92
21	Petroleum ether	120	KOH	21	98

[a] Reaction conditions: nitrobenzene (0.2 mmol), benzyl alcohol (0.6 mmol), base (0.54 mmol), solvent (6 mL), Zn/CS (0.09 mol% [Zn], Zn: $PhNO_2$), reacted at 120 °C for 24 h. [b] The yield of N-benzylaniline was determined by gas chromatography.

Table S4. Effect of reaction temperature on borrowing hydrogen reaction of nitrobenzene and benzyl alcohol [a]

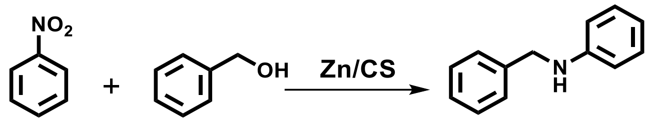

Entry	Temperature (℃)	Yield (%) [b]
1	90	22
2	100	47
3	110	83
4	120	98
5	130	92

[a] Reaction conditions: nitrobenzene (0.2 mmol), benzyl alcohol (0.6 mmol), KOH (0.54 mmol), petroleum ether (6 mL), Zn/CS (0.09 mol% [Zn], Zn: $PhNO_2$), reacted for 21 h. [b] Yield was measured by GC.

Table S5: Effect of base dosage on borrowing hydrogen of nitrobenzene and benzyl alcohol [a]

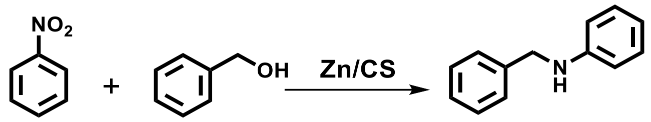

Entry	KOH (mmol)	Yield (%) [b]
1	0.27	Trace
2	0.36	16
3	0.45	29
4	0.54	98
5	-	NR

[a] Reaction conditions: nitrobenzene (0.2 mmol), benzyl alcohol (0.6 mmol), petroleum ether (6 mL), Zn/CS (0.09 mol% [Zn], Zn: $PhNO_2$), reacted at 120 °C for 21 h. [b] Yield was measured by GC.

Table S6. Effect of catalyst dosage on borrowing hydrogen reaction of nitrobenzene and benzyl alcohol [a]

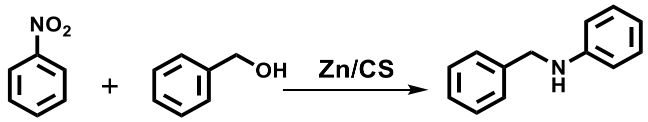

Entry	Catalyst (mol/%)	Yield (%) [b]
1	0.03	28
2	0.05	36
3	0.07	45
4	0.09	95
5	0.12	59
6	-	NR

[a] Reaction conditions: nitrobenzene (0.2 mmol), benzyl alcohol (0.6 mmol), KOH (0.54 mmol), petroleum ether (6 mL), reacted at 120 °C for 24 h. [b] Yield was measured by GC.

Table S7. Effect of molar ratio of the substrate on borrowing hydrogen reaction of nitrobenzene and benzyl alcohol [a]

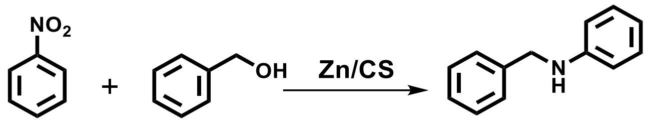

Entry	$PhNO_2:PhCH_2OH$	Yield (%) [b]
1	1:1	5
2	1:1.5	20
3	1:2.0	56
4	1:3.0	98
5	1.5:1.0	Trace

[a] Reaction conditions: KOH (0.54 mmol), petroleum ether (6 mL), Zn/CS (0.09 mol% [Zn], Zn: $PhNO_2$), reacted at 120 °C for 21 h. [b] Yield was measured by GC.

Table S8. Some typical cases of supported catalysts catalyzing the borrowing hydrogen reaction of benzyl alcohol and nitrobenzene.

Catalyst	Temperature (ºC)	Catalyst amount (mol%)	Time (h)	Yield (%)	Number of substrates	TOF (h^{-1})	References
Fe_3O_4@N-C/$Pd_{5\%}$	160	1	36	88	10	2.44	*Catal. Commun.* **2022**, 172, 106529.[5]
Pd/HT	130	2	24	7	6	0.15	*Appl. Catal. A.* **2014**, 470, 1-7.[6]
CoO_x@NC-800	120	10	15	84	9	0.56	*Catalysts* **2019**, 9, 116.[7]
Au/TiO_2-VS	120	0.5	14	99	24	14.14	*Chem. Eur. J.* **2011**, 17, 7172-7177.[8]
Au/Ag-Mo-NR	150	--	24	91	12	--	*Chem. Commun.* **2012**, 48, 9391-9393.[9]
Co-N-C/CNT@AC	160	16.88	38	82	6	0.12	*Green Chem.* **2019**, 21, 2129-2137.[10]
Ag-MCP-1	150	1.678	24	93	7	2.3	*J. Colloid Interface Sci.* **2016**, 472, 202-209.[11]
Ag/Al_2O_3	140	2.2	19	98	16	2.34	*Phys. Chem. Chem. Phys.* **2015**, 17, 15012-15018.[12]
Au/Fe_2O_3	160	0.924	8	87	15	11.77	*Chem. Commun.* **2011**, 47, 6476-6478.[13]
Cu/ Al_2O_3-DH (MeOH)	190	0.756	72	78	7	1.43	*Appl. Surf. Sci.* **2020**, 526, 146708.[14]
Pt/C (MeOH)	140	1	15	92	10	6.13	*J. Catal.* **2019**, 371, 47-56.[15]
Pt/C (MeOH)	130	4.7	20	92	31	0.98	*J. Org. Chem.* **2019**, 84, 15389-15398.[16]
Ni-NC-650	200	--	4	66	6	--	*ChemCatChem* **2021**, 13, 4243-4250.[17]
Ir@YSMCNs (MeOH)	170	0.521	30	97	8	6.21	*Asian J. Org. Chem.* **2019**, 8, 487-491.[18]
Co@MgF_2-f-h	110	2.03	24	85	23	1.74	*Mol. Catal.* **2023**, 545, 113186.[19]
Ru-Pd/TiO_2	160	1.24	3	45	0	12.10	*Catal. Sci. Technol.* **2016**, 6, 5473-5482.[20]
Pd_2/TiO_2 (λ＞300 nm)	25	7.547	4	96	13	3.18	*New J. Chem.* **2015**, 39, 2467-2473.[21]
Pd/Ti-NS (λ≤700 nm)	25	0.32	8	85	7	33.2	*J. Catal.* **2018**, 361, 105-115.[22]
Pd/NiTI-LDH (hv)	20	3.77	24	98	7	1.08	*Dalton Trans.* **2023**, 52, 16935-16942.[23]
Pd@NH_2-UiO-66(Zr) (hv)	--	1.858	24	81	10	1.82	*Appl. Catal. B Environ.* **2022**, 305, 121031.[24]
3%Pt/D-TiO_2/Ti_3C ((λ=400 nm)	--	0.69	12	17	--	2.05	*Appl. Organomet. Chem.* **2021**, 35, e6291.[25]
$Pd_{0.8}$/TiO_2 (λ=400 nm)	--	0.80	20	76	5	4.75	*RSC Adv.* **2015**, 5, 14514-14521.[26]
**This Work**	120	0.09	21	98	56	**51.85**	--

S2. Computational Details

Geometry optimizations, total energy calculations, and reaction pathway searches of the composite were performed using the DMol^3 (Materials Studio 2022) software package[27]. The generalized gradient approximation (GGA) with the Perdew-Burke-Ernzerhof (PBE) functional[28], along with the semi-core pseudopotential (SPP) method and double numerical basis sets plus polarization (DNP)[29] were employed. The self-consistent field (SCF) convergence criterion for electronic energy was set to 1.0×10^{-5} Ha. Geometry optimization convergence thresholds were set to 1.0×10^{-5} Ha for energy, 0.004 Ha Å^{-1} for force, and 0.0l Å for displacement. The binding energy was calculated using the formula: $E_{bind} = E_{complex} - (E_{partA} + E_{partB})$. The transition states were located using the linear synchronous transit (LST) and quadratic synchronous transit (QST) methods[30], combined with conjugated gradient (CG) refinement. The Gibbs free energy (G) was obtained using the equation: $G = E_{total} + E_{ZPE} - TS$, where E_{total} was the ground-state electronic energy, E_{ZPE} was the zero-point energy, T was the temperature, and S was the entropy derived from vibrational frequency analysis. Finally, the Gibbs free energy change (ΔG) for each elementary reaction was defined as: $\Delta G = G_P - G_R$, where G_P was the total energy of all products and G_R was the total energy of all reactants.

The Bader charge analysis, density of states (DOS), and charge density difference calculations were carried out using the Vienna Ab initio Simulation Package (VASP, 6.3.2) [31,32]. The exchange-correlation functional was described by the GGA-PBE approach, and core-valence interactions were treated using the projector augmented-wave (PAW) method[33,34]. A plane-wave energy cutoff of 450 eV was used. To model the isolated molecular clusters, all VASP calculations were performed in a $(30 \times 30 \times 30)$ Å^3 cubic simulation cell to avoid spurious interactions between periodic images. Brillouin-zone sampling was carried out using a Γ-centered Monkhorst-Pack scheme with a $1 \times 1 \times 1$ k-point mesh. Dispersion interactions were included using the DFT-D3 correction method proposed by Grimme et al.[35], ensuring an accurate description of long-range van der Waals interactions.

S3. NMR Data of the Products

S3.1 Scope of Nitroarenes

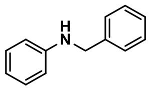

N-benzylaniline (**Pro.1)**: Colorless oil (m=53 mg, 95% isolated yield). ^{1}H NMR (400 MHz, $CDCl_3$) δ 7.39-7.31 (m, 4H), 7.29-7.24 (m, 1H), 7.20-7.15 (m, 2H), 6.74-6.70 (m, 1H), 6.66-6.62 (m, 2H), 4.33 (s, 2H). This spectrum was also similar to the reported (*J. Am. Chem. Soc.* **2018**, 140, 9167-9173; *ACS Catal.* **2018**, 8, 8525 - 8530; *ACS Catal.* **2019**, 9, 9051-9059; *Inorg. Chem.* **2017**, 56, 14682-14687)[36-39].

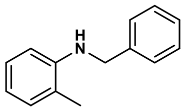

N-benzyl-2-methylaniline (Pro.2): Colorless oil (m=35 mg, 89% isolated yield). ^{1}H NMR (400 MHz, $CDCl_3$) δ 7.40-7.33 (m, 4H), 7.30-7.25 (m, 1H), 7.09 (dd, *J* = 12.7, 7.1 Hz, 2H), 6.67 (t, *J* = 7.3 Hz, 1H), 6.62 (d, *J* = 8.0 Hz, 1H), 4.37 (s, 2H), 3.90 (s, 1H), 2.17 (s, 3H). This spectrum was also similar to the reported (*Green Chem.* **2018**, 20, 2571-2577; *Chem. Eur. J.* **2023**, 29, e202302007)[40,41].

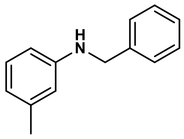

N-benzyl-3-methylaniline (Pro.3): Colorless oil (m=37 mg, 93% isolated yield).^{1}H NMR (400 MHz, $CDCl_3$) δ 7.35-7.29 (m, 4H), 7.28-7.23 (m, 1H), 7.05 (t, *J* = 7.7 Hz, 1H), 6.53 (d, *J* = 7.4 Hz, 1H), 6.46-6.41 (m, 2H), 4.29 (s, 2H), 3.93 (s, 1H), 2.25 (s, 3H). This spectrum was also similar to the reported (*Chem. Eur. J.* **2023**, 29, e202302007; *Eur. J. Inorg. Chem.* **2023**, 26, e202200751)[41,42].

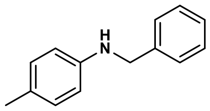

N-benzyl-4-methylaniline (Pro.4): Colorless oil (m=37mg, 94% isolated yield). ^{1}H NMR (400 MHz, $CDCl_3$) δ 7.36-7.29 (m, 4H), 7.26-7.21 (m, 1H), 7.04 (t, *J* = 7.7 Hz,

1H), 6.52 (d, J = 7.4 Hz, 1H), 6.49-6.38 (m, 2H), 4.29 (s, 2H), 4.04 (s, 1H), 2.24 (s, 3H). This spectrum was also similar to the reported (*Chem. Eur. J.* **2023**, 29, e202302007; *Eur. J. Inorg. Chem.* **2023**, 26, e202200751; *J. Org. Chem.* **2023**, 88, 771-787)[41-43].

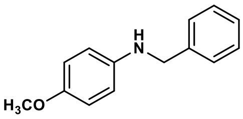

N-benzyl-4-methoxyaniline (**Pro.5**): Yellow oil (m=38 mg, 90% isolated yield).^{1}H NMR (400 MHz, $CDCl_3$) δ 7.40-7.33 (m, 4H), 7.31-7.28 (m, 1H), 6.82-6.76 (m, 2H), 6.66-6.60 (m, 2H), 4.30 (s, 2H), 3.76 (s, 3H). This spectrum was also similar to the reported (*ACS Catal.* **2019**, 9, 9051-9059; *Chem. Eur. J.* **2023**, 29, e202302007; *Eur. J. Inorg. Chem.* **2023**, 26, e202200751)[38,41,42].

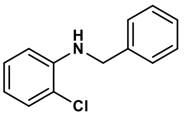

N-benzyl-2-chloroaniline (Pro.6): Colorless oil (m=40 mg, 92% isolated yield). ^{1}H NMR (400 MHz, $CDCl_3$) δ 7.33-7.28 (m, 4H), 7.26-7.21 (m, 2H), 7.06-7.02 (m, 1H), 6.60 (dd, J = 11.3, 4.6 Hz, 2H), 4.72 (s, 1H), 4.35 (s, 2H). This spectrum was also similar to the reported (*Chin. Chem. Lett.* **2022**, 33, 266-270; *Org. Lett.* **2024**, 26, 6065-6069)[44,45].

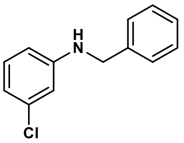

N-benzyl-3-chloroaniline (Pro.7): Colorless oil (m=40 mg, 93% isolated yield). ^{1}H NMR (400 MHz, $CDCl_3$) δ 7.29 (s, 1H), 7.28 (s, 2H), 7.12 (dd, J = 8.5, 7.4 Hz, 1H), 6.99 (t, J = 8.0 Hz, 2H), 6.62-6.59 (m, 1H), 6.55 (t, J = 2.1 Hz, 1H), 6.43 (dd, J = 8.2, 1.6 Hz, 1H), 4.26 (s, 1H), 4.24 (s, 2H). ^{13}C NMR (101 MHz, $CDCl_3$) δ 134.45 (s), 129.63 (s), 128.74 (s), 128.11 (d, J = 9.2 Hz), 126.89 (d, J = 3.7 Hz), 116.92 (s), 111.98 (s), 110.62 (s), 47.58 (s). This spectrum was also similar to the reported (*Chin. Chem. Lett.* **2022**, 33, 266-270; *New J. Chem.* **2022**, 46, 19100-19103)[44,46].

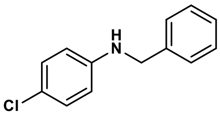

N-benzyl-4-chloroaniline (Pro.8): Colorless oil (m=41 mg, 95% isolated yield). ^{1}H NMR (400 MHz, $CDCl_3$) δ 7.32 (d, *J* = 4.5 Hz, 4H), 7.29-7.24 (m, 1H), 7.12-7.04 (m, 2H), 6.53-6.48 (m, 2H), 4.26 (s, 2H), 4.00 (s, 1H). This spectrum was also similar to the reported (*ACS Catal.* **2018**, 8, 8525-8530; *Inorg. Chem.* **2017**, 56, 14682-14687; *Chin. Chem. Lett.* **2022**, 33, 266-270)[37,39,44].

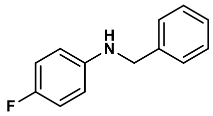

N-benzyl-4-fluoroaniline (Pro.9): Colorless oil (m=31 mg, 77% isolated yield). ^{1}H NMR (400 MHz, $CDCl_3$) δ 7.40-7.31 (m, 5H), 6.88 (t, *J* = 8.7 Hz, 2H), 6.65-6.52 (m, 2H), 4.29 (s, 2H). ^{13}C NMR (101 MHz, $CDCl_3$) δ 137.97 (s), 127.56 (s), 126.33 (d, *J* = 19.5 Hz), 114.68 (s), 114.46 (s), 112.70 (d, *J* = 7.3 Hz), 47.93 (s). This spectrum was also similar to the reported (*Eur. J. Inorg. Chem.* **2023**, 26, e202200751; *J. Org. Chem.* **2023**, 88, 771-787)[42,43].

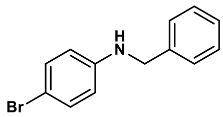

N-benzyl-4-bromoaniline (Pro.10): Colorless oil (m=52 mg, 99% isolated yield). ^{1}H NMR (400 MHz, $CDCl_3$) δ 7.35 (d, *J* = 4.4 Hz, 4H), 7.30 (dd, *J* = 4.8, 3.8 Hz, 1H), 7.27-7.25 (m, 2H), 7.25-7.22 (m, 1H), 6.54-6.50 (m, 2H), 4.31 (s, 2H). This spectrum was also similar to the reported (*Eur. J. Inorg. Chem.* **2023**, 26, e202200751; *Org. Lett.* **2023**, 25, 163-168)[42,47].

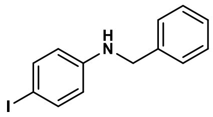

N-benzyl-4-iodoaniline (Pro.11): Colorless oil (m=59 mg, 96% isolated yield). ^{1}H NMR (400 MHz, $CDCl_3$) δ 7.33 (d, *J* = 2.2 Hz, 1H), 7.31-7.27 (m, 2H), 7.26-7.20 (m, 1H), 7.16-7.10 (m, 2H), 6.71-6.66 (m, 1H), 6.58 (dd, *J* = 8.6, 0.9 Hz, 2H), 4.26 (s, 2H), 3.94 (s, 1H). ^{13}C NMR (101 MHz, $CDCl_3$) δ 147.15 (s), 138.46 (s), 128.28 (s), 127.64 (s), 126.52 (s), 126.23 (s), 116.57 (s), 111.88 (s), 47.30 (s). This spectrum was also similar to the reported (*Eur. J. Inorg. Chem.* **2023**, 26, e202200751; *Org. Biomol. Chem.* **2023**, 21, 1038-1045)[42,48].

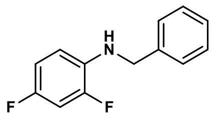

N-benzyl-2,4-difluoroaniline (Pro.12): Colorless oil (m=38 mg, 88% isolated yield).^{1}H NMR (400 MHz, $CDCl_3$) δ 7.97 (dd, *J* = 9.1, 6.0 Hz, 1H), 7.47 (d, *J* = 7.4 Hz, 2H), 7.44-7.39 (m, 2H), 7.35 (dd, *J* = 8.4, 5.8 Hz, 1H), 6.83 (dd, *J* = 10.2, 2.5 Hz, 1H), 6.76-6.71 (m, 1H), 5.23 (s, 2H). ^{13}C NMR (101 MHz, $CDCl_3$) δ 166.08 (s), 163.54 (s), 153.38 (d, *J* = 11.5 Hz), 134.02 (s), 128.03 (s), 127.68 (s), 127.37 (d, *J* =11.5 Hz), 126.17 (s), 106.97 (s), 102.18 (s), 101.91 (s), 70.69 (s). This spectrum was also similar to the reported (*Catal. Sci. Technol.* **2016**, 6, 4554-4564)[49].

S3.2 Scope of Alcohols

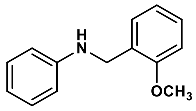

N-(2-methoxybenzyl) aniline (Pro.13): Yellow oil (m=35 mg, 83% isolated yield). ^{1}H NMR (400 MHz, $CDCl_3$) δ 7.32 (d, *J* = 7.4 Hz, 1H), 7.27-7.23 (m, 1H), 7.21-7.13 (m, 2H), 6.91 (dd, *J* = 12.9, 7.8 Hz, 2H), 6.75-6.62 (m, 3H), 4.34 (s, 2H), 3.86 (s, 3H). This spectrum was also similar to the reported (*Chin. Chem. Lett.* **2022**, 33, 266-270; *Tetrahedron* **2024**, 151, 133789)[44,50].

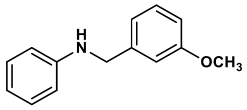

N-(3-methoxybenzyl) aniline (Pro.14): Yellow oil (m=40 mg, 95% isolated yield). ^{1}H NMR (400 MHz, $CDCl_3$) δ 7.36 (q, *J* = 7.9 Hz, 4H), 7.30-7.24 (m, 1H), 7.09 (t, *J* = 8.1 Hz, 1H), 6.33-6.25 (m, 2H), 6.21 (t, *J* = 2.2 Hz, 1H), 4.32 (s, 2H), 4.15 (s, 1H), 3.75 (s, 3H).^{13}C NMR (101 MHz, $CDCl_3$) δ 160.06 (s), 154.40 (d, *J* = 1139.6 Hz), 170.81-129.33 (m), 144.14 (dd, *J* = 2064.4, 1037.7 Hz), 170.81-126.86 (m), 217.50-126.38 (m), 105.26 (s), 101.96 (s), 98.14 (s), 54.29 (s), 47.57 (s). This spectrum was also similar to the reported (*Chin. Chem. Lett.* **2022**, 33, 266-270; *Synlett.* **2023**, 34, 2097-2102)[44,51].

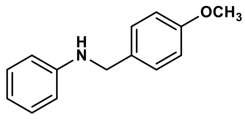

N-(4-methoxybenzyl) aniline (Pro.15): Yellow oil (m=41 mg, 97% isolated yield).^{1}H NMR (400 MHz, $CDCl_3$) δ 7.30-7.23 (m, 2H), 7.19-7.12 (m, 2H), 6.90-6.82 (m, 2H), 6.75-6.66 (m, 1H), 6.65-6.56 (m, 2H), 4.22 (s, 2H), 3.90 (s, 1H), 3.77 (dd, *J* = 2.5, 1.3 Hz, 3H). This spectrum was also similar to the reported (*ACS Catal.* **2019**, 9, 9051-9059; *Chem. Eur. J.* **2023**, 29, e202302007)[38,41].

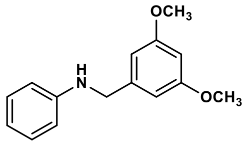

N-(3,5-dimethoxybenzyl) aniline (Pro.16): Colorless oil (m=47 mg, 96% isolated yield). ^{1}H NMR (400 MHz, $CDCl_3$) δ 7.28-7.21 (m, 1H), 7.09-7.02 (m, 1H), 6.75-6.58 (m, 3H), 6.52 (d, *J* = 1.9 Hz, 2H), 6.37 (s, 1H), 4.71 (s, 1H), 4.32 (s, 2H), 3.76 (s, 6H). This spectrum was also similar to the reported (*J. Am. Chem. Soc.* **2020**, 142, 14427-14431; *J. Org. Chem.* **2018**, 83, 8533-8542)[52,53].

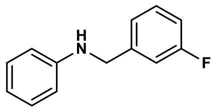

N-(3-fluorobenzyl) aniline (Pro.17): Light gray liquid (m=38 mg, 94% isolated yield). ^{1}H NMR (400 MHz, $CDCl_3$) δ 7.36-7.31 (m, 1H), 7.25-7.16 (m, 3H), 7.12 (d, *J* = 9.7 Hz, 1H), 7.01-6.97 (m, 1H), 6.79 (t, *J* = 7.3 Hz, 1H), 6.68 (d, *J* = 7.7 Hz, 2H), 4.38 (s, 2H).This spectrum was also similar to the reported (*Chem. Eur. J.* **2023**, 29, e202302007; *Chem. Commun.* **2018**, 54, 4302-4305)[41,54].

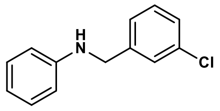

N-(3-chlorobenzyl) aniline (Pro.18): Light gray liquid (m=40 mg, 94% isolated yield).^{1}H NMR (400 MHz, $CDCl_3$) δ 7.38 (s, 1H), 7.26 (d, *J* = 3.5 Hz, 3H), 7.19 (dd, *J* = 8.4, 7.4 Hz, 2H), 6.76 (t, *J* = 7.3 Hz, 1H), 6.64 (d, *J* = 7.7 Hz, 2H), 4.34 (s, 2H). This spectrum was also similar to the reported (*Chem. Eur. J.* **2023**, 29, e202302007; *Chem. Eur. J* **2023**, 29, e202302007)[41,55].

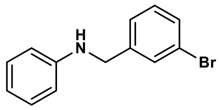

N-(3-bromobenzyl) aniline (Pro.19): Colorless oil (m=45 mg, 87% isolated yield).^{1}H NMR (400 MHz, $CDCl_3$) δ 7.49 (s, 1H), 7.36 (d, *J* = 7.8 Hz, 1H), 7.26 (d, *J* = 7.7 Hz, 1H), 7.15 (dd, *J* = 15.4, 7.5 Hz, 3H), 6.71 (t, *J* = 7.3 Hz, 1H), 6.60 (d, *J* = 7.8 Hz, 2H), 4.53 (s, 1H), 4.28 (s, 2H). This spectrum was also similar to the reported (*Inorg. Chem.* **2018**, 57, 14582-14593; *Eur. J. Org. Chem.* **2022**, 2022, e202200982)[56,57].

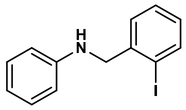

N-(2-iodobenzyl) aniline (Pro.20): Colorless oil (m=57 mg, 93% isolated yield). ^{1}H NMR (400 MHz, $CDCl_3$) δ 7.36 (dd, *J* = 3.3, 2.7 Hz, 3H), 7.29-7.26 (m, 1H), 7.20-7.15 (m, 2H), 6.72 (t, *J* = 7.3 Hz, 1H), 6.67-6.62 (m, 2H), 4.33 (s, 2H), 4.17 (s, 1H).^{13}C NMR (101 MHz, $CDCl_3$) δ 147.19 (s), 138.52 (s), 128.54 (d, *J* = 7.0 Hz), 127.87 (s), 126.79 (s), 126.49 (s), 117.00 (d, *J* = 8.1 Hz), 112.22 (s), 47.66 (s). This spectrum was also similar to the reported (*Org. Lett.* **2017**, 19, 1578-1581; *Tetrahedron Lett.* **2015**, 56, 6448-6451)[58,59].

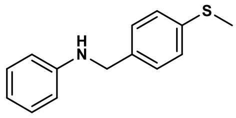

N-(4-(methylthio) benzyl) aniline (Pro.21): Yellow liquid (m=44 mg, 96% isolated yield). ^{1}H NMR (400 MHz, $CDCl_3$) δ 7.23 (dd, *J* = 22.8, 8.3 Hz, 4H), 7.14 (t, *J* = 7.8 Hz, 2H), 6.69 (t, *J* = 7.3 Hz, 1H), 6.59 (d, *J* = 8.0 Hz, 2H), 4.25 (s, 2H), 3.99 (s, 1H), 2.44 (s, 3H). This spectrum was also similar to the reported (*Chem. Eur. J.* **2023**, 29, e202302007; *Angew. Chem. Int. Ed.* **2020**, 59, 11789-11793; *Org. Biomol. Chem.* **2021**, 19, 3451-3461)[41,60,61].

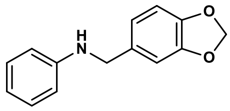

N-(benzo[d] [1,3] dioxol-5-ylmethyl) aniline (Pro.22): Yellow liquid (m=18 mg, 24% isolated yield). ^{1}H NMR (400 MHz, $CDCl_3$) δ 7.26-7.18 (m, 2H), 6.92-6.84 (m, 2H), 6.80 (t, *J* = 8.1 Hz, 2H), 6.73 (d, *J* = 7.9 Hz, 2H), 5.97 (s, 2H), 4.27 (s, 2H).This

spectrum was also similar to the reported (*Chem. Eur. J.* **2023**, 29, e202302007; *J. Org. Chem.* **2023**, 88, 771-787; *J. Org. Chem.* **2023**, 88, 5944-5961)[41,43,62].

S3.3 Scope of Nitroarenes and Alcohols

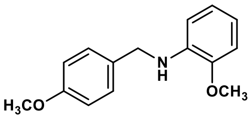

2-methoxy-N-(4-methoxybenzyl) aniline (Pro.28): Colorless oil (m=45 mg, 93% isolated yield). ^{1}H NMR (400 MHz, $CDCl_3$) δ 7.33-7.22 (m, 2H), 6.88-6.83 (m, 2H), 6.81 (dd, *J* = 7.6, 1.3 Hz, 1H), 6.75 (dd, *J* = 7.9, 1.1 Hz, 1H), 6.67-6.63 (m, 1H), 6.59 (dd, *J* = 7.8, 1.3 Hz, 1H), 4.53 (s, 1H), 4.24 (s, 2H), 3.77 (d, *J* = 14.9 Hz, 6H). This spectrum was also similar to the reported (*Tetrahedron* **2014**, 70, 8817-8821; *Org. Lett.* **2015**, 17, 173-175)[63,64].

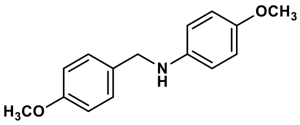

4-methoxy-N-(4-methoxybenzyl) aniline (Pro.29): Colorless oil (m=43 mg, 89% isolated yield). ^{1}H NMR (400 MHz, $CDCl_3$) δ 7.27-7.22 (m, 2H), 6.86-6.81 (m, 2H), 6.77-6.71 (m, 2H), 6.59-6.54 (m, 2H), 4.16 (s, 2H), 3.73 (d, J = 22.8 Hz, 6H), 3.43 (s, 1H). This spectrum was also similar to the reported (*Chem. Eur. J.* **2022**, 17, e202200013; *Adv. Synth. Catal.* **2024**, 366, 2035-2043)[65,66].

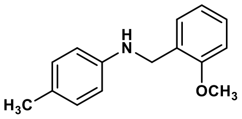

N-(2-methoxybenzyl)-4-methylaniline (Pro.30): Colorless oil (m=41 mg, 91% isolated yield). ^{1}H NMR (400 MHz, $CDCl_3$) δ 7.28 (d, *J* = 7.1 Hz, 1H), 7.25-7.19 (m, 1H), 6.96 (d, *J* = 8.2 Hz, 2H), 6.89-6.84 (m, 2H), 6.57 (d, *J* = 8.4 Hz, 2H), 4.29 (s, 2H), 3.83 (s, 3H), 3.41 (s, 1H), 2.21 (s, 3H). ^{13}C NMR (101 MHz, $CDCl_3$) δ 156.31 (s), 145.00 (s), 128.59 (s), 127.85 (s), 127.16 (s), 126.41 (s), 125.54 (s), 119.44 (s), 112.26 (s), 109.13 (s), 54.21 (s), 42.81 (s), 19.33 (s). This spectrum was also similar to the reported (*J. Organomet. Chem.* **1990**, 391, 179-188)[67].

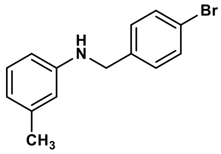

N-(4-bromobenzyl)-3-methylaniline (Pro.31): Colorless oil (m=54 mg, 98% isolated yield). ^{1}H NMR (400 MHz, $CDCl_3$) δ 7.52 (s, 1H), 7.38 (d, *J* = 7.9 Hz, 1H), 7.29 (d, *J* = 7.6 Hz, 1H), 7.19 (t, *J* = 7.8 Hz, 1H), 6.98 (d, *J* = 8.2 Hz, 2H), 6.54 (d, *J* = 8.4 Hz, 2H), 4.29 (s, 2H), 2.23 (s, 3H). This spectrum was also similar to the reported (*Bull. Korean Chem. Soc.* **2015**, 36, 2557-2560)[68].

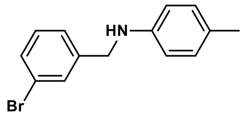

N-(3-bromobenzyl)-4-methylaniline (Pro.32): Colorless oil (m=42 mg, 77% isolated yield). ^{1}H NMR (400 MHz, $CDCl_3$) δ 7.52 (s, 1H), 7.38 (d, *J* = 7.9 Hz, 1H), 7.29 (d, *J* = 7.6 Hz, 1H), 7.19 (t, *J* = 7.8 Hz, 1H), 6.98 (d, *J* = 8.2 Hz, 2H), 6.54 (d, *J* = 8.4 Hz, 2H), 4.29 (s, 2H), 2.23 (s, 3H). ^{13}C NMR (101 MHz, $CDCl_3$) δ 145.51 (s), 142.26 (s), 130.49-130.02 (m), 129.82 (d, *J* = 4.2 Hz), 128.64 (s), 127.59 (s), 127.15 (s), 125.93 (s), 122.77 (s), 113.14 (d, *J* = 6.7 Hz), 77.40 (s), 77.08 (s), 76.76 (s), 48.11 (s), 20.44 (s). HRMS (ESI) calcd. for $C_{14}H_{15}BrN$ $[M+H]^+$ 276.0382, found 276.0381. This spectrum was also similar to the reported (*Adv. Synth. Catal.* **2018**, 360, 556-561)[69].

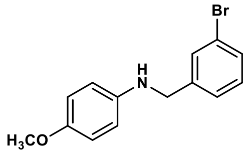

N-(3-bromobenzyl)-4-methoxyaniline (Pro.33): Colorless oil (m=55 mg, 95% isolated yield). ^{1}H NMR (400 MHz, $CDCl_3$) δ 7.40-7.33 (m, 4H), 7.31-7.08 (m, 1H), 6.82-6.77 (m, 2H), 6.65-6.61 (m, 2H), 4.30 (s, 2H), 3.76 (s, 3H). ^{13}C NMR (101 MHz, $CDCl_3$) δ 152.27 (s), 142.39 (s), 139.65 (s), 128.63 (s), 127.60 (s), 127.22 (s), 114.94 (s), 114.21 (s), 55.84 (s), 49.32 (s). This spectrum was also similar to the reported (*Chem. Eur. J.* **2013**, 19, 4021-4029)[70].

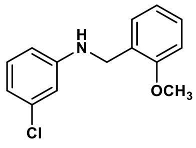

3-chloro-N-(2-methoxybenzyl) aniline (Pro.34): Colorless oil (m=37 mg, 75% isolated yield). ^{1}H NMR (400 MHz, $CDCl_3$) δ 7.27 (d, *J* = 7.7 Hz, 2H), 7.05 (t, *J* = 8.2 Hz, 1H), 6.91 (dd, *J* = 13.1, 7.6 Hz, 2H), 6.69-6.63 (m, 2H), 6.54-6.50 (m, 1H), 4.31 (s, 2H), 3.86 (s, 3H). ^{13}C NMR (101 MHz, $CDCl_3$) δ 158.32 (s), 150.41 (s), 135.87 (s), 131.05 (s), 129.84 (s), 129.50 (s), 127.60 (s), 121.51 (s), 118.12 (s), 113.64 (s), 112.29 (s), 111.27 (s), 56.26 (s), 44.29 (s). HRMS (ESI) calcd. for $C_{14}H_{14}ClONNa$ $[M+Na]^+$ 270.0656, found 270.0658.

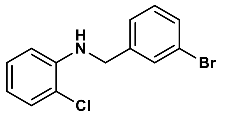

N-(3-bromobenzyl)-2-chloroaniline (Pro.35): Colorless oil (m=53 mg, 91% isolated yield). ^{1}H NMR (400 MHz, $CDCl_3$) δ 7.49 (s, 1H), 7.41-7.33 (m, 1H), 7.26 (dd, *J* = 7.9, 1.4 Hz, 2H), 7.22-7.15 (m, 1H), 7.10-7.02 (m, 1H), 6.65-6.60 (m, 1H), 6.54 (dd, *J* = 8.2, 1.3 Hz, 1H), 4.76 (s, 1H), 4.35 (s, 2H). ^{13}C NMR (101 MHz, $CDCl_3$) δ 143.25 (s), 141.06 (s), 130.32-129.80 (m), 128.95 (s), 127.60 (s), 125.40 (s), 122.61 (s), 118.97 (s), 117.54 (s), 111.28 (s), 47.02 (s). HRMS (ESI) calcd. for $C_{13}H_{12}ClBrN$ $[M+H]^+$ 295.9836, found 295.9835.

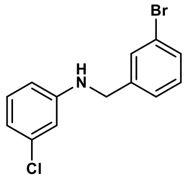

N-(3-bromobenzyl)-3-chloroaniline (Pro.36): Colorless oil (m=58 mg, 99% isolated yield). ^{1}H NMR (400 MHz, $CDCl_3$) δ 7.44-7.39 (m, 1H), 7.31 (dd, *J* = 23.5, 5.1 Hz, 1H), 7.22-7.18 (m, 1H), 7.14 (t, *J* = 7.7 Hz, 1H), 7.00 (t, *J* = 8.0 Hz, 1H), 6.62 (dd, *J* = 7.9, 1.1 Hz, 1H), 6.52 (t, *J* = 2.0 Hz, 1H), 6.40 (dd, *J* = 8.2, 2.2 Hz, 1H), 4.22 (s, 2H). ^{13}C NMR (101 MHz, $CDCl_3$) δ 150.03 (s), 142.46 (s), 136.26 (s), 131.72 (s), 131.49 (d, *J* = 1.7 Hz), 127.03 (s), 124.05 (s), 118.96 (s), 113.78 (s), 112.34 (s), 48.67 (s). HRMS (ESI) calcd. for $C_{13}H_{12}ClBrN$ $[M+H]^+$ 295.9836, found 295.9838.

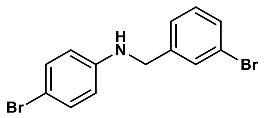

4-bromo-N-(3-bromobenzyl) aniline (Pro.37): Colorless oil (m=59 mg, 87%

isolated yield). ^{1}H NMR (400 MHz, $CDCl_3$) δ 7.49 (s, 1H), 7.41-7.38 (m, 1H), 7.25 (dd, *J* = 3.4, 1.9 Hz, 2H), 7.23-7.17 (m, 2H), 6.49-6.44 (m, 2H), 4.27 (s, 2H), 4.16 (s, 1H). ^{13}C NMR (101 MHz, $CDCl_3$) δ 145.82 (s), 140.57 (s), 131.22 (s), 129.78-129.28 (m), 125.01 (s), 122.05 (s), 113.72 (s), 108.73 (s), 46.85 (s). HRMS (ESI) calcd. for $C_{13}H_{12}Br_2N$ $[M+H]^+$ 339.9331, found 339.9326.

S3.4 Substrates of Heterocycles

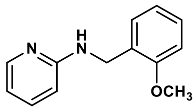

N-(2-methoxybenzyl) pyridin-2-amine (Pro.38): Colorless oil (m=42 mg, 98% isolated yield). ^{1}H NMR (400 MHz, $CDCl_3$) δ 8.23-8.15 (m, 1H), 7.61-7.54 (m, 1H), 7.46 (d, *J* = 7.4 Hz, 1H), 7.32-7.24 (m, 1H), 6.96 (t, *J* = 7.4 Hz, 1H), 6.90 (d, *J* = 8.2 Hz, 1H), 6.86 (dd, *J* = 6.5, 5.6 Hz, 1H), 6.82 (d, *J* = 8.4 Hz, 1H), 5.43 (s, 2H), 3.84 (s, 3H). ^{13}C NMR (101 MHz, $CDCl_3$) δ 163.09 (s), 156.57 (s), 146.13 (s), 137.73 (s), 128.40 (s), 128.18 (s), 124.87 (s), 119.63 (s), 115.93 (s), 110.51 (s), 109.57 (s), 62.15 (s), 54.62 (s). This spectrum was also similar to the reported (*Org. Lett.* **2011**, 13, 6184-6187; *Tetrahedron* **2016**, 72, 264-272)[71,72].

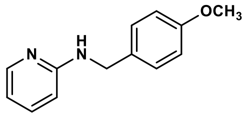

N-(4-methoxybenzyl) pyridin-2-amine (Pro.39): Colorless oil (m=42 mg, 99% isolated yield). ^{1}H NMR (400 MHz, $CDCl_3$) δ 8.18 (dd, *J* = 5.0, 1.6 Hz, 1H), 7.61-7.54 (m, 1H), 7.43-7.37 (m, 2H), 6.94-6.89 (m, 2H), 6.89-6.85 (m, 1H), 6.78 (d, *J* = 8.4 Hz, 1H), 5.30 (s, 2H), 3.81 (s, 3H). This spectrum was also similar to the reported (*Chem. Eur. J.* **2023**, 29, e202302007; *J. Org. Chem.* **2023**, 88, 771-787; *J. Org. Chem.* **2021**, 86, 2254-2263)[41,43,73].

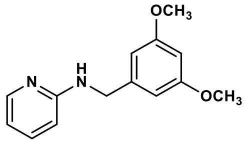

N-(3,5-dimethoxybenzyl) pyridin-2-amine (Pro.40): Colorless oil (m=44 mg, 91% isolated yield). ^{1}H NMR (400 MHz, $CDCl_3$) δ 8.16 (dd, *J* = 4.9, 1.3 Hz, 1H), 7.60-7.54 (m, 1H), 6.87 (dd, *J* = 6.6, 5.5 Hz, 1H), 6.80 (d, *J* = 8.3 Hz, 1H), 6.60 (d, *J* = 2.1 Hz, 2H), 6.40 (t, *J* = 2.2 Hz, 1H), 5.31 (s, 2H), 3.78 (s, 6H). ^{13}C NMR (101 MHz, $CDCl_3$) δ 162.43 (s), 159.79 (s), 145.74 (s), 138.58 (s), 137.55 (s), 115.87 (s), 110.23 (s), 104.57 (s), 98.70 (s), 66.33 (s), 54.25 (s).

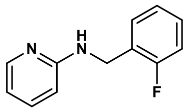

N-(2-fluorobenzyl) pyridin-2-amine (Pro.41): Colorless oil (m=25 mg, 62% isolated yield). ^{1}H NMR (400 MHz, $CDCl_3$) δ 8.18 (dd, *J* = 4.9, 1.3 Hz, 1H), 7.62-7.55 (m, 1H), 7.51 (t, *J* = 7.4 Hz, 1H), 7.34-7.27 (m, 1H), 7.16-7.06 (m, 2H), 6.89 (dd, *J* = 6.5, 5.5 Hz, 1H), 6.81 (d, *J* = 8.4 Hz, 1H), 5.45 (s, 2H), 5.45 (s, 2H).

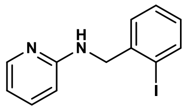

N-(2-iodobenzyl) pyridin-2-amine (Pro.42): Colorless oil (m=49 mg, 79% isolated yield). ^{1}H NMR (400 MHz, $CDCl_3$) δ 8.25-8.03 (m, 1H), 7.95-7.89 (m, 1H), 7.70-7.48 (m, 2H), 7.48-7.35 (m, 2H), 7.22-7.02 (m, 1H), 6.98-6.84 (m, 1H), 5.42 (d, *J* = 5.4 Hz, 2H), 1.63 (s, 1H). This spectrum was also similar to the reported (*J. Org. Chem.* **2010**, 75, 2206-2218)[74].

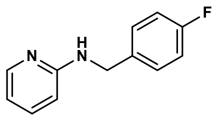

N-(4-fluorobenzyl) pyridin-2-amine (Pro.43): Colorless oil (m=31 mg, 77% isolated yield). ^{1}H NMR (400 MHz, $CDCl_3$) δ 8.18 (dd, *J* = 4.9, 1.3 Hz, 1H), 7.65-7.55 (m, 1H), 7.45 (dd, *J* = 8.5, 5.5 Hz, 2H), 7.06 (t, *J* = 8.7 Hz, 2H), 6.90 (dd, *J* = 6.5, 5.4 Hz, 1H), 6.81 (d, *J* = 8.3 Hz, 1H), 5.36 (s, 2H). This spectrum was also similar to the reported (*J. Org. Chem.* **2023**, 88, 771-787; *J. Org. Chem.* **2023**, 88, 5944-5961; *Eur. J. Inorg. Chem.* **2023**, 26, e202300263)[43,62,75].

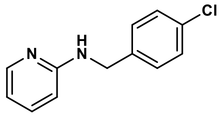

N-(4-chlorobenzyl) pyridin-2-amine (Pro.44): Colorless oil (m=36 mg, 83%

isolated yield). ^{1}H NMR (400 MHz, $CDCl_3$) δ 8.20 (dd, *J* = 4.9, 1.4 Hz, 1H), 7.67-7.58 (m, 1H), 7.43 (d, *J* = 8.4 Hz, 2H), 7.40-7.34 (m, 2H), 6.97-6.90 (m, 1H), 6.84 (d, *J* = 8.3 Hz, 1H), 5.39 (s, 2H). This spectrum was also similar to the reported (*J. Org. Chem.* **2023**, 88, 5944-5961; *Eur. J. Inorg. Chem.* **2023**, 26, e202300263)[62,75].

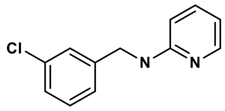

2-((3-chlorobenzyl)-λ²-azaneyl) pyridine (Pro.45): Colorless oil (m=30 mg, 69% isolated yield). ^{1}H NMR (400 MHz, $CDCl_3$) δ 8.74 (d, *J* = 2.9 Hz, 1H), 8.08 (d, *J* = 8.6 Hz, 1H), 7.43 (s, 1H), 7.39 (dd, *J* = 8.2, 4.2 Hz, 1H), 7.32 (d, *J* = 7.6 Hz, 1H), 7.26 (d, *J* = 3.3 Hz, 1H), 7.08 (d, *J* = 8.1 Hz, 1H), 6.65 (s, 1H), 6.59 (d, *J* = 7.6 Hz, 1H), 4.55 (s, 2H). This spectrum was also similar to the reported (*J. Org. Chem.* **2023**, 88, 771-787; *J. Org. Chem.* **2023**, 88, 5944-5961; *Eur. J. Inorg. Chem.* **2023**, 26, e202300263)[43,62,75].

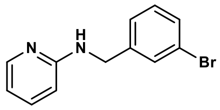

N-(3-bromobenzyl) pyridin-2-amine (Pro.46): Colorless oil (m=51 mg, 97% isolated yield). ^{1}H NMR (400 MHz, $CDCl_3$) δ 8.18 (dd, *J* = 5.0, 1.4 Hz, 1H), 7.66-7.57 (m, 2H), 7.45 (d, *J* = 7.8 Hz, 1H), 7.38 (d, *J* = 7.7 Hz, 1H), 7.24 (t, *J* = 7.8 Hz, 1H), 6.92-6.89 (m, 1H), 6.83 (d, *J* = 8.4 Hz, 1H), 5.37 (s, 2H), 1.80 (s, 1H). ^{13}C NMR (101 MHz, $CDCl_3$) δ 162.97 (s), 146.52 (s), 139.48 (s), 138.50 (s), 130.51 (d, *J* = 4.2 Hz), 129.71 (s), 126.03 (s), 122.25 (s), 116.87 (s), 111.03 (s), 66.22 (s). This spectrum was also similar to the reported (*J. Org. Chem.* **2023**, 88, 771-787; *Eur. J. Inorg. Chem.* **2023**, 26, e202300263)[43,75].

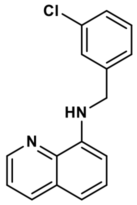

N-(3-chlorobenzyl) quinolin-8-amine (Pro.47): Yellow liquid (m=34 mg, 63% isolated yield). ^{1}H NMR (400 MHz, $CDCl_3$) δ 8.73 (d, *J* = 2.8 Hz, 1H), 8.20-8.04 (m, 1H), 7.41 (t, *J* = 6.0 Hz, 2H), 7.32 (dd, *J* = 15.2, 7.3 Hz, 2H), 7.26 (d, *J* = 7.8 Hz, 1H),

7.23 (s, 1H), 7.08 (d, J = 8.1 Hz, 1H), 6.78 (s, 1H), 6.59 (d, J = 7.5 Hz, 1H), 4.54 (s, 2H). This spectrum was also similar to the reported (*Dalton Trans.* **2024**, 53, 5064-5072)[76].

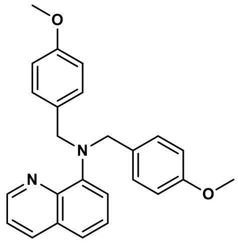

N, N-bis(4-methoxybenzyl) quinolin-8-amine (Pro.48): Colorless oil (m=58 mg, 76% isolated yield).^{1}H NMR (400 MHz, $CDCl_3$) δ 8.73 (dd, J = 4.3, 1.6 Hz, 1H), 8.13 (d, J = 7.4 Hz, 1H), 7.45-7.33 (m, 4H), 7.26-7.22 (m, 2H), 7.16-7.03 (m, 1H), 6.92-6.85 (m, 4H), 6.69 (d, J = 7.7 Hz, 1H), 4.69 (s, 2H), 4.51 (s, 2H), 3.81 (d, J = 1.3 Hz, 6H).

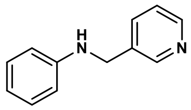

N-(pyridin-3-ylmethyl) aniline (Pro.49): Colorless oil (m=35 mg, 95% isolated yield). ^{1}H NMR (400 MHz, $CDCl_3$) δ 8.63 (s, 1H), 8.52 (d, J = 3.9 Hz, 1H), 7.70 (d, J = 7.8 Hz, 1H), 7.26 (dd, J = 8.1, 4.4 Hz, 1H), 7.21-7.15 (m, 2H), 6.74 (t, J = 7.3 Hz, 1H), 6.63 (d, J = 7.7 Hz, 2H), 4.36 (s, 2H), 4.05 (s, 1H). This spectrum was also similar to the reported (*Eur. J. Org. Chem.* **2015**, 2015, 2972-2977; *J. Org. Chem.* **2022**, 87, 628-643)[77,78].

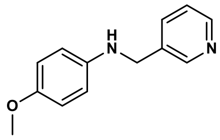

4-methoxy-N-(pyridin-3-ylmethyl) aniline (Pro.50): Yellow liquid (m=42 mg, 81% isolated yield). ^{1}H NMR (400 MHz, $CDCl_3$) δ 8.64 (s, 1H), 8.53 (d, J = 4.2 Hz, 1H), 7.73 (d, J = 7.8 Hz, 1H), 7.29 (dd, J = 7.7, 5.0 Hz, 1H), 6.81-6.75 (m, 2H), 6.63-6.57 (m, 2H), 4.33 (s, 2H), 3.74 (s, 3H). This spectrum was also similar to the reported (*Tetrahedron Lett.* **2011**, 52, 5004-5007; *Can. J. Chem.* **2005**, 83, 909-916)[79,80].

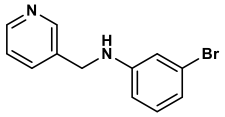

3-bromo-N-(pyridin-3-ylmethyl) aniline (Pro.51): Yellow solid (m=42 mg, 81%

isolated yield). ^{1}H NMR (400 MHz, $CDCl_3$) δ 8.69-8.49 (m, 2H), 7.69 (dd, *J* = 13.1, 7.9 Hz, 1H), 7.31-7.25 (m, 1H), 7.18 (t, *J* = 7.9 Hz, 1H), 7.04-6.82 (m, 1H), 6.75 (dd, *J* = 12.5, 4.7 Hz, 1H), 6.66-6.50 (m, 1H), 4.35 (d, *J* = 11.3 Hz, 2H), 3.31 (s, 1H).

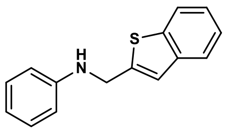

N-(benzo*[b]*thiophen-2-ylmethyl) aniline (Pro.52): Yellow solid (m=40 mg, 84% isolated yield). ^{1}H NMR (400 MHz, $CDCl_3$) δ 7.80 (d, *J* = 7.7 Hz, 1H), 7.71 (d, *J* = 7.7 Hz, 1H), 7.37-7.26 (m, 2H), 7.28-7.16 (m, 3H), 6.79 (t, *J* = 7.3 Hz, 1H), 6.73 (d, *J* = 8.0 Hz, 2H), 4.62 (s, 2H), 4.19 (s, 1H).

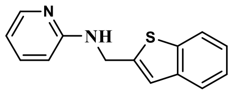

N-(benzo*[b]*thiophen-2-ylmethyl) pyridin-2-amine (Pro.53): Colorless oil (m=41 mg, 87% isolated yield). ^{1}H NMR (400 MHz, $CDCl_3$) δ 8.20 (dd, *J* = 5.0, 1.4 Hz, 1H), 7.82-7.72 (m, 2H), 7.61-7.56 (m, 1H), 7.37-7.27 (m, 3H), 6.90 (dd, *J* = 6.6, 5.5 Hz, 1H), 6.81 (d, *J* = 8.3 Hz, 1H), 5.64 (s, 2H).^{13}C NMR (101 MHz, $CDCl_3$) δ 162.69 (s), 146.43 (s), 140.34 (s), 140.11 (s), 139.07 (s), 138.52 (s), 124.01 (d, *J* = 10.9 Hz), 123.25 (d, *J* = 10.2 Hz), 122.06 (s), 116.99 (s), 111.11 (s), 62.48 (s).

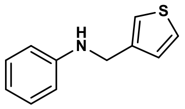

N-(thiophen-3-ylmethyl) aniline (Pro.54): Yellow liquid (m=31 mg, 82% isolated yield). ^{1}H NMR (400 MHz, $CDCl_3$) δ 7.32 (dd, *J* = 4.9, 3.0 Hz, 1H), 7.25-7.16 (m, 3H), 7.10 (dd, *J* = 4.9, 1.1 Hz, 1H), 6.79-6.71 (m, 1H), 6.68 (dd, *J* = 8.5, 0.9 Hz, 2H), 4.35 (s, 2H), 4.10 (s, 1H). This spectrum was also similar to the reported (*Chin. Chem. Lett.* **2022**, 33, 266-270; *Chem. Commun.* **2019**, 55, 6213-6216; *Mol. Catal.* **2021**, 503, 111415)[44,81,82].

S3.5 Scope of Drugs

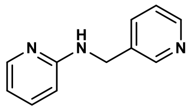

N-(pyridin-2-ylmethyl) pyridin-2-amine (Pro.55): Colorless oil (m=36 mg, 97% isolated yield). ^{1}H NMR (400 MHz, $CDCl_3$) δ 8.70 (d, *J* = 1.7 Hz, 1H), 8.53 (dd, *J* = 4.8, 1.4 Hz, 1H), 8.19-8.10 (m, 1H), 7.76 (dd, *J* = 7.8, 1.7 Hz, 1H), 7.59-7.53 (m, 1H), 7.28-7.24 (m, 1H), 6.90-6.84 (m, 1H), 6.77 (d, *J* = 8.3 Hz, 1H), 5.39 (s, 2H). This spectrum was also similar to the reported (*J. Org. Chem.* **2023**, 88, 771-787; *J. Org. Chem.* **2021**, 86, 2254-2263)[43,73].

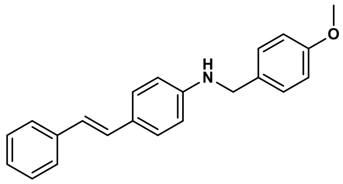

(E)-N-(4-methoxybenzyl)-4-styrylaniline (Pro.56): White soild (m=18 mg, 28% isolated yield). ^{1}H NMR (400 MHz, $CDCl_3$) δ 7.46 (d, *J* = 7.5 Hz, 2H), 7.39-7.27 (m, 7H), 7.19 (t, *J* = 7.3 Hz, 1H), 7.01 (d, *J* = 6.4 Hz, 1H), 6.89 (d, *J* = 8.7 Hz, 2H), 6.62 (d, *J* = 8.5 Hz, 2H), 4.28 (s, 2H), 3.80 (s, 3H).

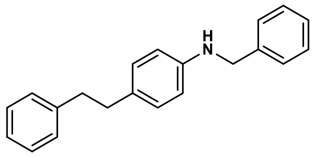

N-benzyl-4-phenethylaniline (Pro.57): Yellow liquid (m=32 mg, 56% isolated yield). ^{1}H NMR (400 MHz, $CDCl_3$) δ 7.40-7.31 (m, 4H), 7.28 (dd, *J* = 10.6, 4.1 Hz, 3H), 7.18 (dd, *J* = 6.9, 3.9 Hz, 3H), 7.00 (d, *J* = 8.3 Hz, 2H), 6.61 (d, *J* = 8.4 Hz, 2H), 4.31 (s, 2H), 2.90-2.78 (m, 4H).

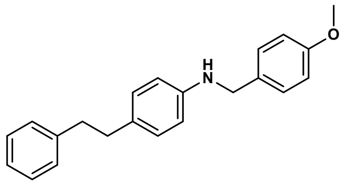

N-(4-methoxybenzyl)-4-phenethylaniline (Pro.58): Yellow soild (m=45 mg, 71% isolated yield). ^{1}H NMR (400 MHz, $CDCl_3$) δ 7.26 (dd, *J* = 16.2, 8.1 Hz, 4H), 7.21-7.15 (m, 3H), 7.00 (d, *J* = 8.2 Hz, 2H), 6.87 (d, *J* = 8.5 Hz, 2H), 6.59 (d, *J* = 8.3 Hz, 2H), 4.23 (s, 2H), 3.79 (s, 3H), 2.89-2.78 (m, 4H).

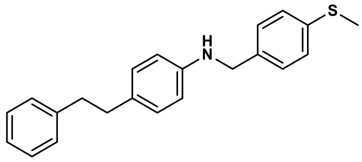

N-(4-(methylthio) benzyl)-4-phenethylaniline (Pro.59): Yellow soild (m=29 mg, 43% isolated yield). ^{1}H NMR (400 MHz, $CDCl_3$) δ 7.28 (t, *J* = 6.5 Hz, 4H), 7.24-7.20 (m, 2H), 7.20-7.15 (m, 3H), 7.03-6.96 (m, 2H), 6.61 (d, *J* = 8.3 Hz, 2H), 4.26 (s, 2H), 2.89-2.77 (m, 4H), 2.46 (s, 3H).

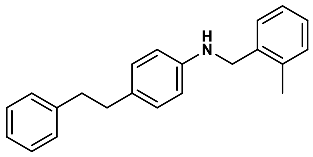

N-(2-methylbenzyl)-4-phenethylaniline (Pro.60): Yellow soild (m=41 mg, 68% isolated yield). ^{1}H NMR (400 MHz, $CDCl_3$) δ 7.34 (d, *J* = 6.6 Hz, 1H), 7.28 (t, *J* = 6.1 Hz, 2H), 7.23-7.14 (m, 6H), 7.02 (d, *J* = 8.3 Hz, 2H), 6.60 (d, *J* = 8.3 Hz, 2H), 4.26 (s, 2H), 2.91-2.78 (m, 4H), 2.36 (s, 3H).

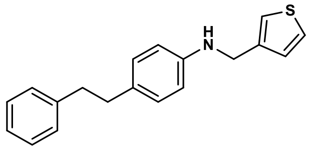

4-phenethyl-N-(thiophen-3-ylmethyl) aniline (Pro.61): Yellow soild (m=36 mg, 61% isolated yield). ^{1}H NMR (400 MHz, $CDCl_3$) δ 7.24-7.19 (m, 3H), 7.12 (dd, *J* = 9.7, 4.4 Hz, 4H), 7.02 (d, *J* = 4.9 Hz, 1H), 6.94 (d, *J* = 8.3 Hz, 2H), 6.58 (d, *J* = 8.3 Hz, 2H), 4.25 (s, 2H), 2.84-2.72 (m, 4H).

S4. NMR Image of the Products

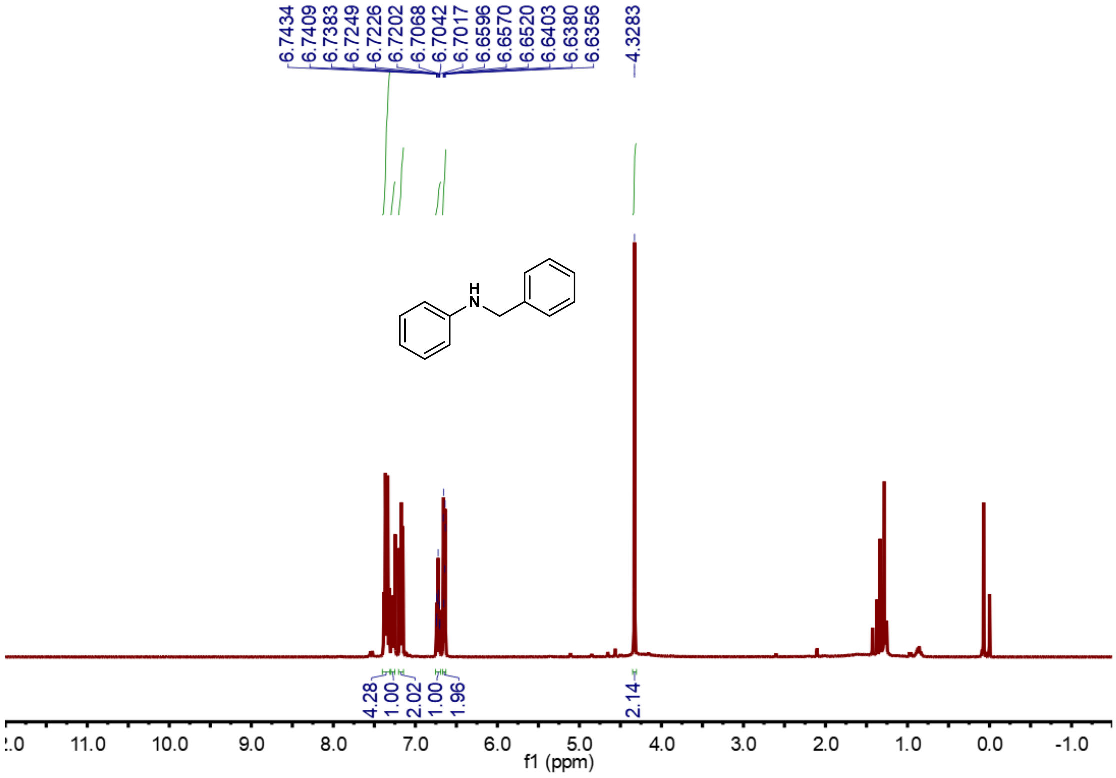

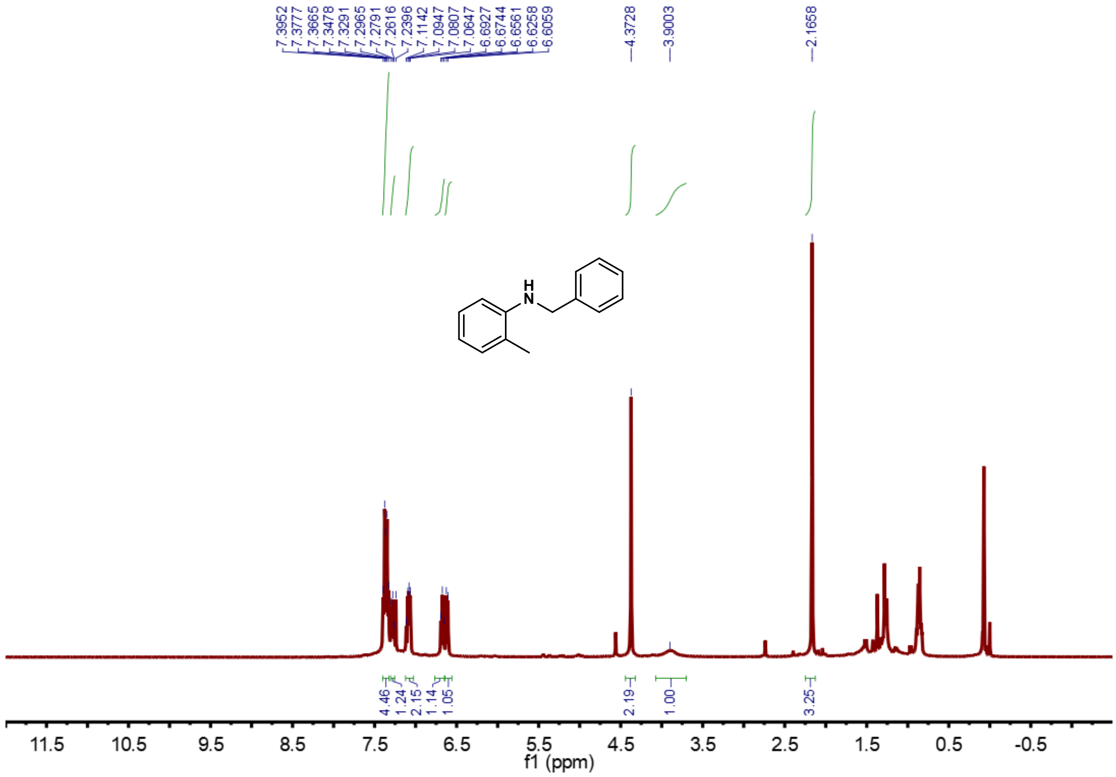

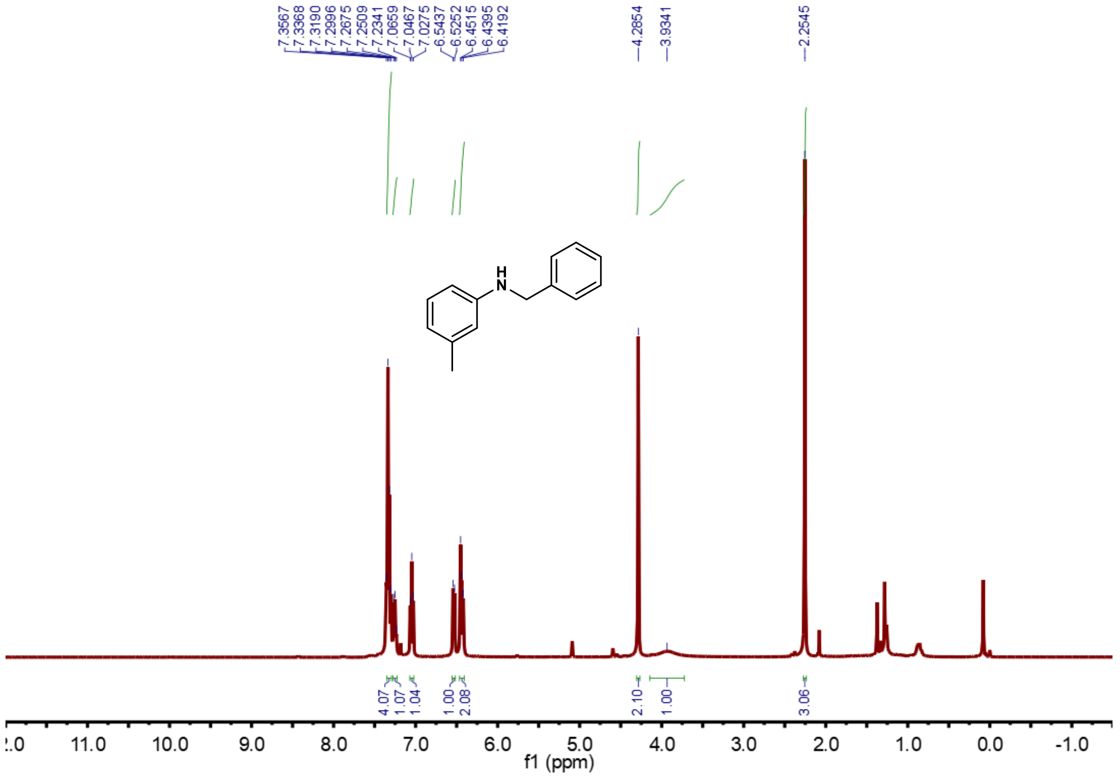
7.3567
7.3368
7.3190
7.2996
7.2675
7.2509
7.2341
7.0659
7.0467
7.0275
6.5437
6.5252
6.4515
6.4395
6.4192
4.2854
3.9341
2.2545
H
N
4.07
1.07
1.04
1.00
2.08
2.10
1.00
3.06
11.0
10.0
9.0
8.0
7.0
6.0
5.0
4.0
3.0
2.0
1.0
0.0
-1.0
f1 (ppm)

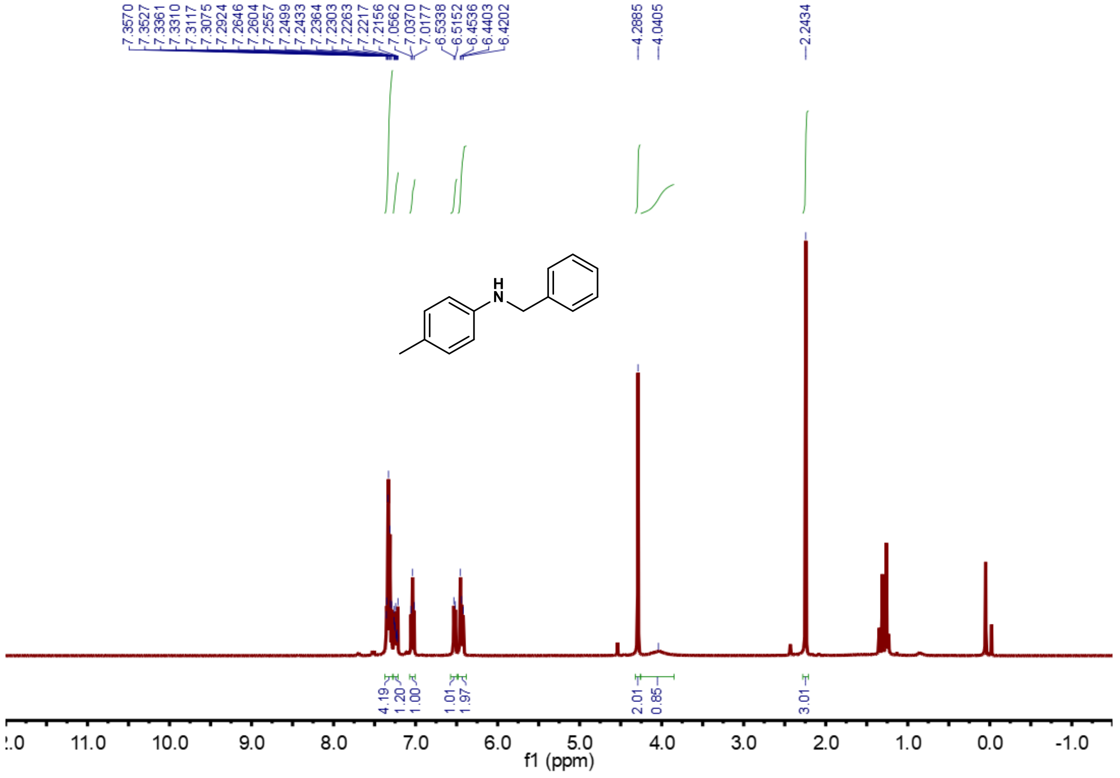
7.3570
7.3527
7.3361
7.3310
7.3117
7.3075
7.2924
7.2646
7.2604
7.2557
7.2499
7.2433
7.2364
7.2303
7.2263
7.2217
7.2156
7.0562
7.0370
7.0177
6.5338
6.5152
6.4536
6.4403
6.4202
4.2885
4.0405
2.2434
H
N
4.19
1.20
1.00
1.01
1.97
2.01
0.85
3.01
11.0
10.0
9.0
8.0
7.0
6.0
5.0
4.0
3.0
2.0
1.0
0.0
-1.0
f1 (ppm)

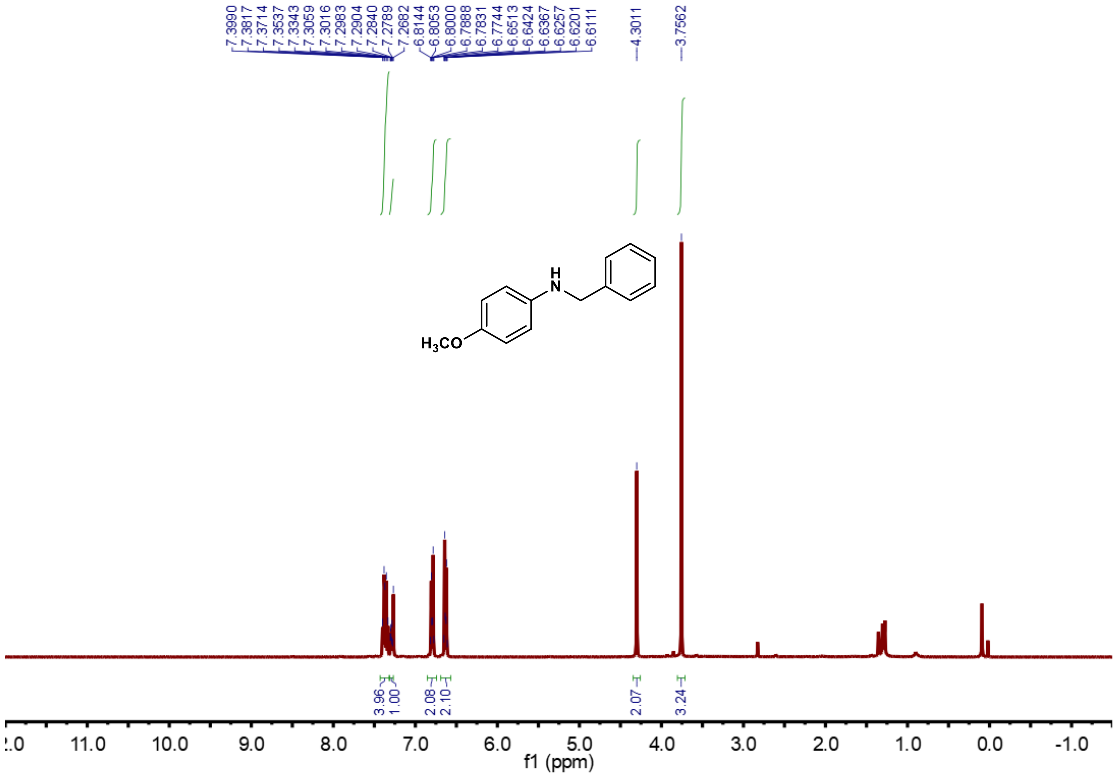
H
N
H_3CO
f1 (ppm)

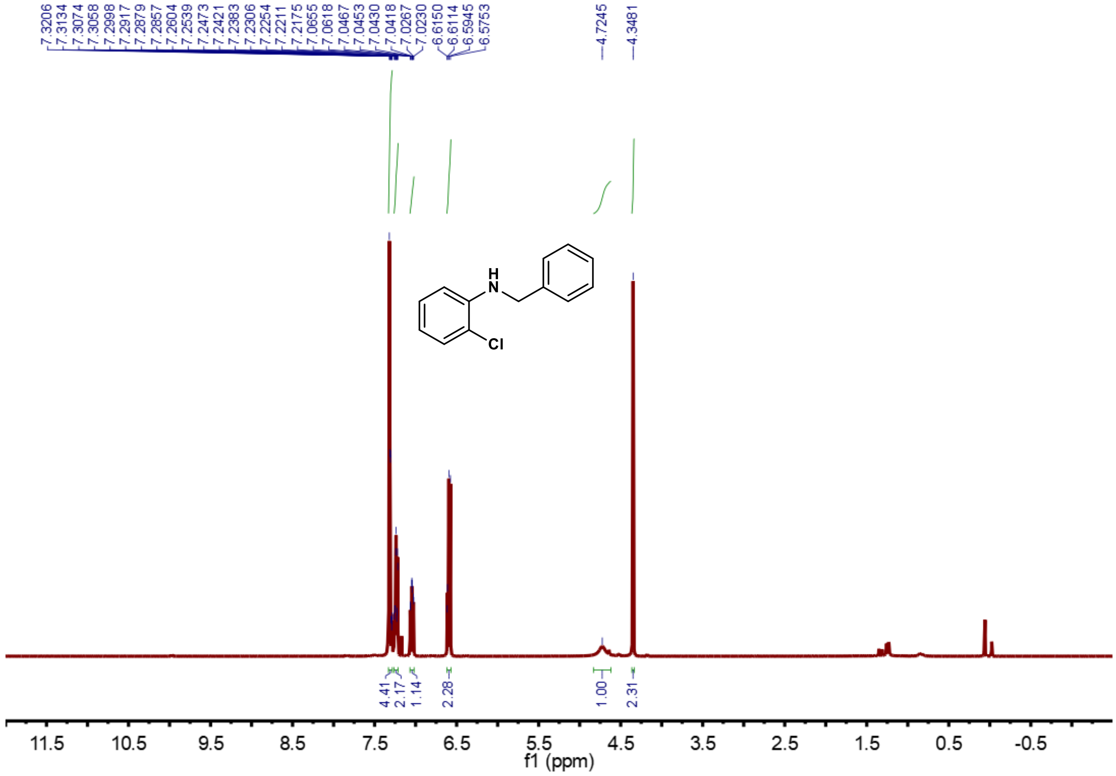
H
N
Cl
f1 (ppm)

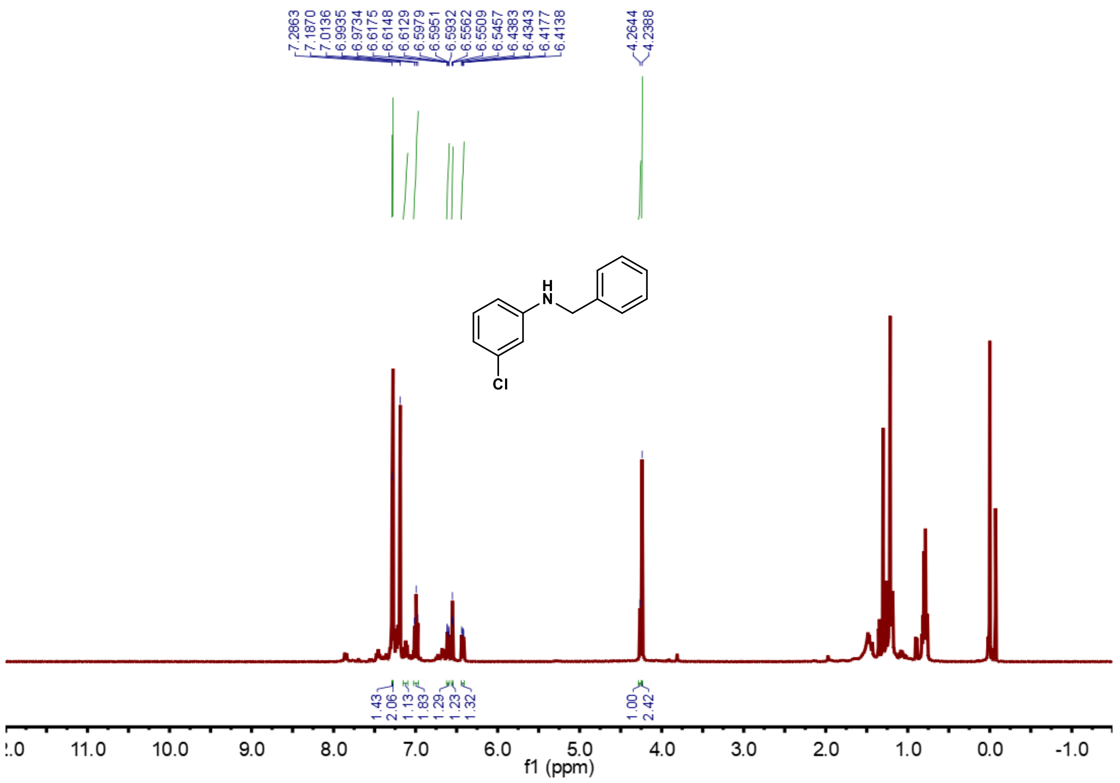

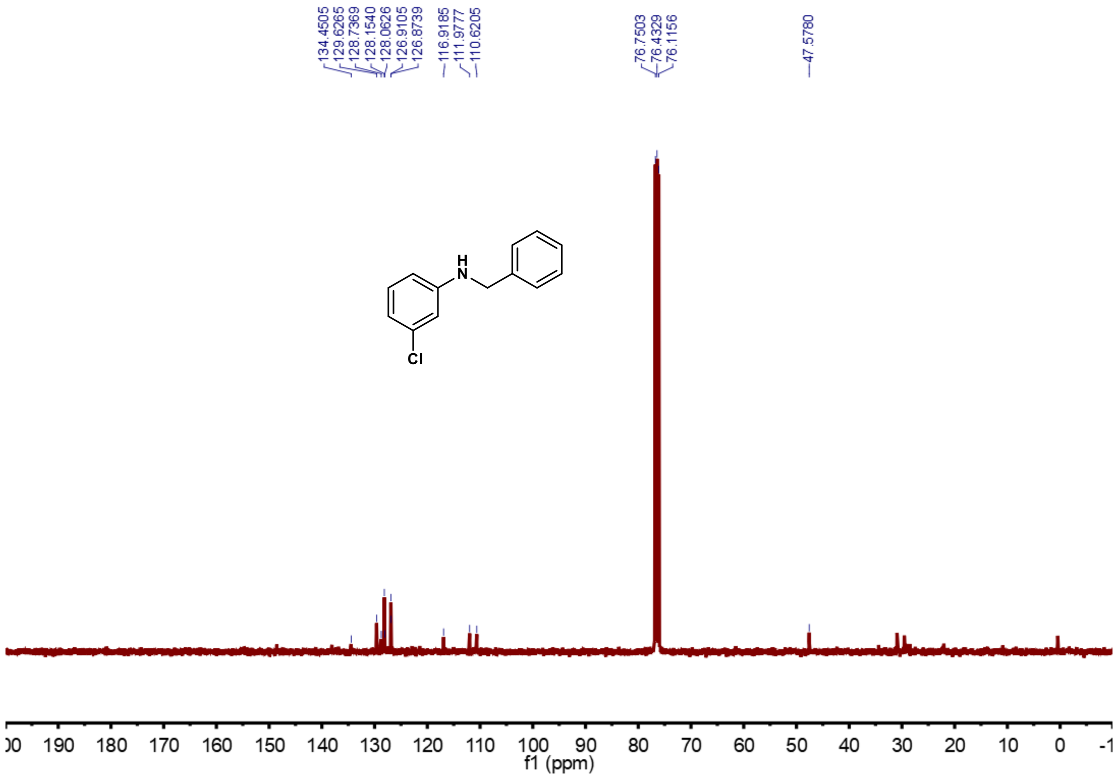

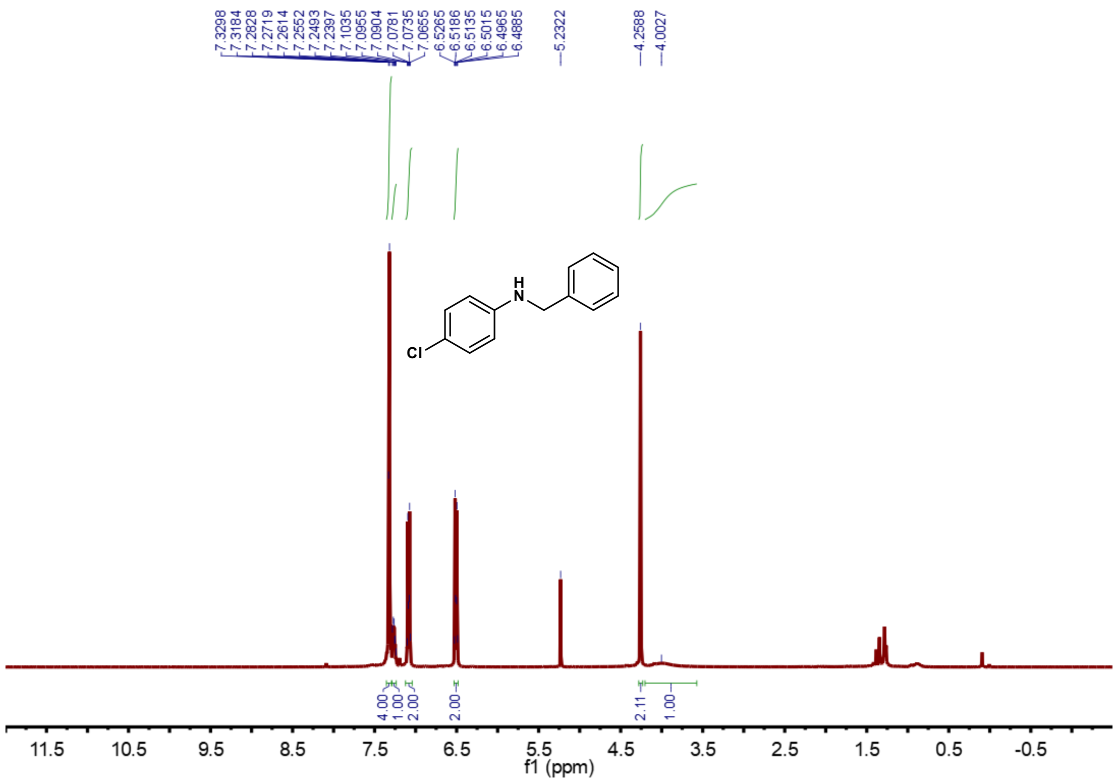

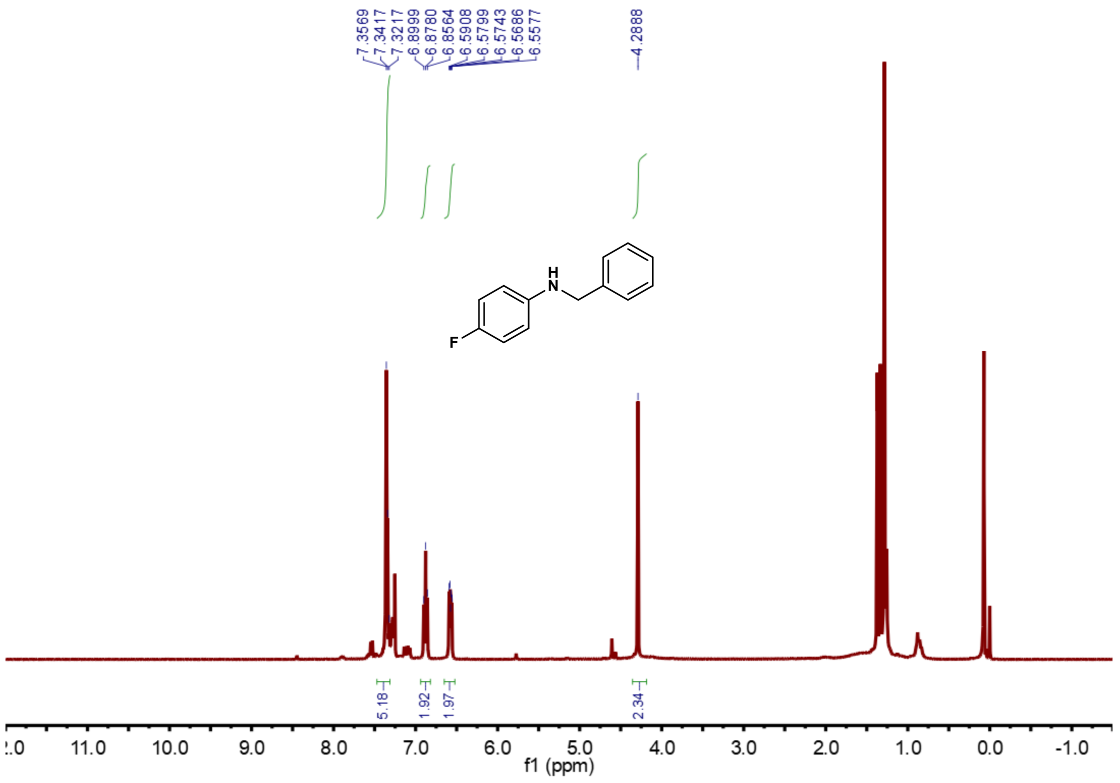

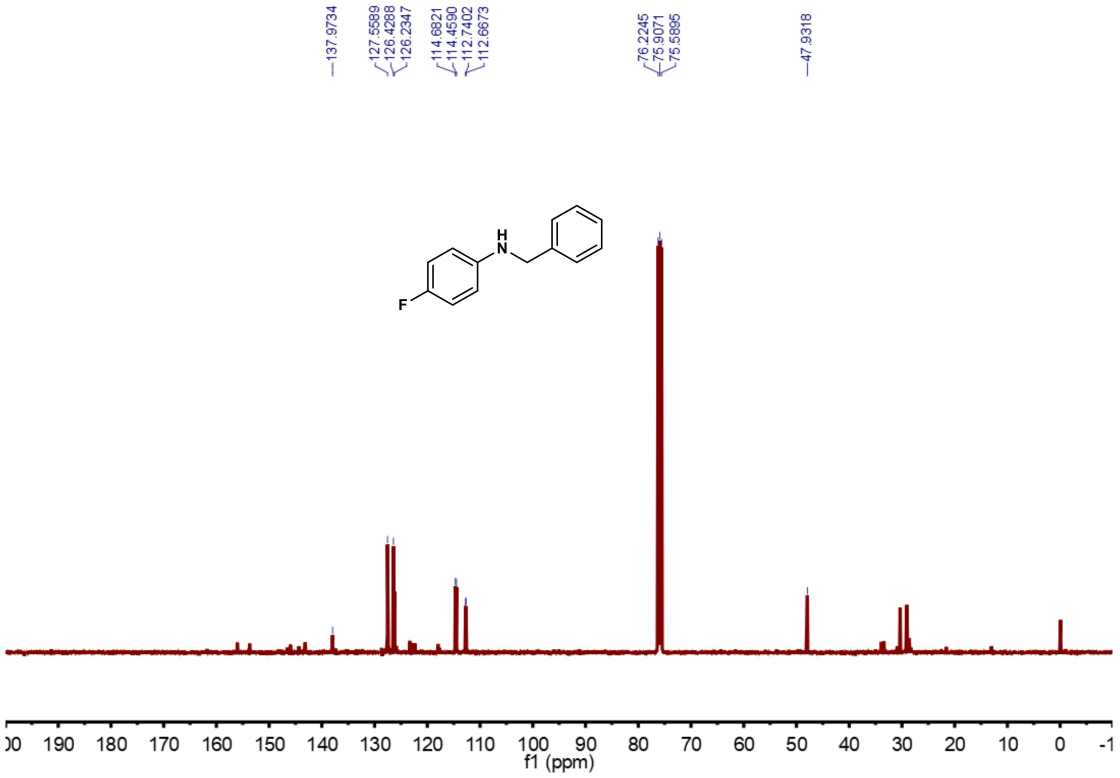

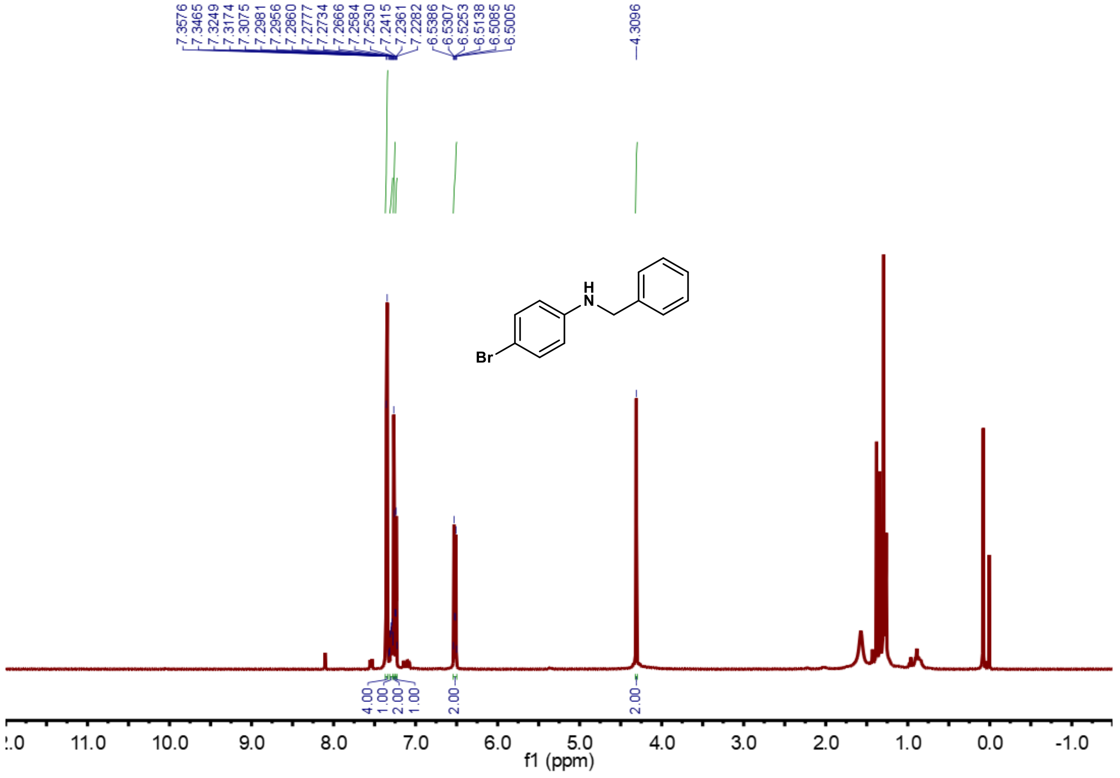

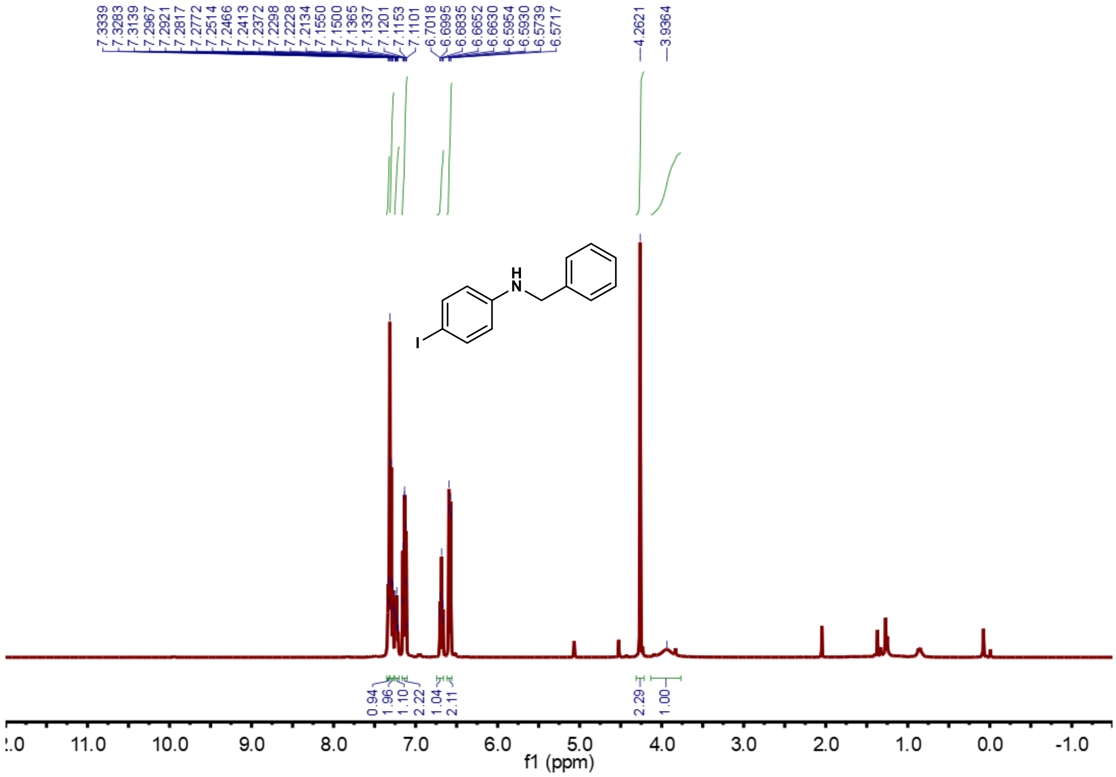
7.3339
7.3283
7.3139
7.2967
7.2921
7.2817
7.2772
7.2514
7.2466
7.2413
7.2372
7.2298
7.2228
7.2134
7.1550
7.1500
7.1365
7.1337
7.1201
7.1153
7.1101
6.7018
6.6995
6.6835
6.6652
6.6630
6.5954
6.5930
6.5739
6.5717
4.2621
3.9364
H
N
I
0.94
1.96
1.10
2.22
1.04
2.11
2.29
1.00
11.0
10.0
9.0
8.0
7.0
6.0
5.0
4.0
3.0
2.0
1.0
0.0
-1.0
f1 (ppm)

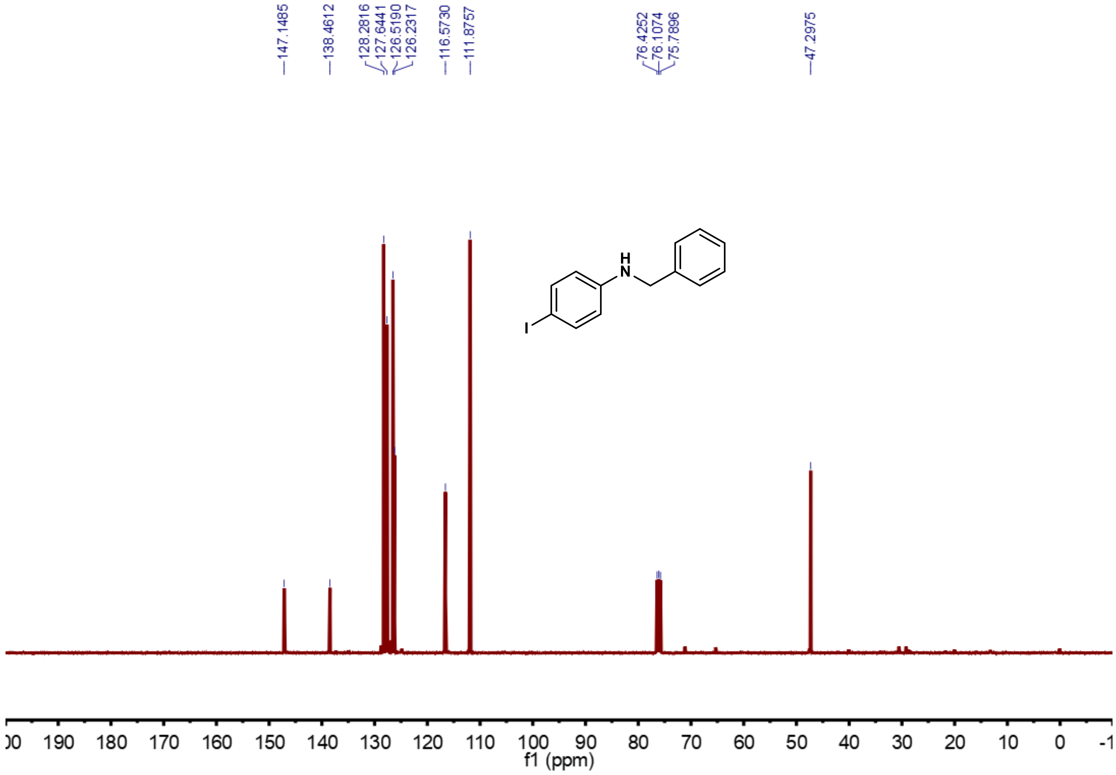
147.1485
138.4612
128.2816
127.6441
126.5190
126.2317
116.5730
111.8757
76.4252
76.1074
75.7896
47.2975
H
N
I
190
180
170
160
150
140
130
120
110
100
90
80
70
60
50
40
30
20
10
0
f1 (ppm)

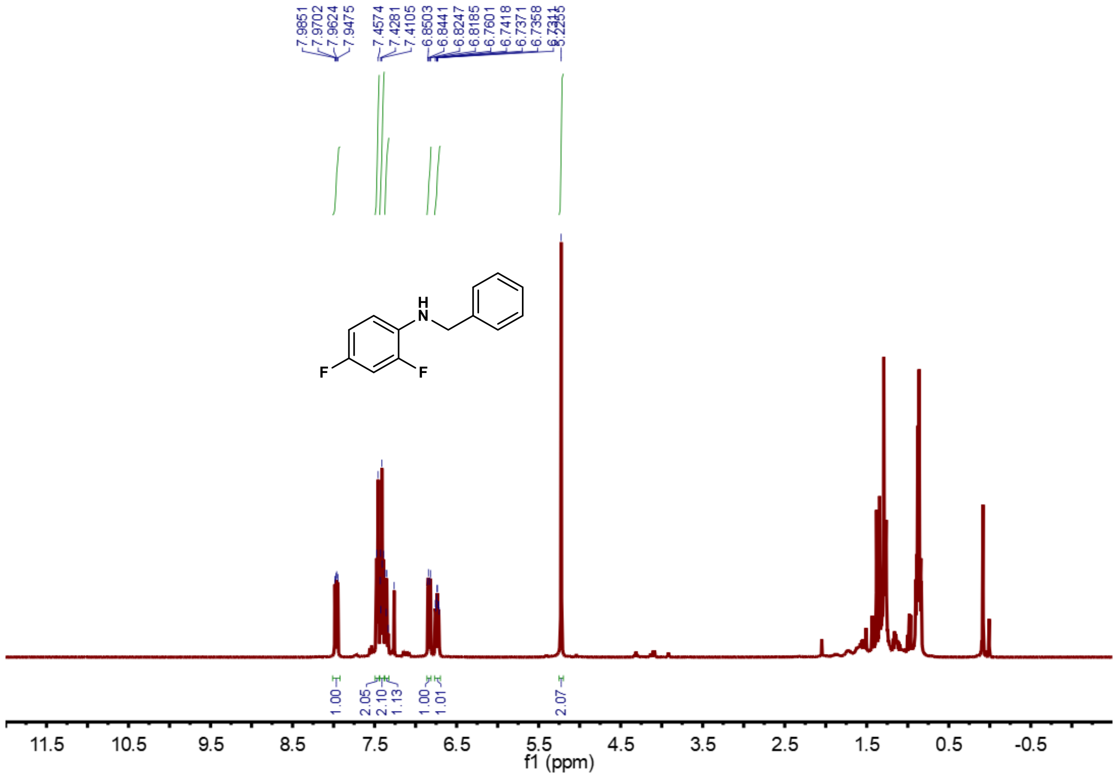

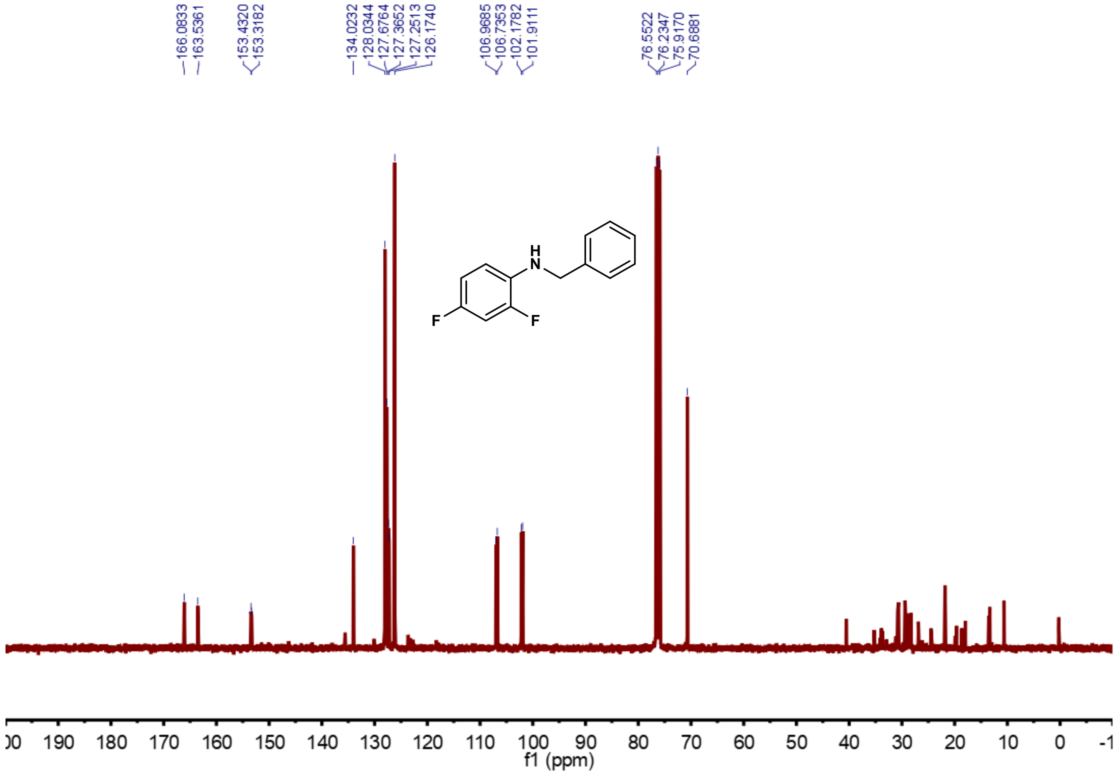

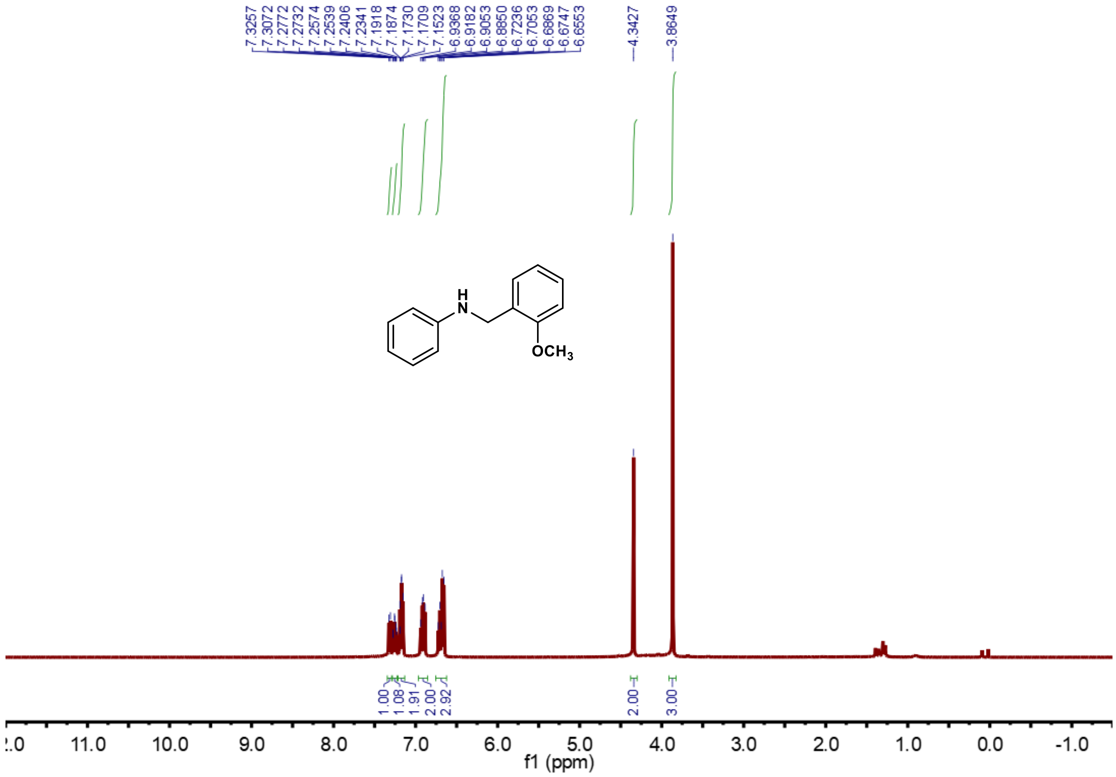

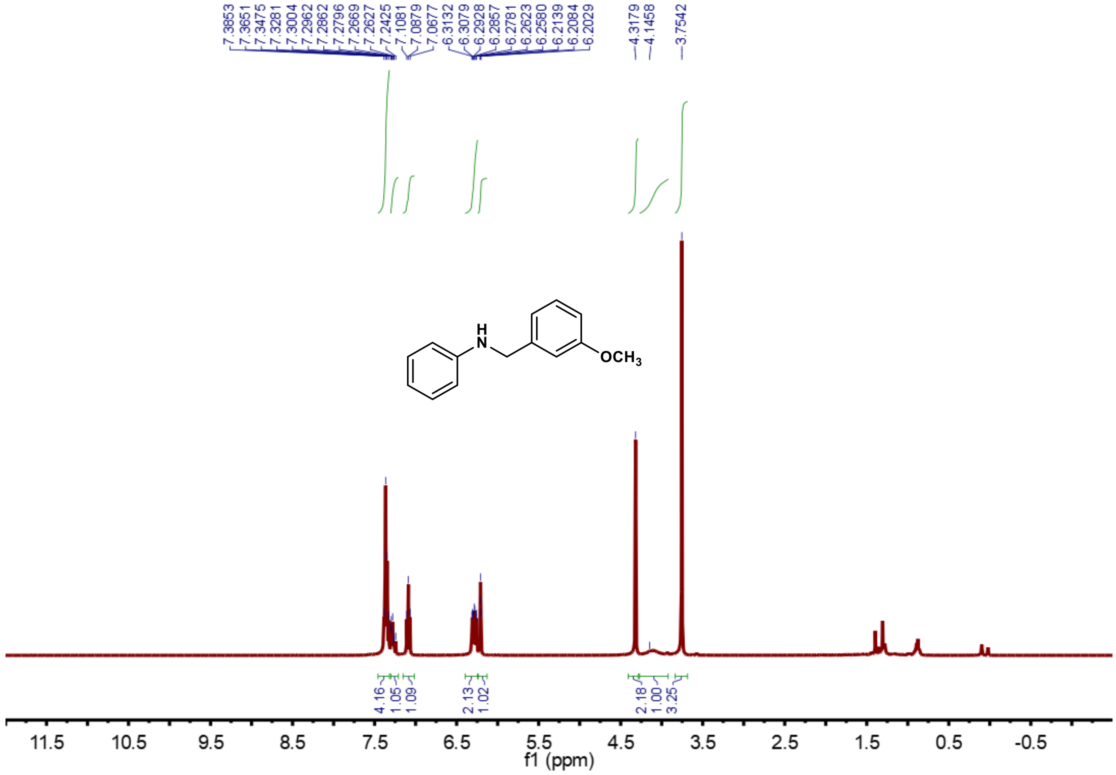

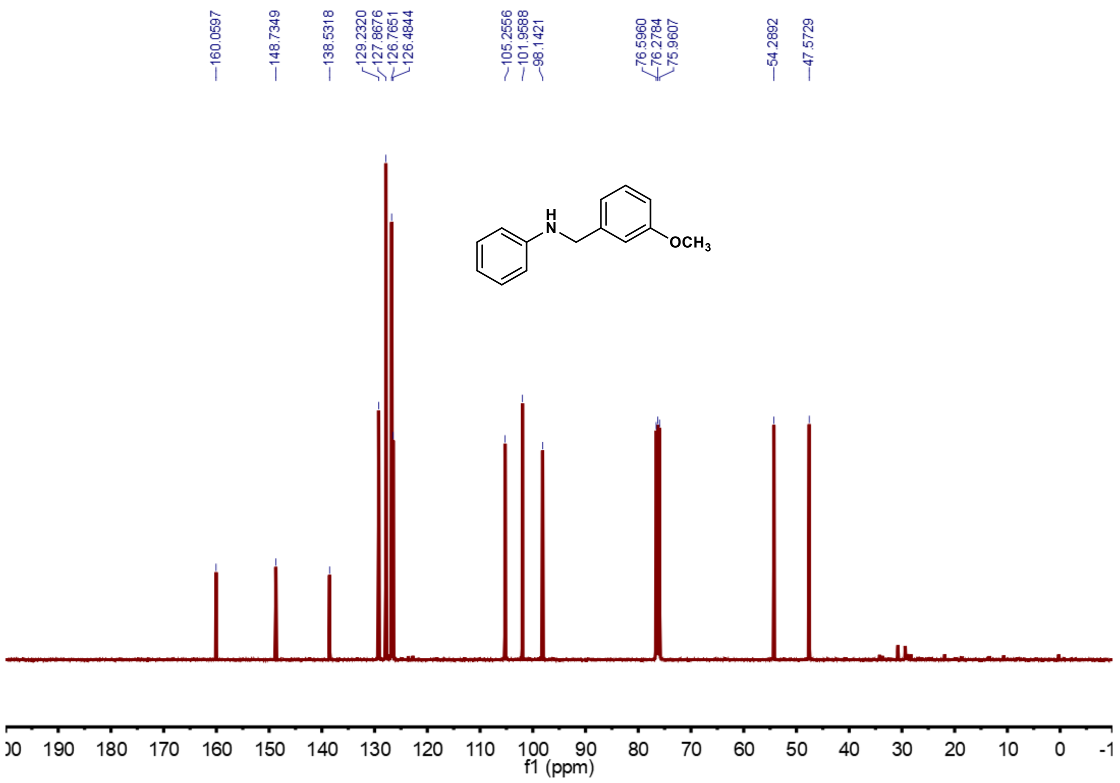
160.0597
148.7349
138.5318
129.2320
127.8676
126.7651
126.4844
105.2556
101.9588
98.1421
76.5960
76.2784
75.9607
54.2892
47.5729
H
N
OCH3
f1 (ppm)

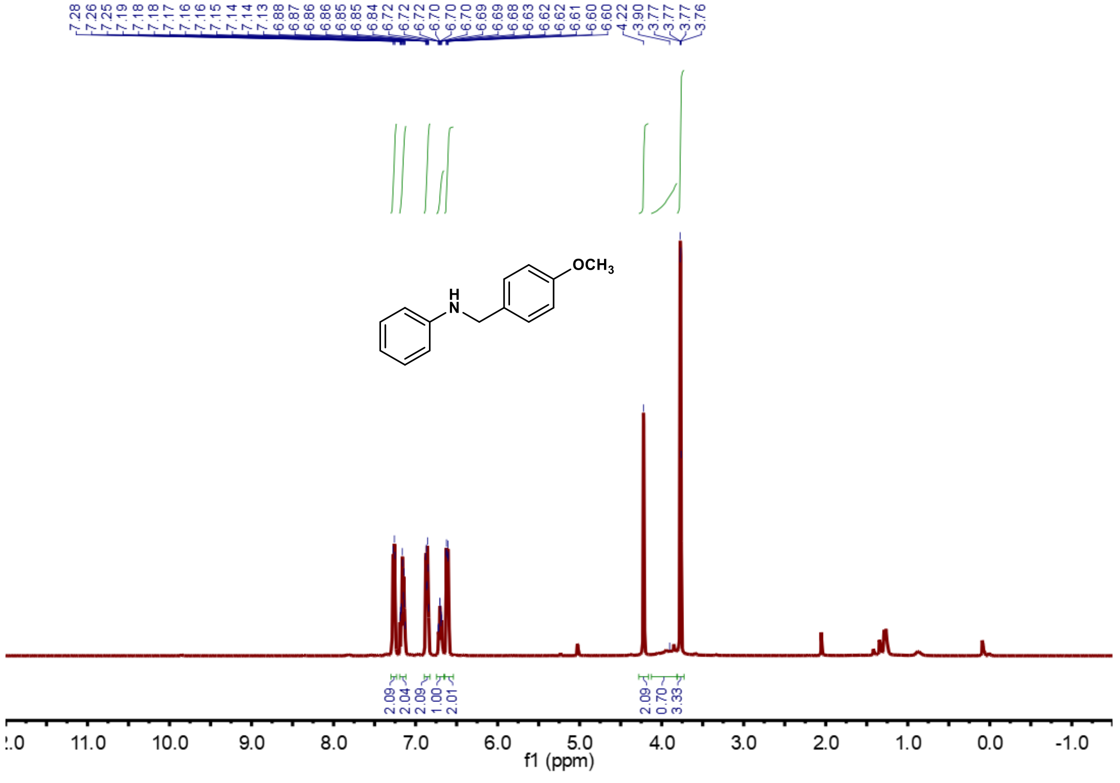
7.28
7.26
7.25
7.19
7.18
7.17
7.16
7.15
7.14
7.13
6.88
6.87
6.86
6.86
6.85
6.84
6.72
6.72
6.70
6.70
6.69
6.68
6.63
6.62
6.61
6.60
6.60
4.22
3.90
3.77
3.77
3.77
3.76
OCH3
H
N
2.09
2.04
2.09
1.00
2.01
2.09
0.70
3.33
f1 (ppm)

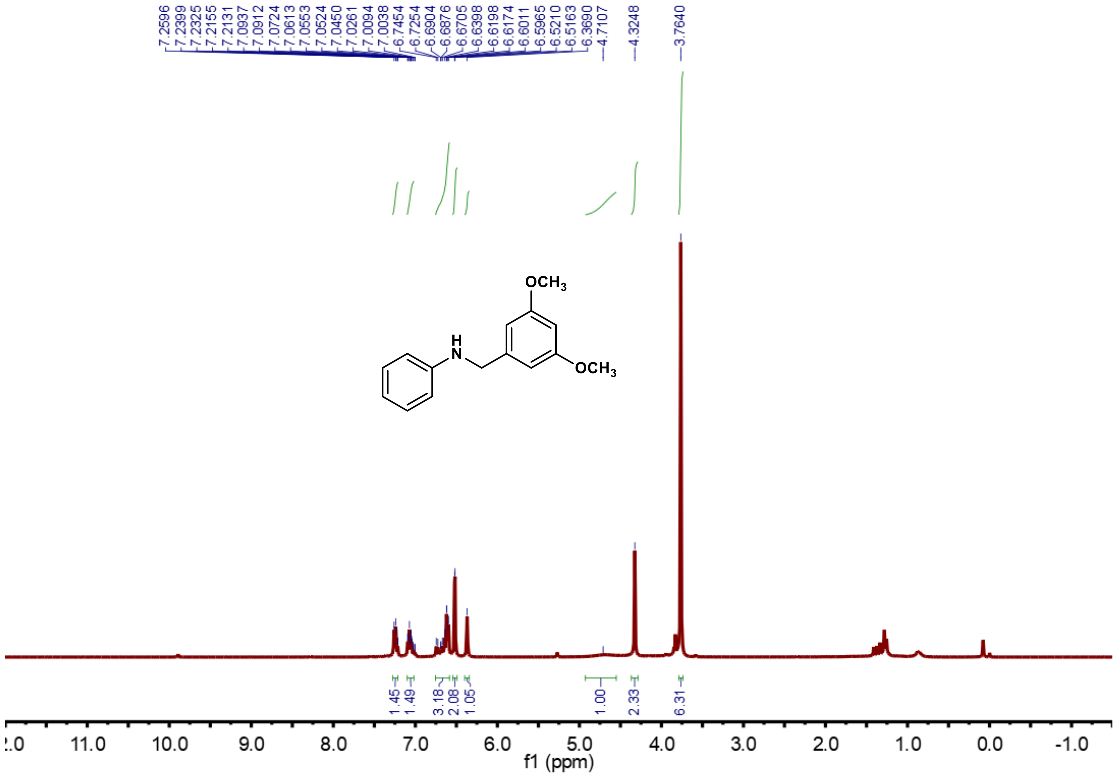
7.2596
7.2399
7.2325
7.2155
7.2131
7.0937
7.0912
7.0724
7.0613
7.0553
7.0524
7.0450
7.0261
7.0094
7.0038
6.7454
6.7254
6.6904
6.6876
6.6705
6.6398
6.6198
6.6174
6.6011
6.5965
6.5210
6.5163
6.3690
4.7107
4.3248
3.7640
OCH3
H
N
OCH3
1.45
1.49
3.18
2.08
1.05
1.00
2.33
6.31
11.0
10.0
9.0
8.0
7.0
6.0
5.0
4.0
3.0
2.0
1.0
0.0
-1.0
f1 (ppm)

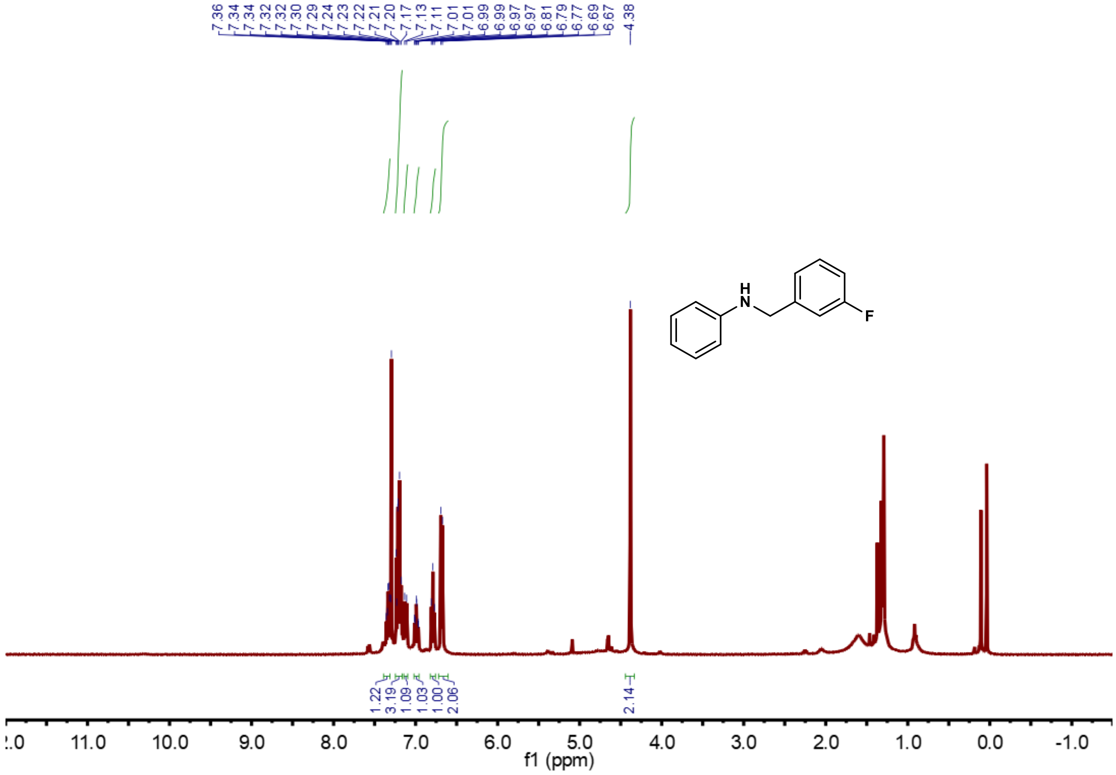
7.36
7.34
7.32
7.30
7.29
7.24
7.23
7.22
7.21
7.20
7.17
7.13
7.11
7.01
6.99
6.97
6.81
6.79
6.69
6.67
4.38
H
N
F
1.22
3.19
1.09
1.03
1.00
2.06
2.14
11.0
10.0
9.0
8.0
7.0
6.0
5.0
4.0
3.0
2.0
1.0
0.0
-1.0
f1 (ppm)

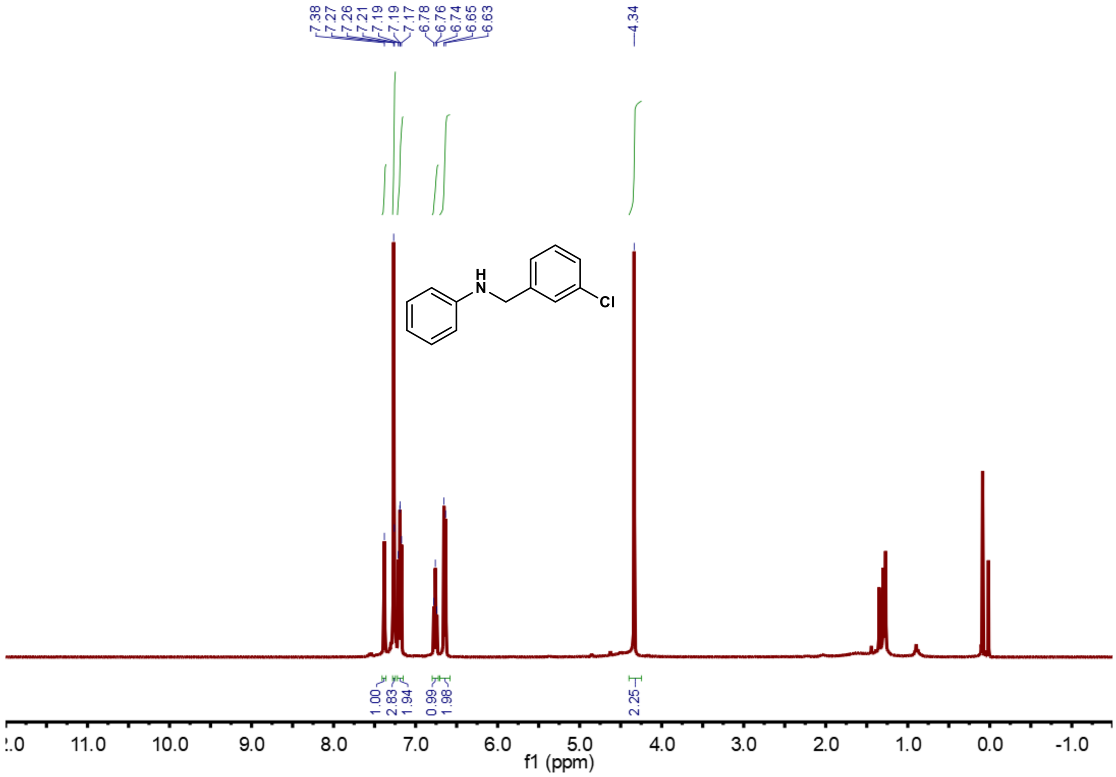

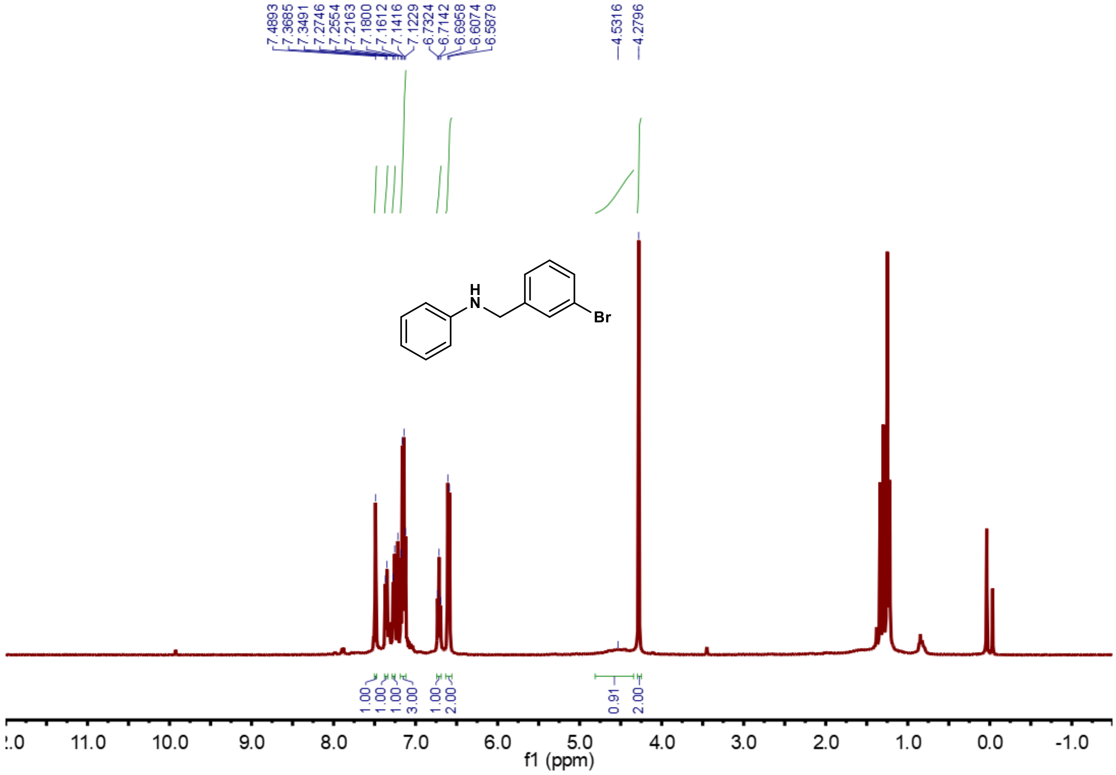

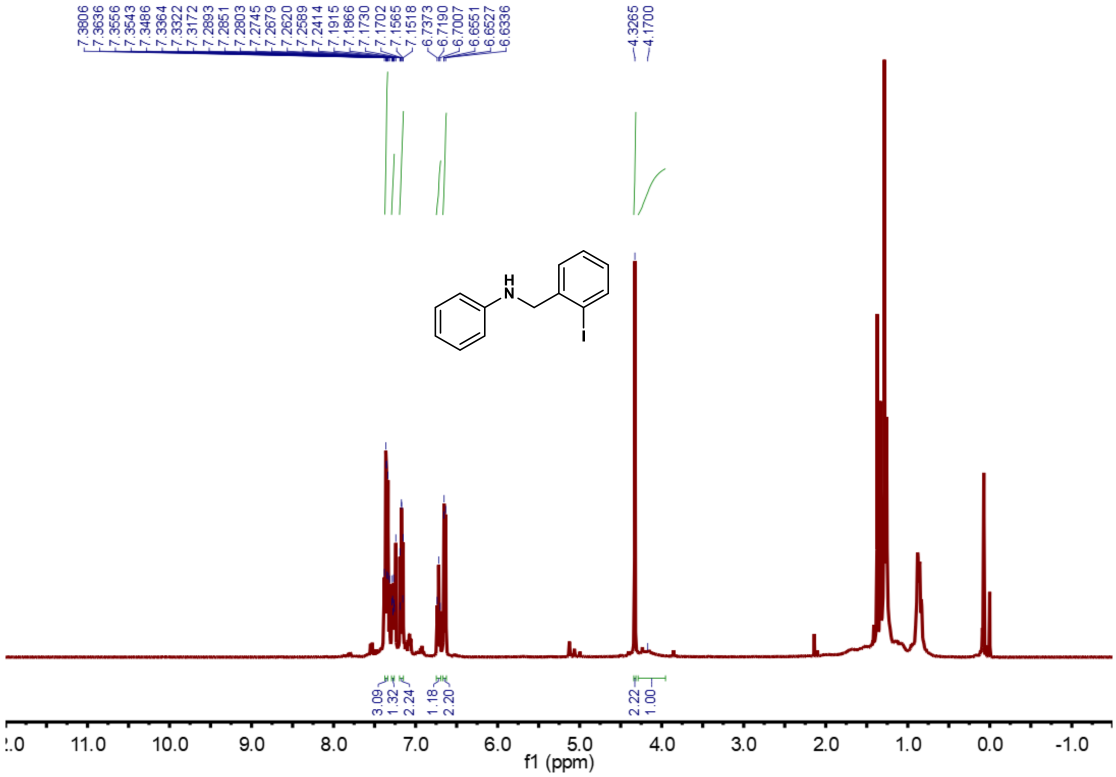

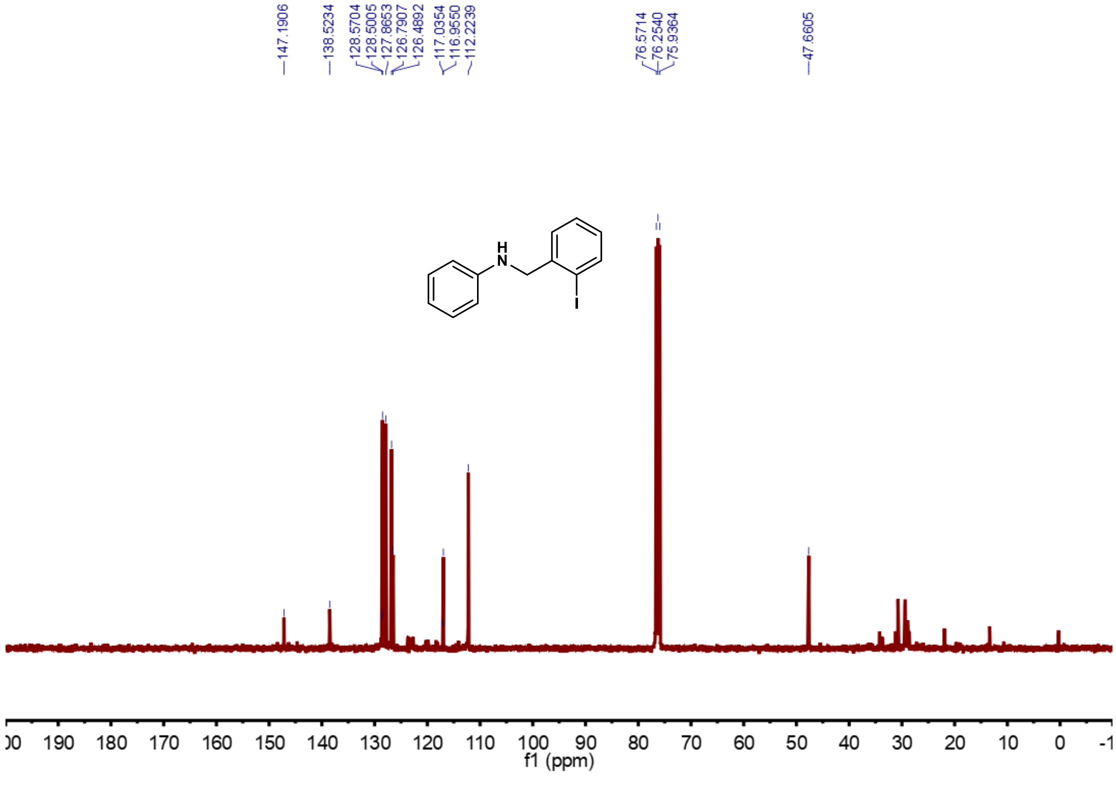

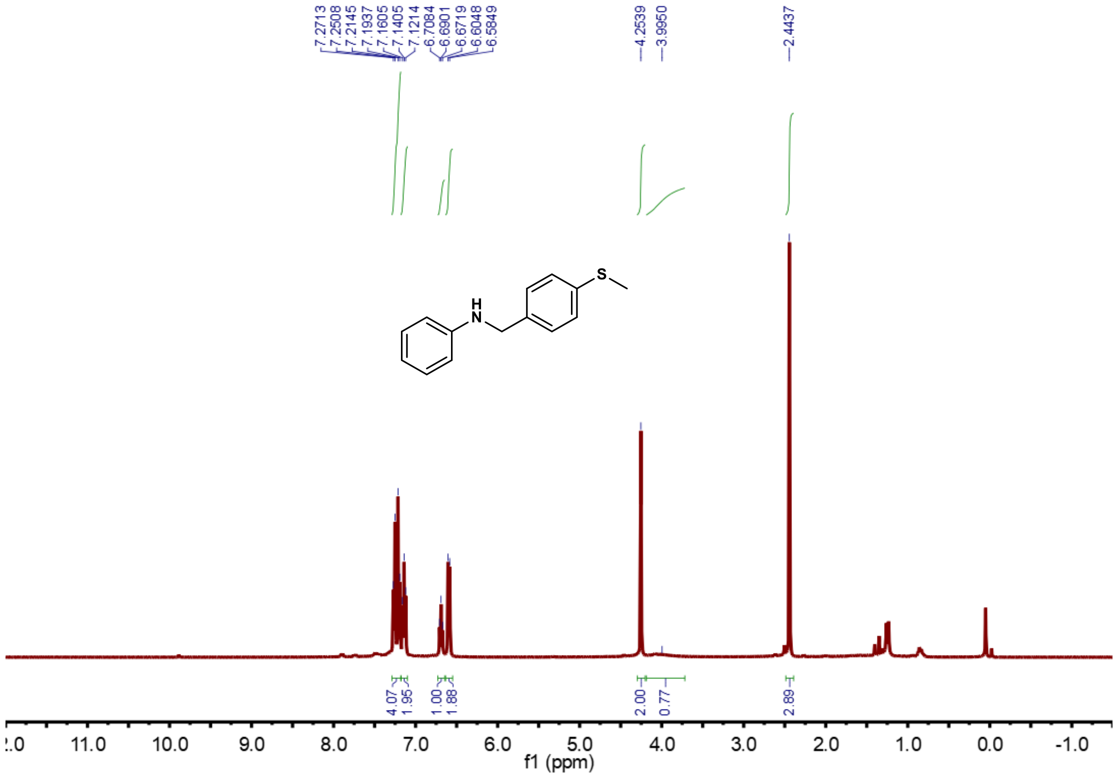

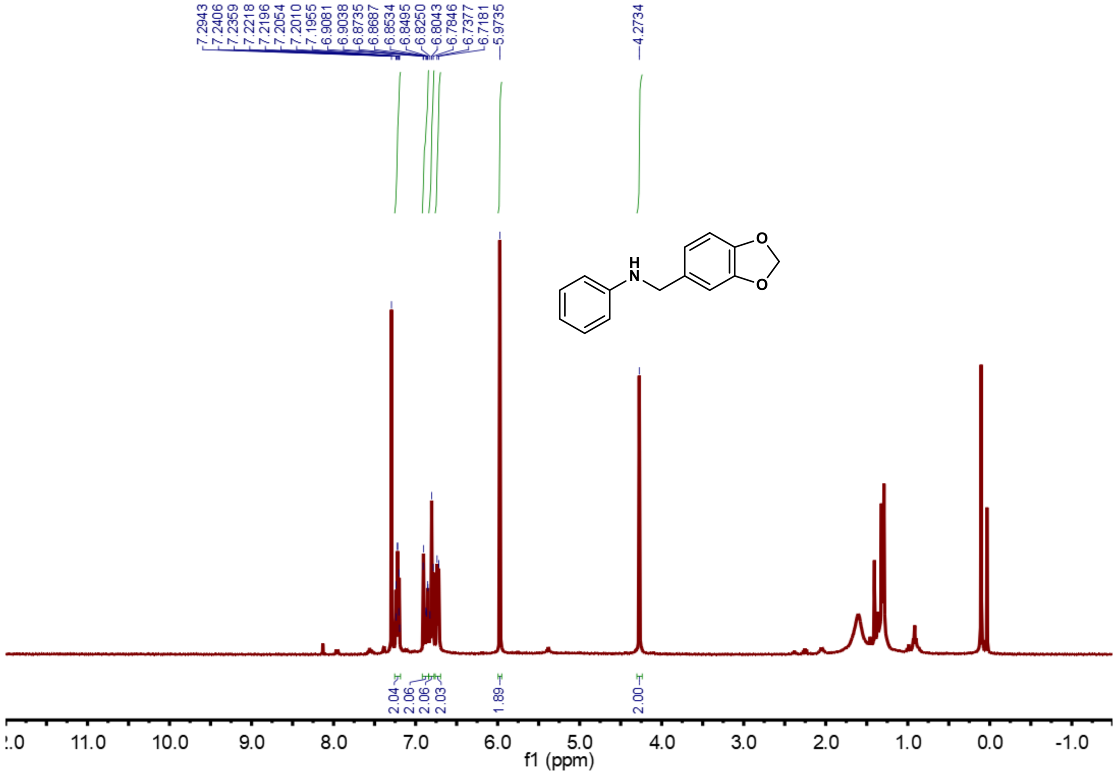

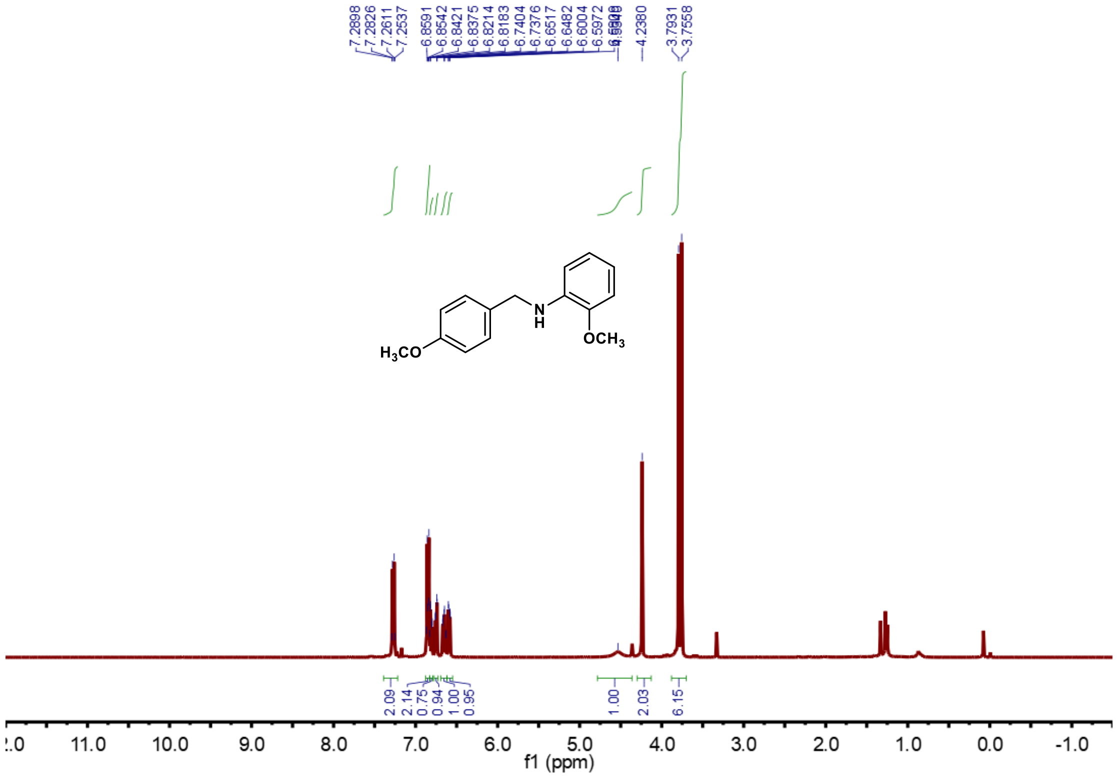

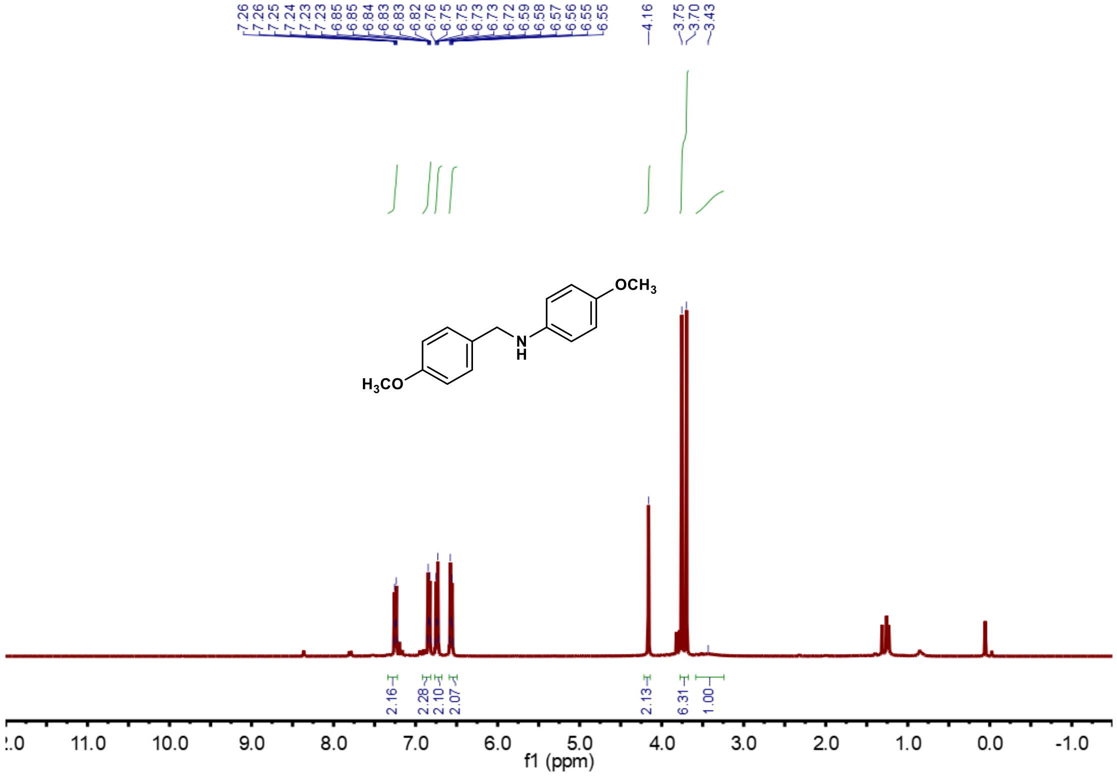

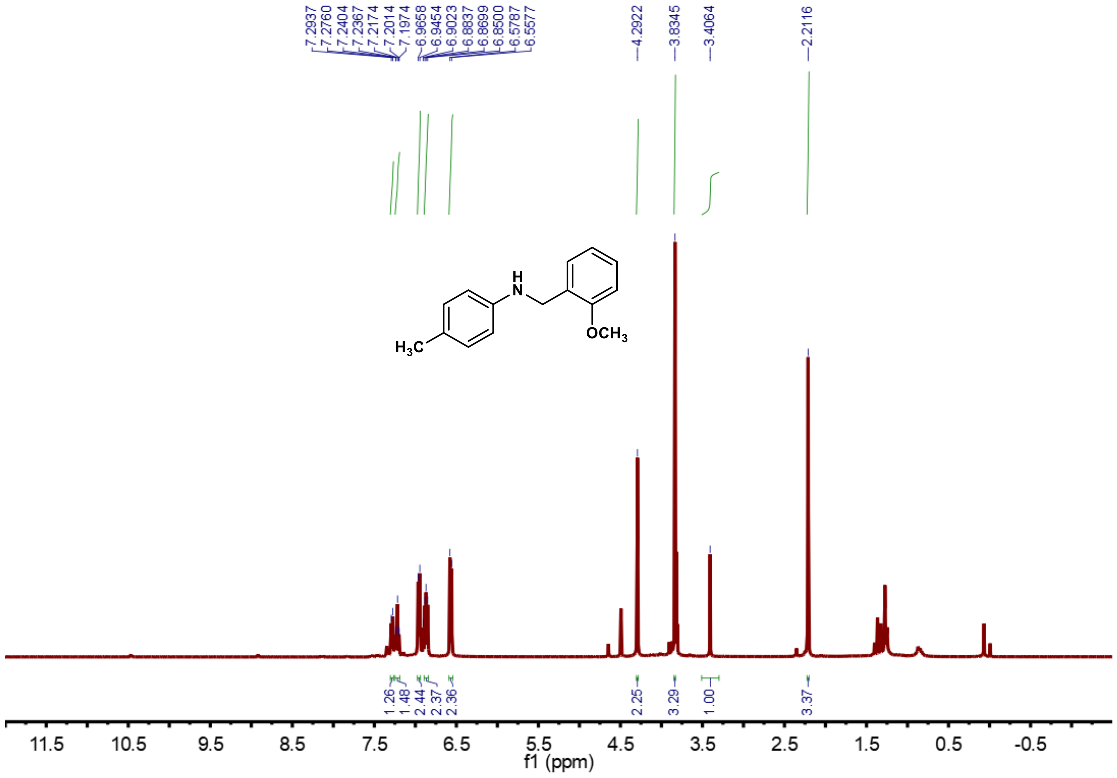

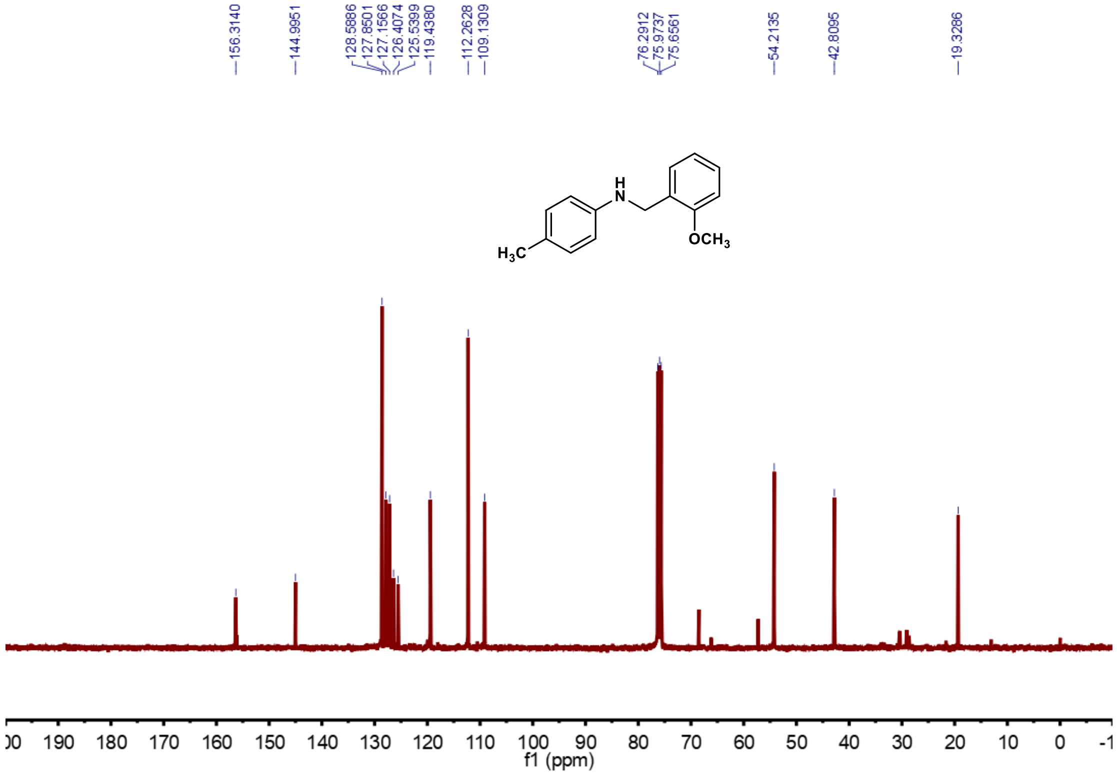

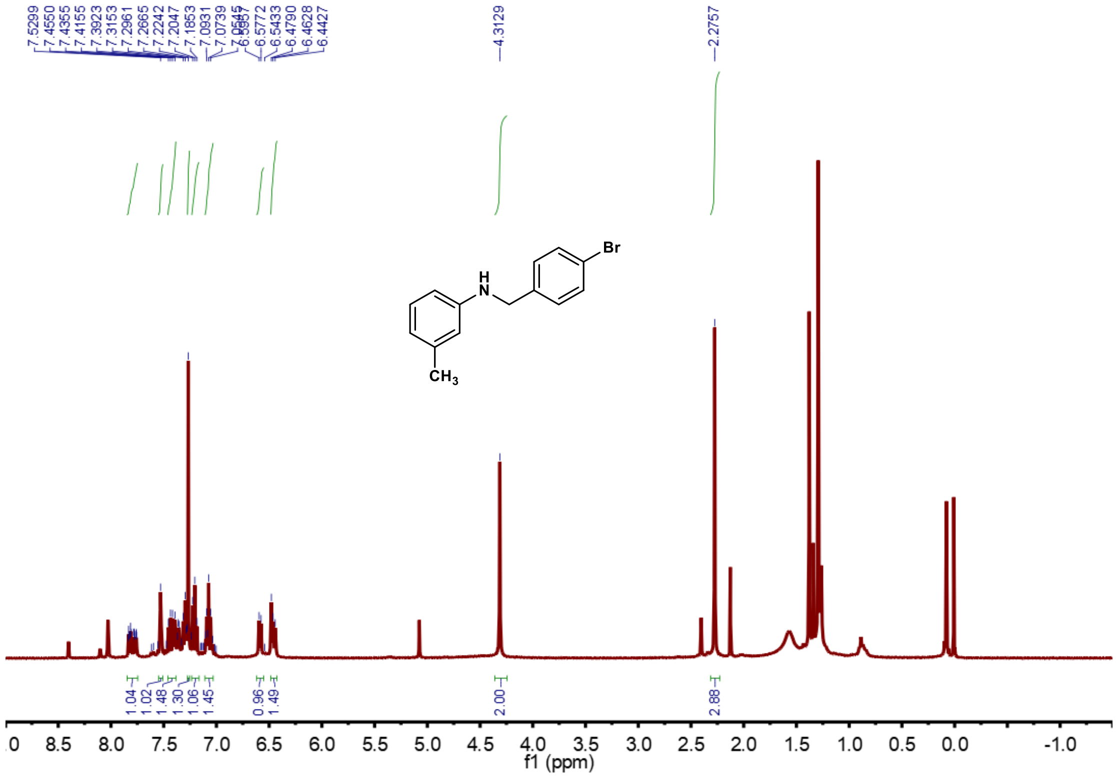
7.5299
7.4550
7.4355
7.4155
7.3923
7.3153
7.2961
7.2665
7.2242
7.2047
7.1853
7.0931
7.0739
6.5772
6.5433
6.4790
6.4628
6.4427
4.3129
2.2757
Br
H
N
CH3
1.04
1.02
1.48
1.30
1.06
1.45
0.96
1.49
2.00
2.88
8.5
8.0
7.5
7.0
6.5
6.0
5.5
5.0
4.5
4.0
3.5
3.0
2.5
2.0
1.5
1.0
0.5
0.0
-1.0
f1 (ppm)

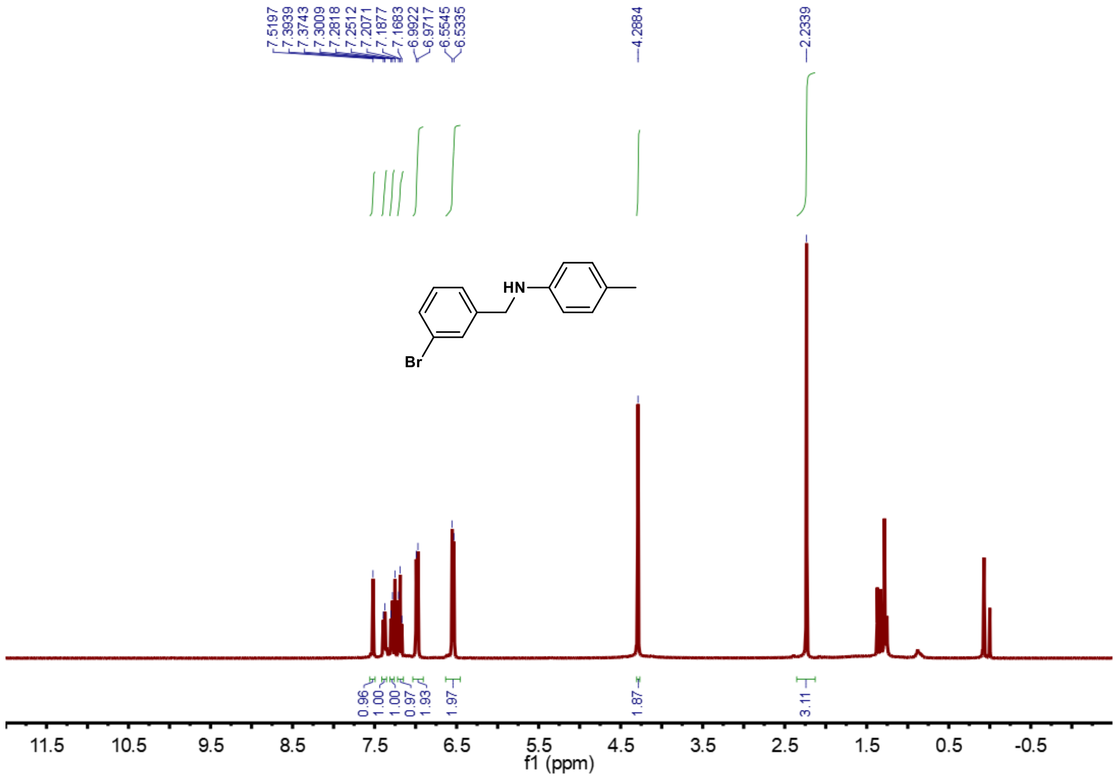
7.5197
7.3939
7.3743
7.3009
7.2818
7.2512
7.2071
7.1877
7.1683
6.9922
6.9717
6.5545
6.5335
4.2884
2.2339
HN
Br
0.96
1.00
1.00
0.97
1.93
1.97
1.87
3.11
11.5
10.5
9.5
8.5
7.5
6.5
5.5
4.5
3.5
2.5
1.5
0.5
-0.5
f1 (ppm)

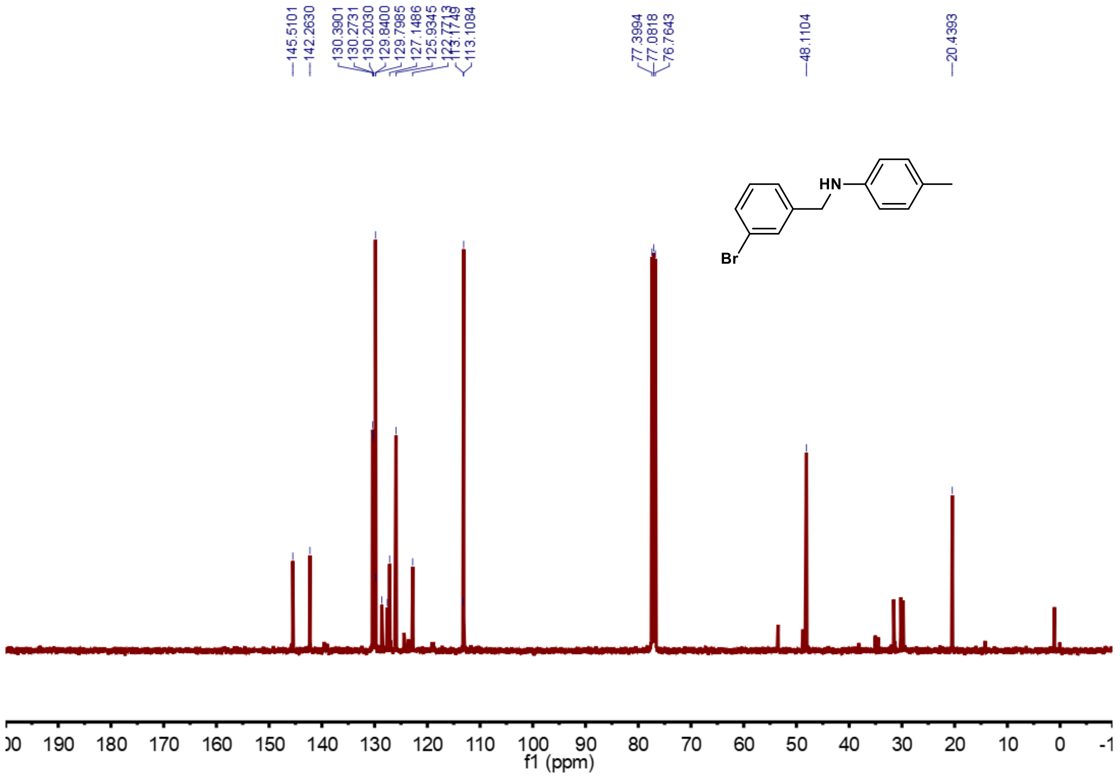
145.5101
142.2630
130.3901
130.2731
130.2030
129.8400
129.7985
127.1486
125.9345
122.7713
113.1749
113.1084
77.3994
77.0818
76.7643
48.1104
20.4393
HN
Br
f1 (ppm)

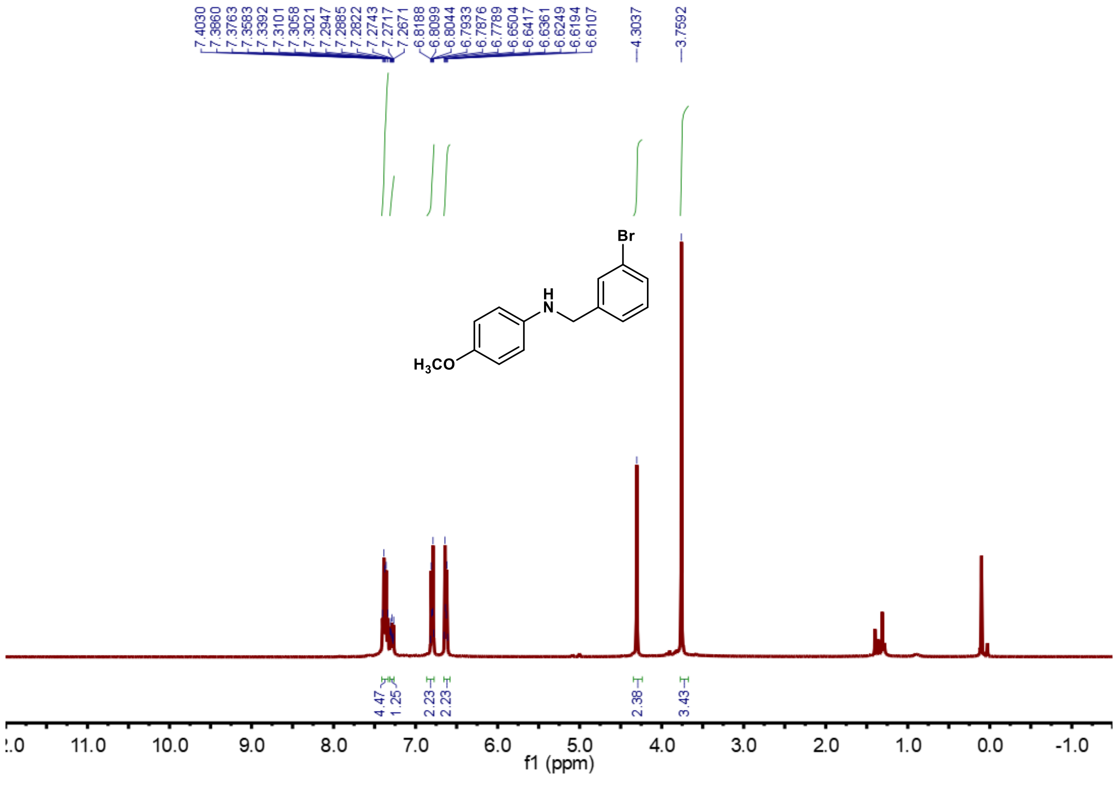
7.4030
7.3860
7.3763
7.3583
7.3392
7.3101
7.3058
7.3021
7.2947
7.2885
7.2822
7.2743
7.2717
7.2671
6.8188
6.8099
6.8044
6.7933
6.7876
6.7789
6.6504
6.6417
6.6361
6.6249
6.6194
6.6107
4.3037
3.7592
Br
H
N
H_3CO
4.47
1.25
2.23
2.23
2.38
3.43
f1 (ppm)

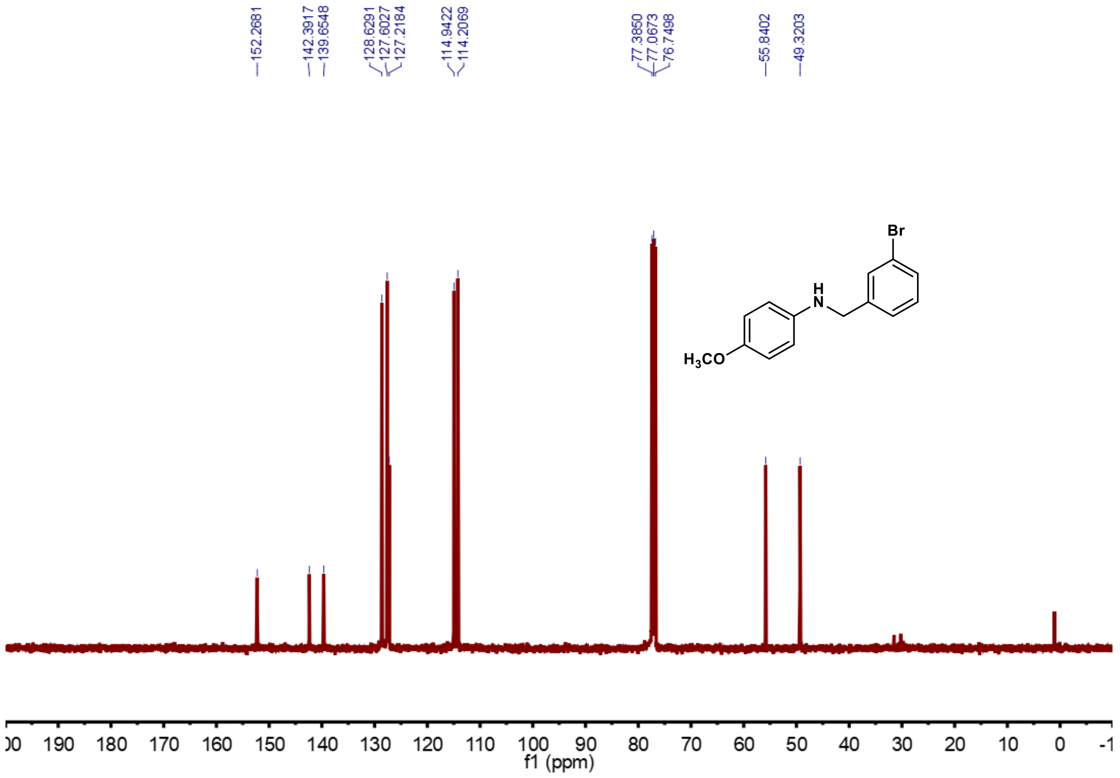
152.2681
142.3917
139.6548
128.6291
127.6027
127.2184
114.9422
114.2069
77.3850
77.0673
76.7498
55.8402
49.3203
Br
H
N
H3CO
f1 (ppm)

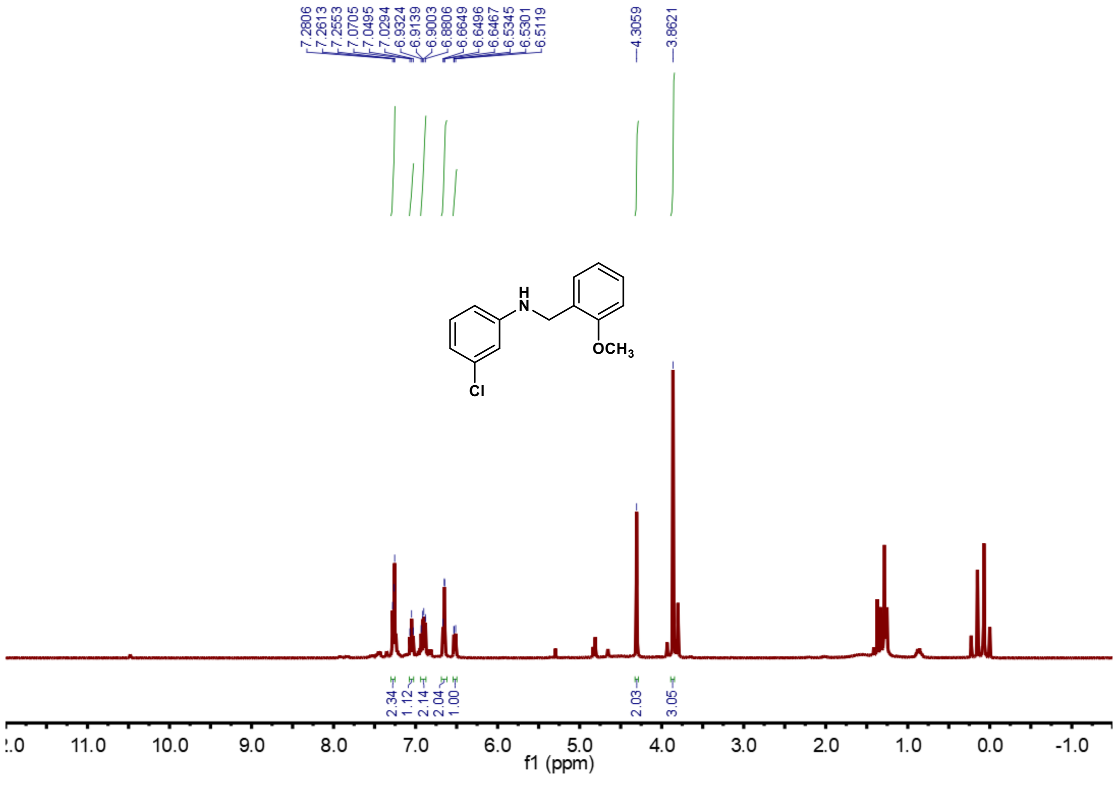
4.3059
3.8621
H
N
OCH3
Cl
2.34
1.12
2.14
2.04
1.00
2.03
3.05
f1 (ppm)

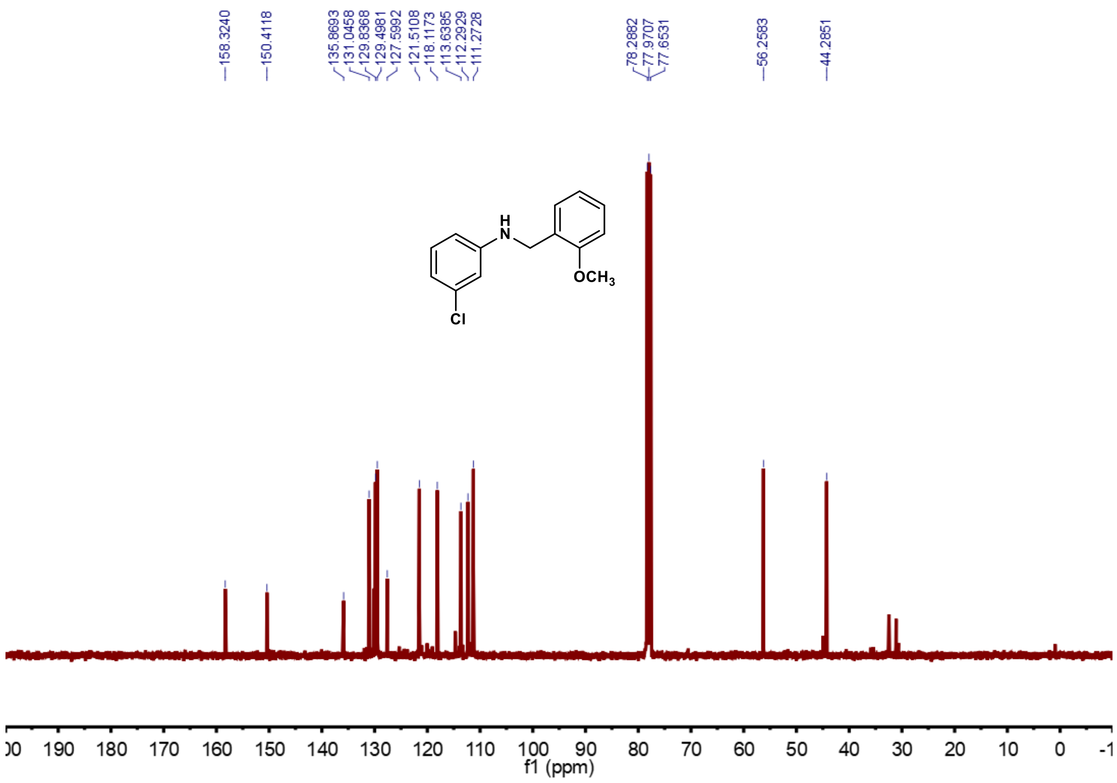
158.3240
150.4118
135.8693
131.0458
129.8368
129.4981
127.5992
121.5108
118.1173
113.6385
112.2929
111.2728
78.2882
77.9707
77.6531
56.2583
44.2851
H
N
OCH3
Cl
f1 (ppm)

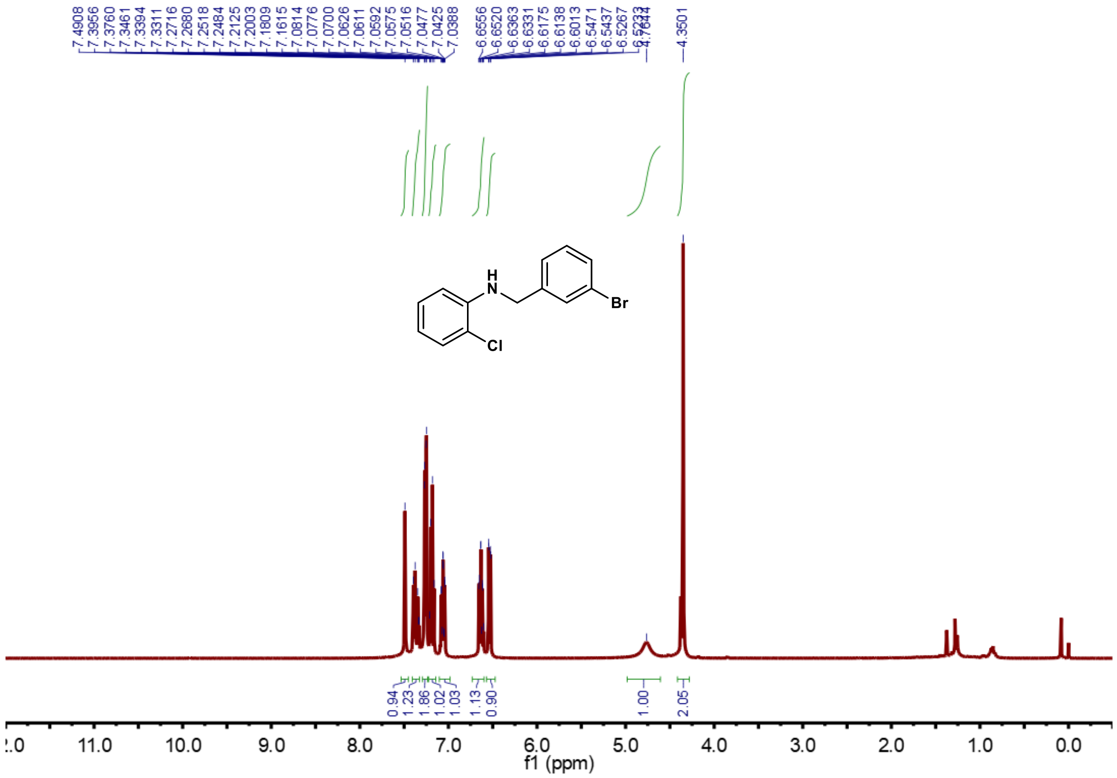
7.4908
7.3956
7.3760
7.3461
7.3394
7.3311
7.2716
7.2680
7.2518
7.2484
7.2125
7.2003
7.1809
7.1615
7.0814
7.0776
7.0700
7.0626
7.0611
7.0592
7.0575
7.0516
7.0477
7.0425
7.0388
6.6556
6.6520
6.6363
6.6331
6.6175
6.6138
6.6013
6.5471
6.5437
6.5267
6.5233
4.7644
4.3501
H
N
Br
Cl
0.94
1.23
1.86
1.02
1.03
1.13
0.90
1.00
2.05
f1 (ppm)

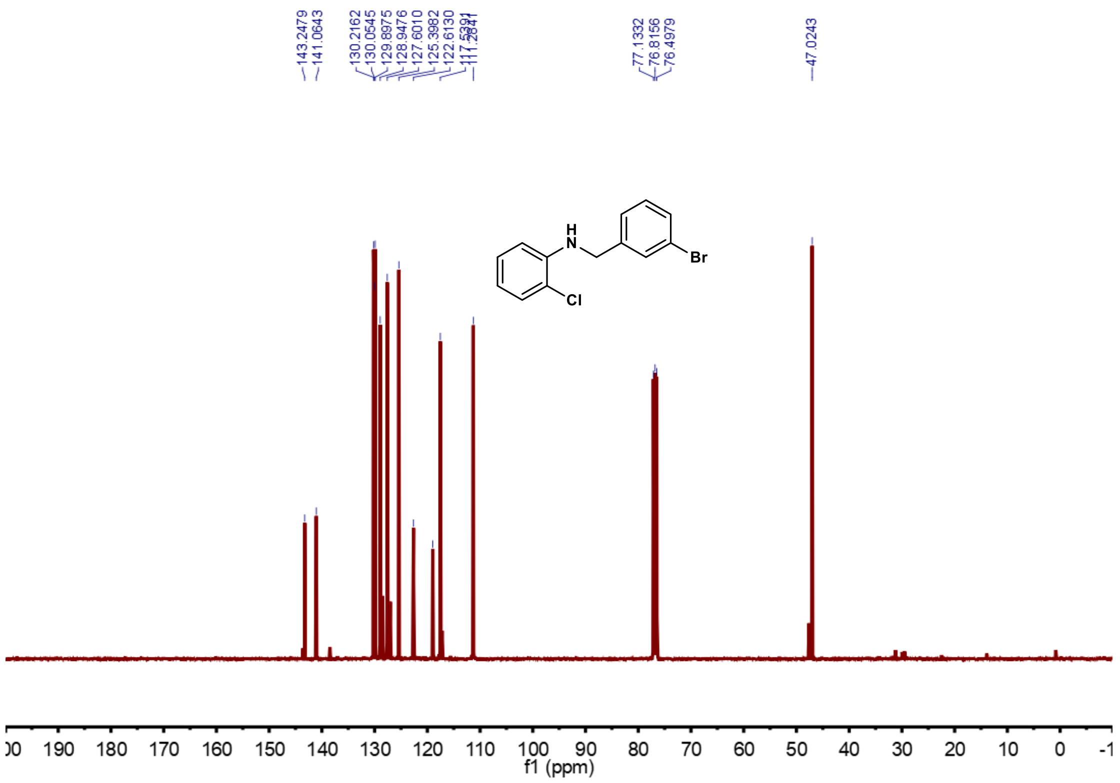

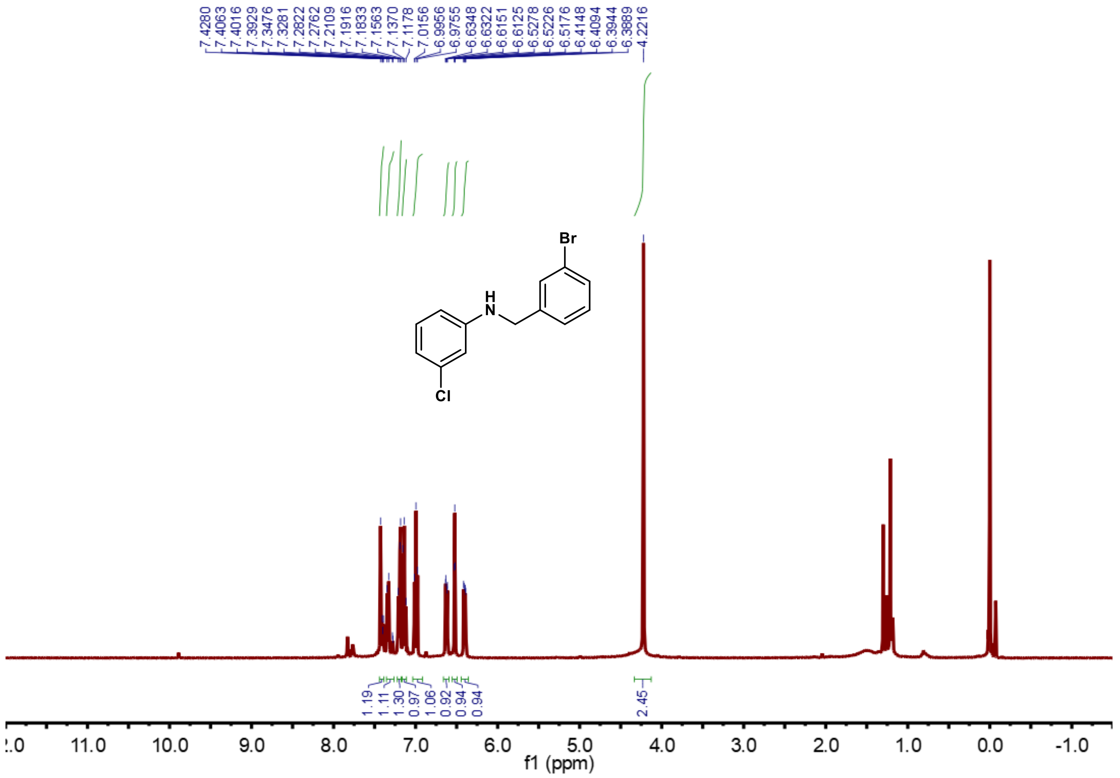

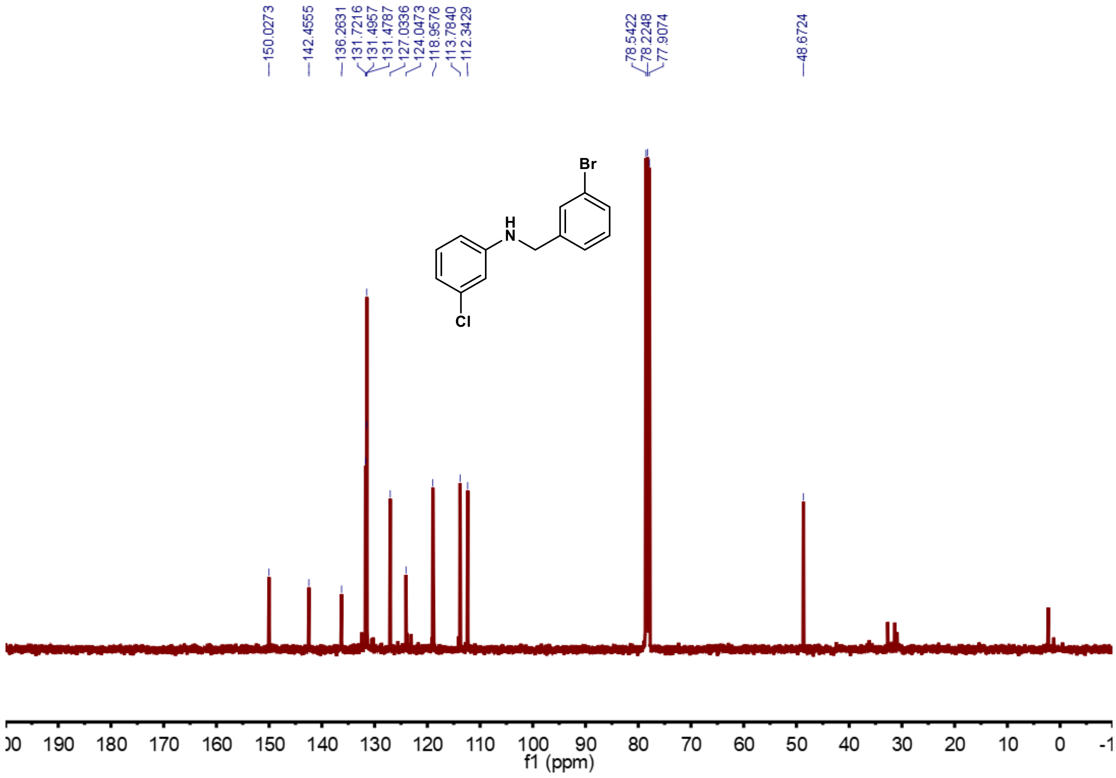
150.0273
142.4555
136.2631
131.7216
131.4957
131.4787
127.0336
124.0473
118.9576
113.7840
112.3429
78.5422
78.2248
77.9074
48.6724
Br
H
N
Cl
f1 (ppm)

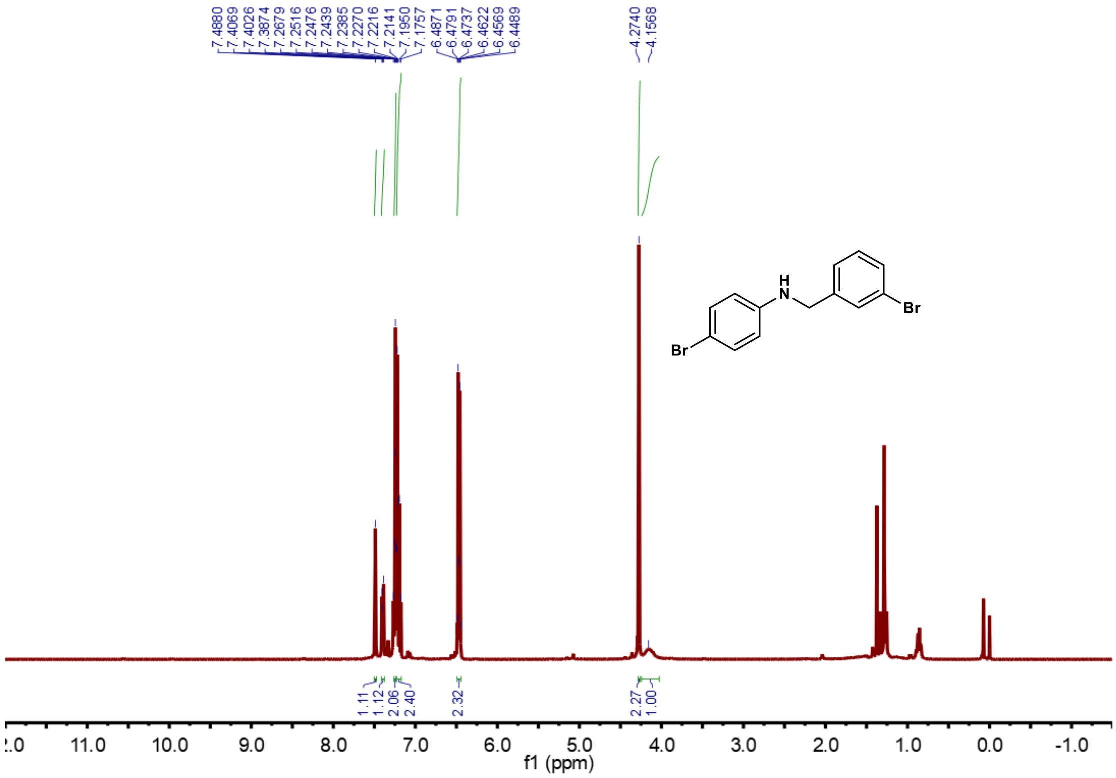
7.4880
7.4069
7.4026
7.3874
7.2679
7.2516
7.2476
7.2439
7.2385
7.2270
7.2216
7.2141
7.1950
7.1757
6.4871
6.4791
6.4737
6.4622
6.4569
6.4489
4.2740
4.1568
H
N
Br
Br
1.11
1.12
2.06
2.40
2.32
2.27
1.00
f1 (ppm)

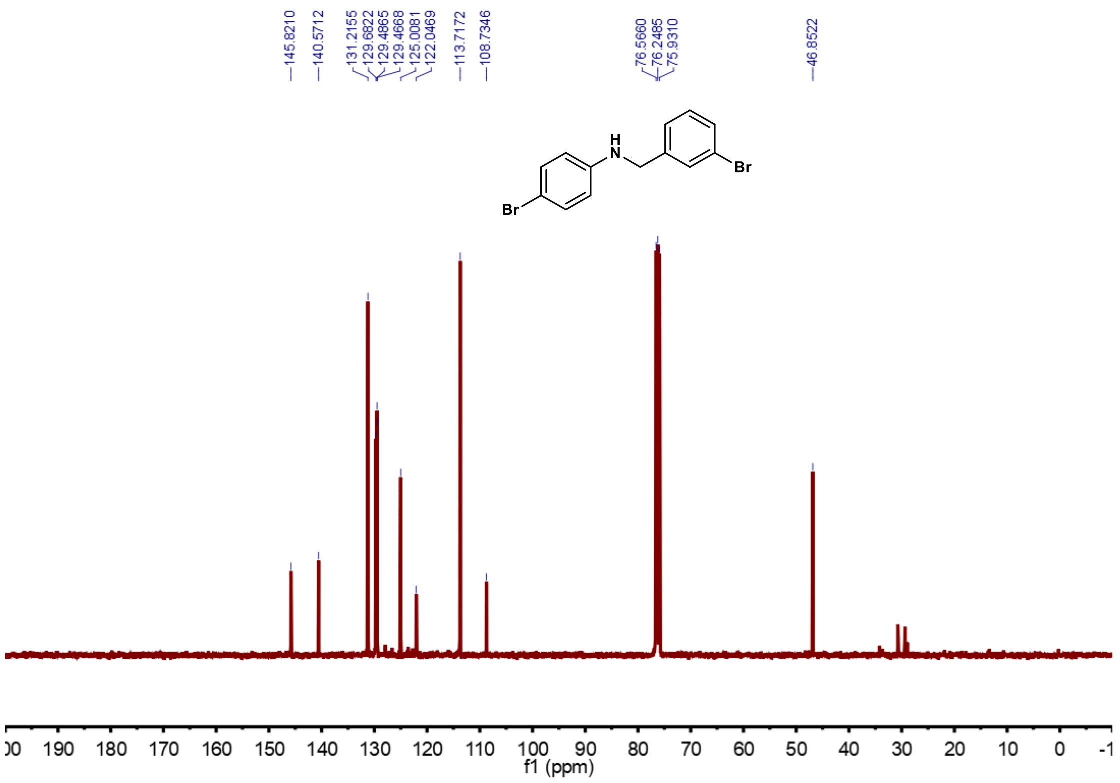
145.8210
140.5712
131.2155
129.6822
129.4865
129.4668
125.0081
122.0469
113.7172
108.7346
76.5660
76.2485
75.9310
46.8522
Br
H
N
Br
f1 (ppm)

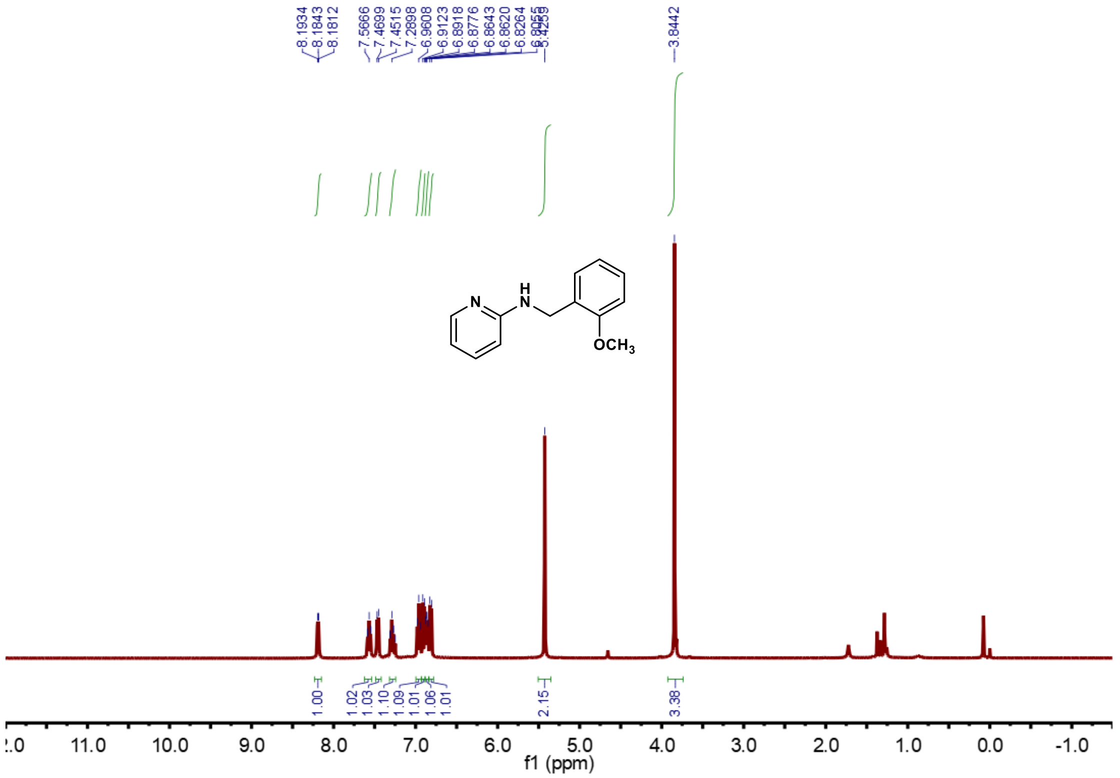
8.1934
8.1843
8.1812
7.5666
7.4699
7.4515
7.2898
6.9608
6.9123
6.8918
6.8776
6.8643
6.8620
6.8264
3.8442
N
H
N
OCH3
1.00
1.02
1.03
1.10
1.09
1.01
1.06
1.01
2.15
3.38
f1 (ppm)

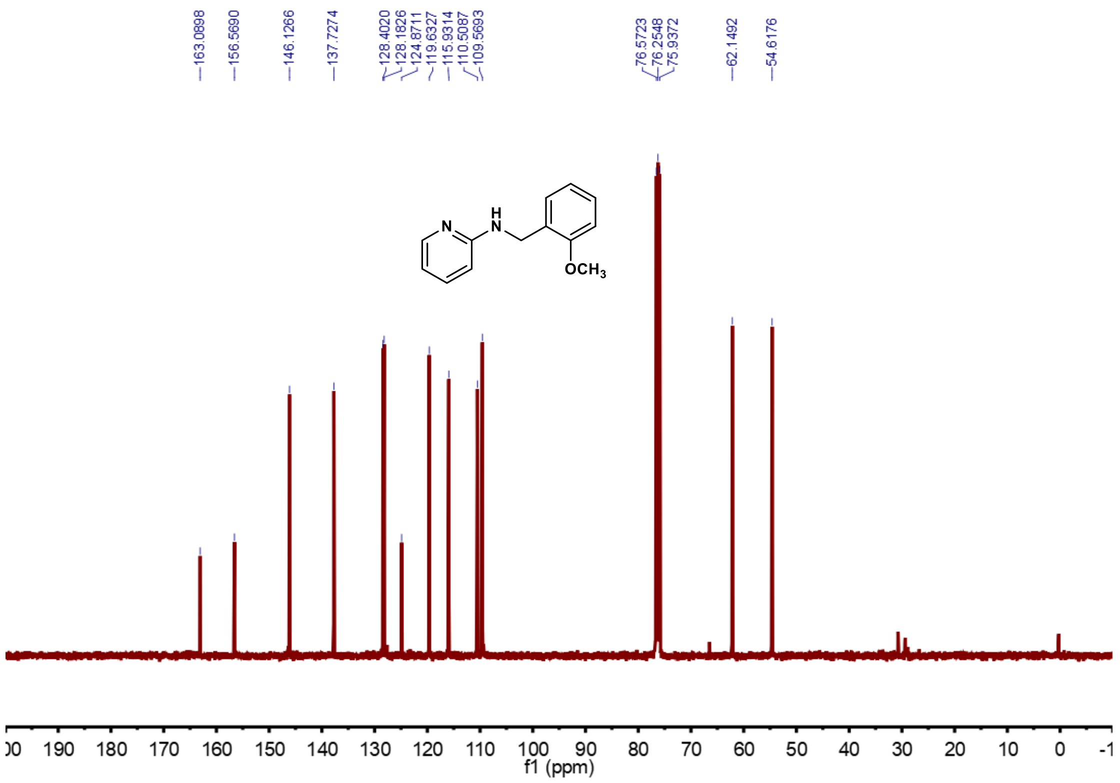
163.0898
156.5690
146.1266
137.7274
128.4020
128.1826
124.8711
119.6327
115.9314
110.5087
109.5693
76.5723
76.2548
75.9372
62.1492
54.6176
N
H
N
OCH3
f1 (ppm)

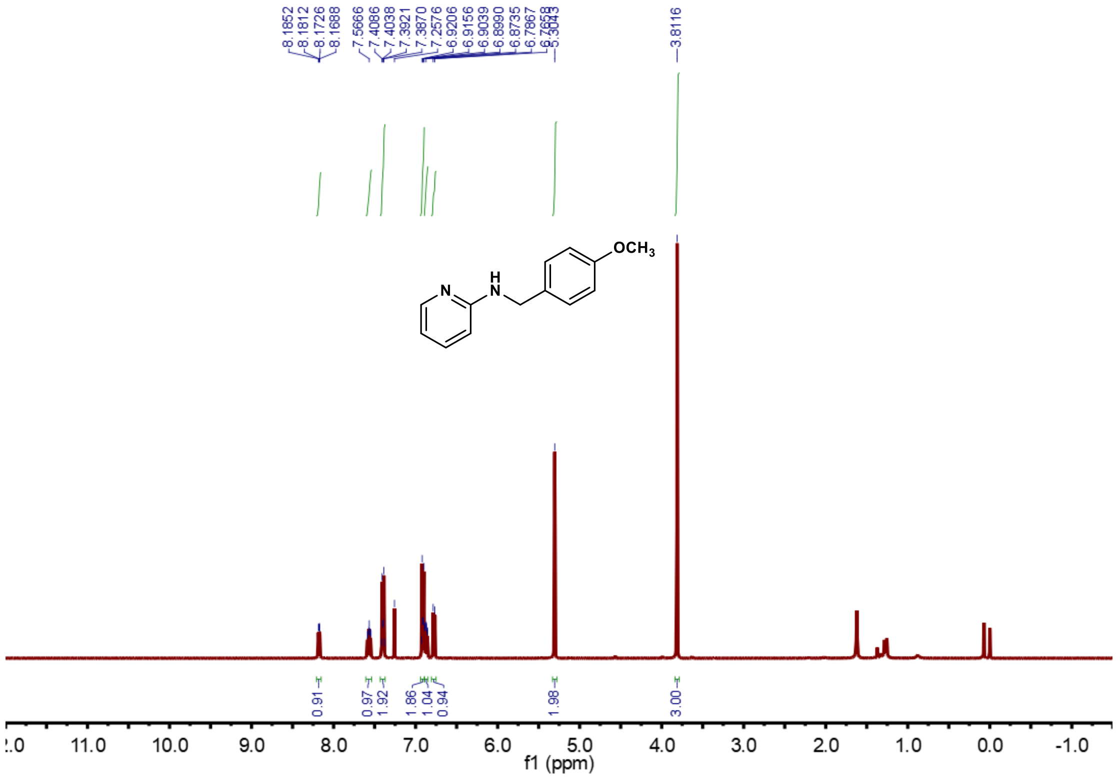
8.1852
8.1812
8.1726
8.1688
7.5666
7.4086
7.4038
7.3921
7.3870
7.2576
6.9206
6.9156
6.9039
6.8990
6.8735
6.7867
3.8116
N
H
N
OCH3
0.91
0.97
1.92
1.86
1.04
0.94
1.98
3.00
f1 (ppm)

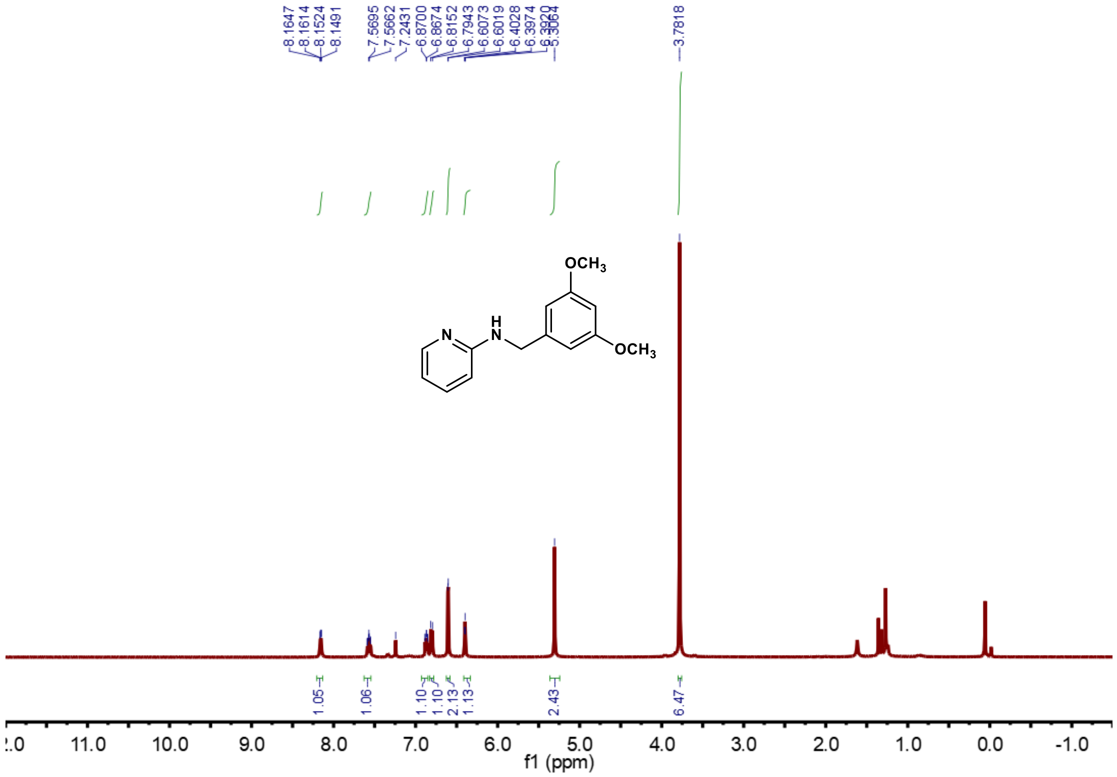
8.1647
8.1614
8.1524
8.1491
7.5695
7.5662
7.2431
6.8700
6.8674
6.8152
6.7943
6.6073
6.6019
6.4028
6.3974
3.7818
OCH3
OCH3
N
H
N
1.05
1.06
1.10
1.10
2.13
1.13
2.43
6.47
f1 (ppm)

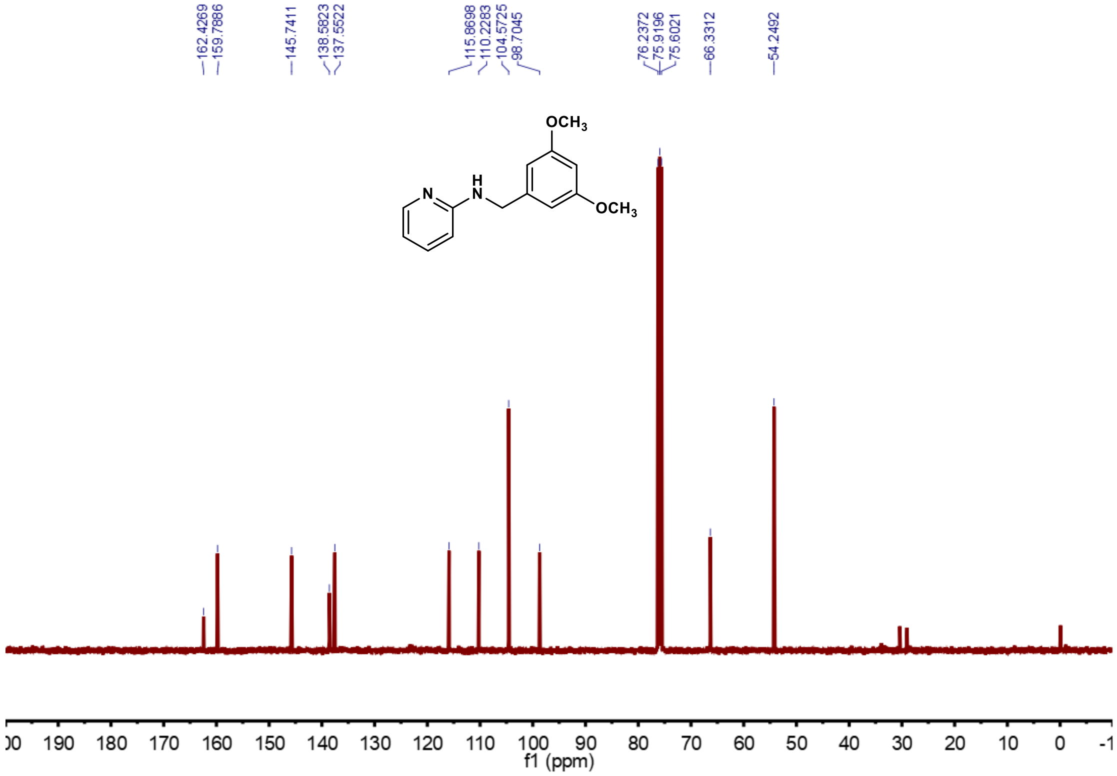
162.4269
159.7886
145.7411
138.5823
137.5522
115.8698
110.2283
104.5725
98.7045
76.2372
75.9196
75.6021
66.3312
54.2492
OCH3
OCH3
N
H
N
f1 (ppm)

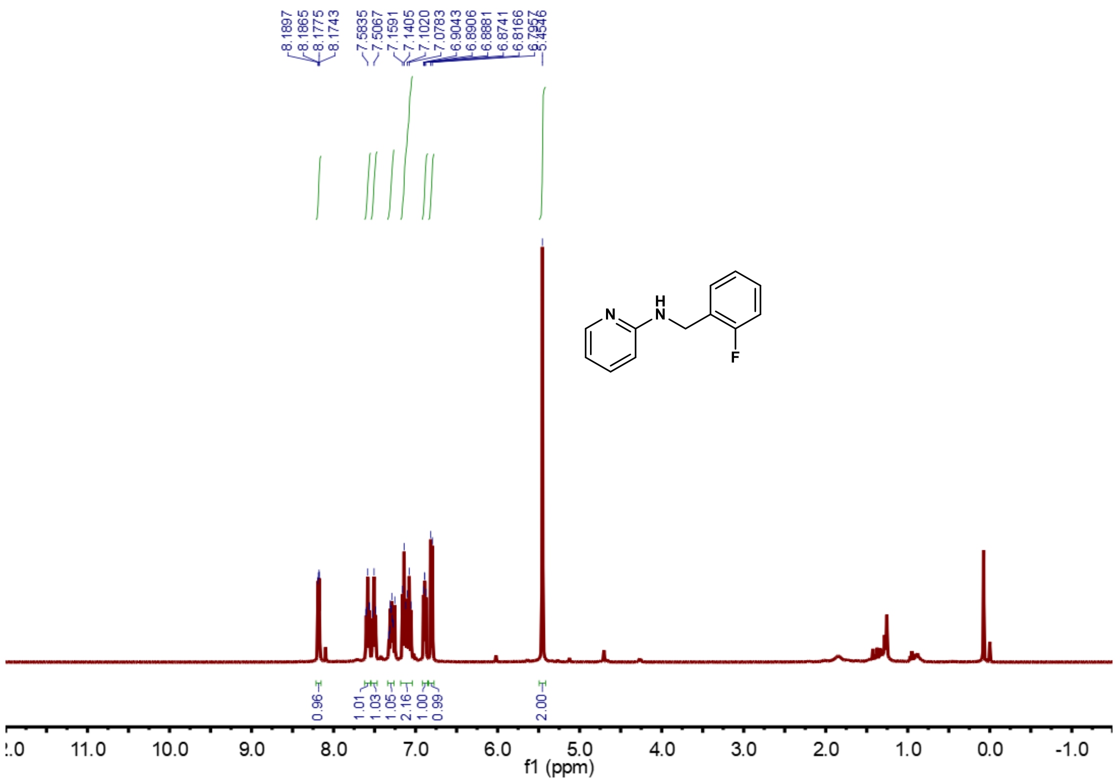
8.1897
8.1865
8.1775
8.1743
7.5835
7.5067
7.1591
7.1405
7.1020
7.0783
6.9043
6.8906
6.8881
6.8741
6.8166
5.7957
5.4546
N
H
N
F
0.96
1.01
1.03
1.05
2.16
1.00
0.99
2.00
12.0 11.0 10.0 9.0 8.0 7.0 6.0 5.0 4.0 3.0 2.0 1.0 0.0 -1.0
f1 (ppm)

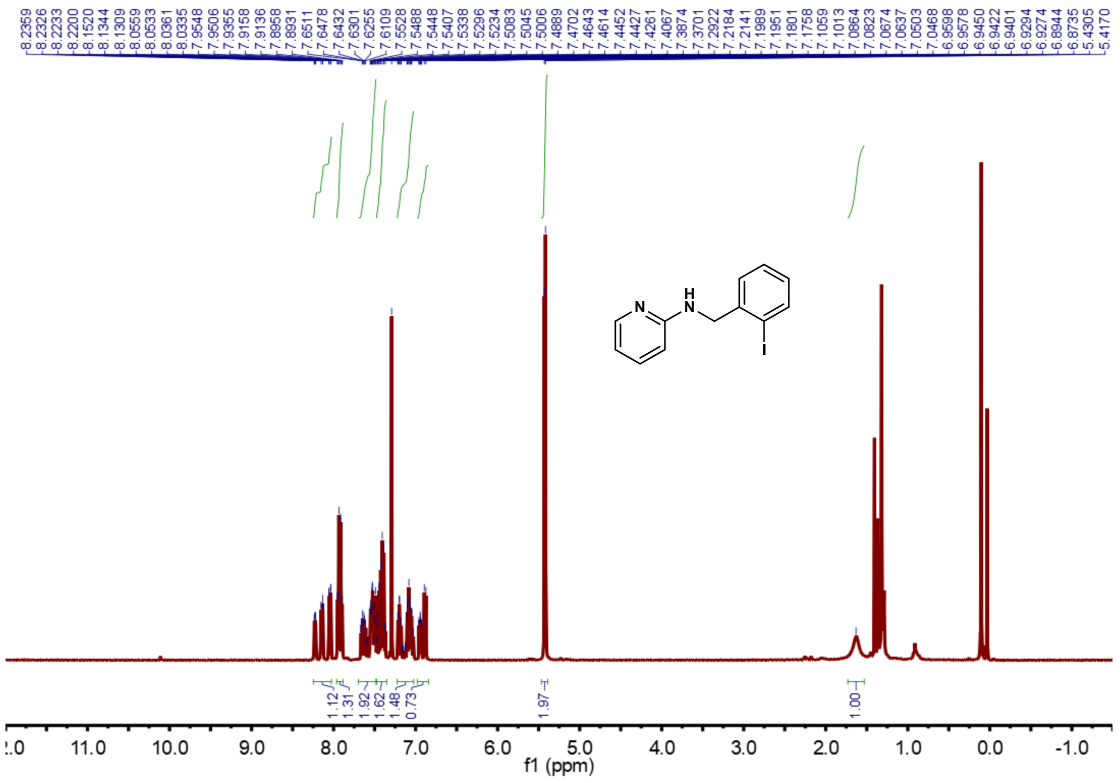
8.2359
8.2326
8.2233
8.2200
8.1520
8.1344
8.1309
8.0559
8.0533
8.0361
8.0335
7.9548
7.9506
7.9355
7.9158
7.9136
7.8958
7.8931
7.6511
7.6478
7.6432
7.6301
7.6255
7.6109
7.5528
7.5488
7.5407
7.5338
7.5296
7.5234
7.5083
7.5045
7.5006
7.4889
7.4702
7.4643
7.4614
7.4452
7.4427
7.4261
7.4067
7.3874
7.3701
7.2922
7.2184
7.2141
7.1989
7.1951
7.1801
7.1758
7.1059
7.1013
7.0864
7.0823
7.0674
7.0637
7.0503
7.0468
6.9598
6.9578
6.9450
6.9422
6.9401
6.9294
6.9274
6.8944
6.8735
5.4305
5.4170
N
H
N
I
1.12
1.31
1.92
1.62
1.48
0.73
1.97
1.00
12.0 11.0 10.0 9.0 8.0 7.0 6.0 5.0 4.0 3.0 2.0 1.0 0.0 -1.0
f1 (ppm)

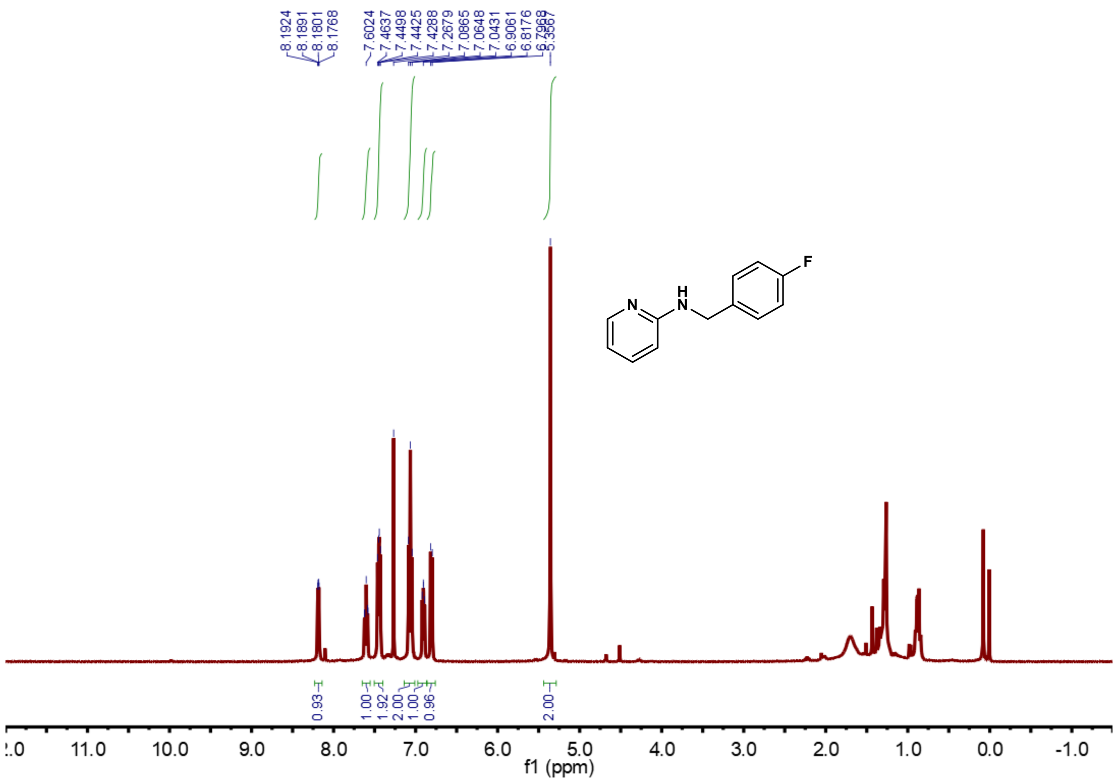
8.1924
8.1891
8.1801
8.1768
7.6024
7.4637
7.4498
7.4425
7.4288
7.2679
7.0865
7.0648
7.0431
6.9061
6.8176
5.3567
N
H
N
F
0.93
1.00
1.92
2.00
1.00
0.96
2.00
11.0
10.0
9.0
8.0
7.0
6.0
5.0
4.0
3.0
2.0
1.0
0.0
-1.0
f1 (ppm)

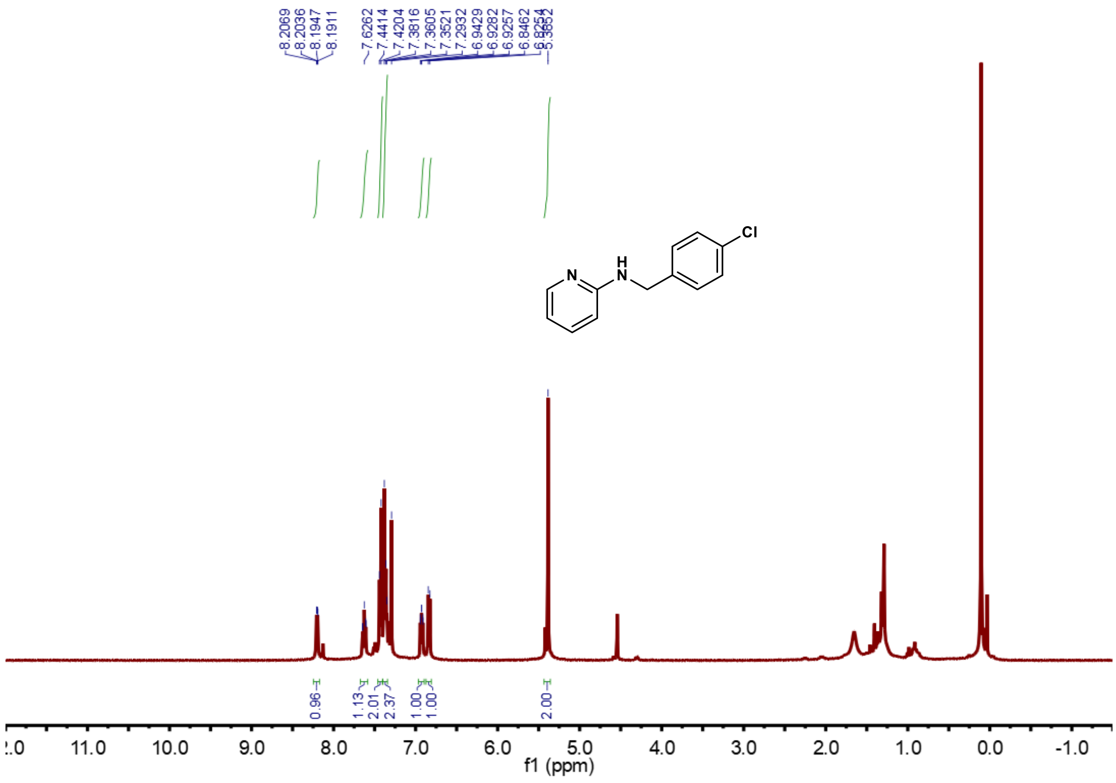
8.2069
8.2036
8.1947
8.1911
7.6262
7.4414
7.4204
7.3816
7.3605
7.3521
7.2932
6.9429
6.9282
6.9257
6.8462
5.3652
N
H
N
Cl
0.96
1.13
2.01
2.37
1.00
1.00
2.00
11.0
10.0
9.0
8.0
7.0
6.0
5.0
4.0
3.0
2.0
1.0
0.0
-1.0
f1 (ppm)

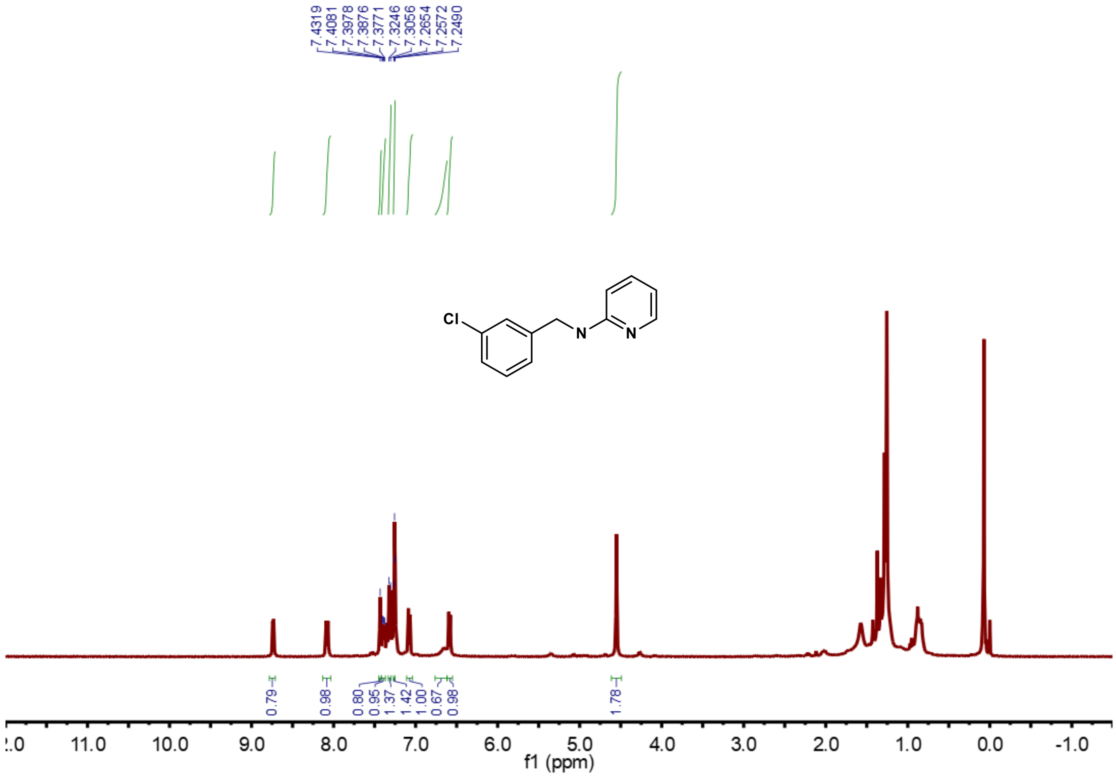
7.4319
7.4081
7.3978
7.3876
7.3771
7.3246
7.3056
7.2654
7.2572
7.2490
Cl
N
N
0.79
0.98
0.80
0.95
1.37
1.42
1.00
0.67
0.98
1.78
f1 (ppm)

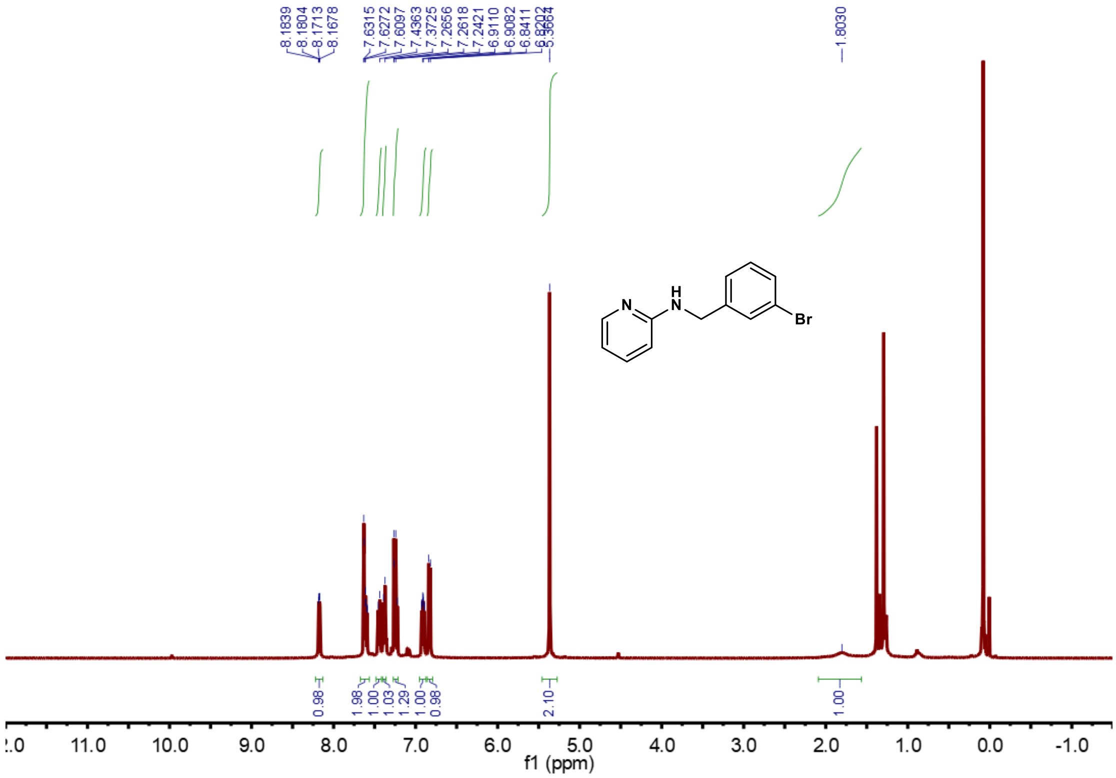
8.1839
8.1804
8.1713
8.1678
7.6315
7.6272
7.6097
7.4363
7.3725
7.2656
7.2618
7.2421
6.9110
6.9082
6.8411
1.8030
H
N
N
Br
0.98
1.98
1.00
1.03
1.29
1.00
0.98
2.10
1.00
f1 (ppm)

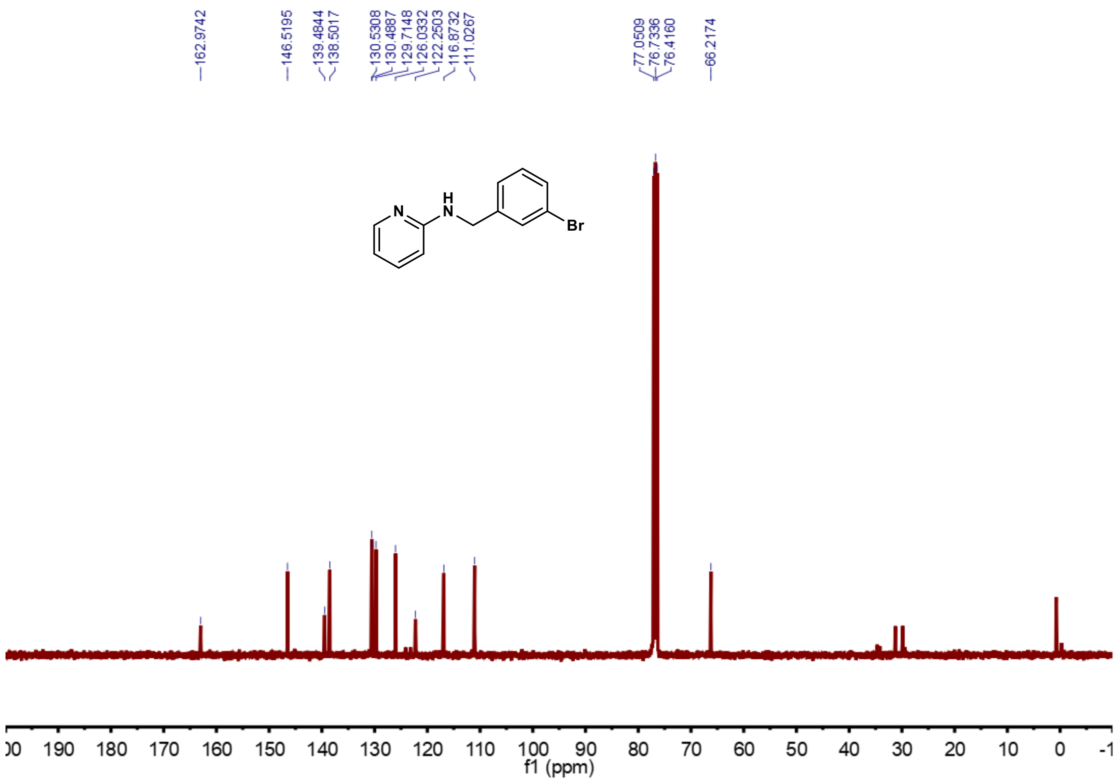

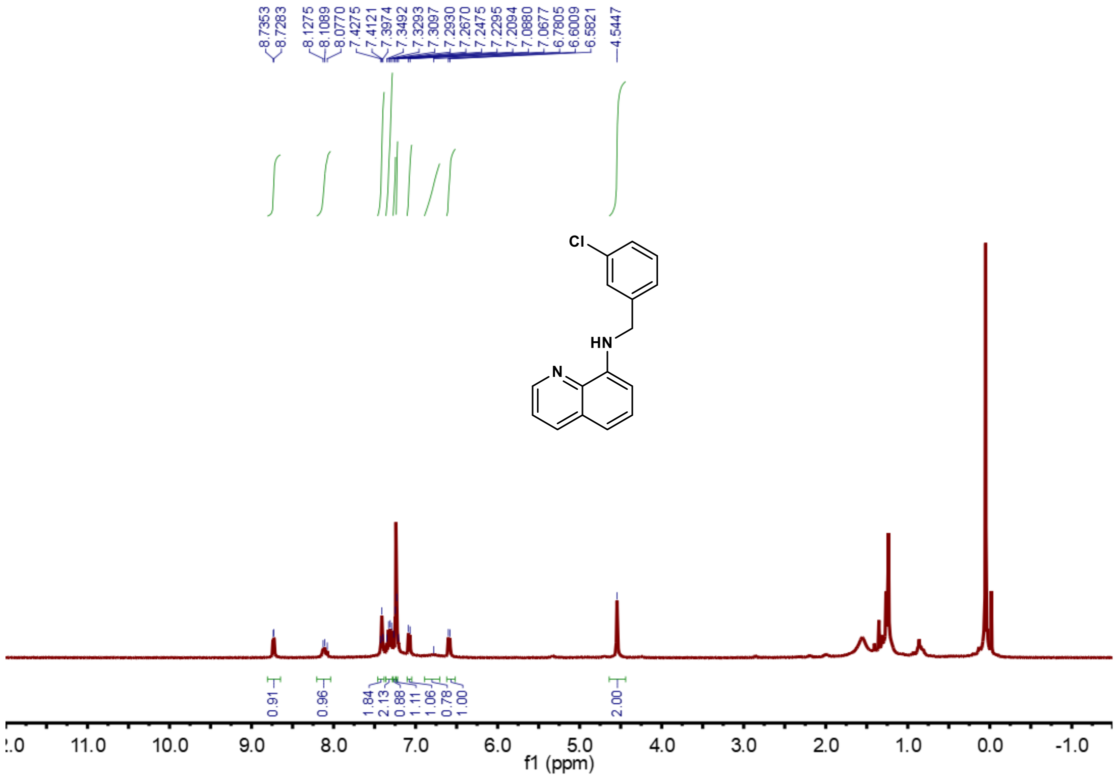

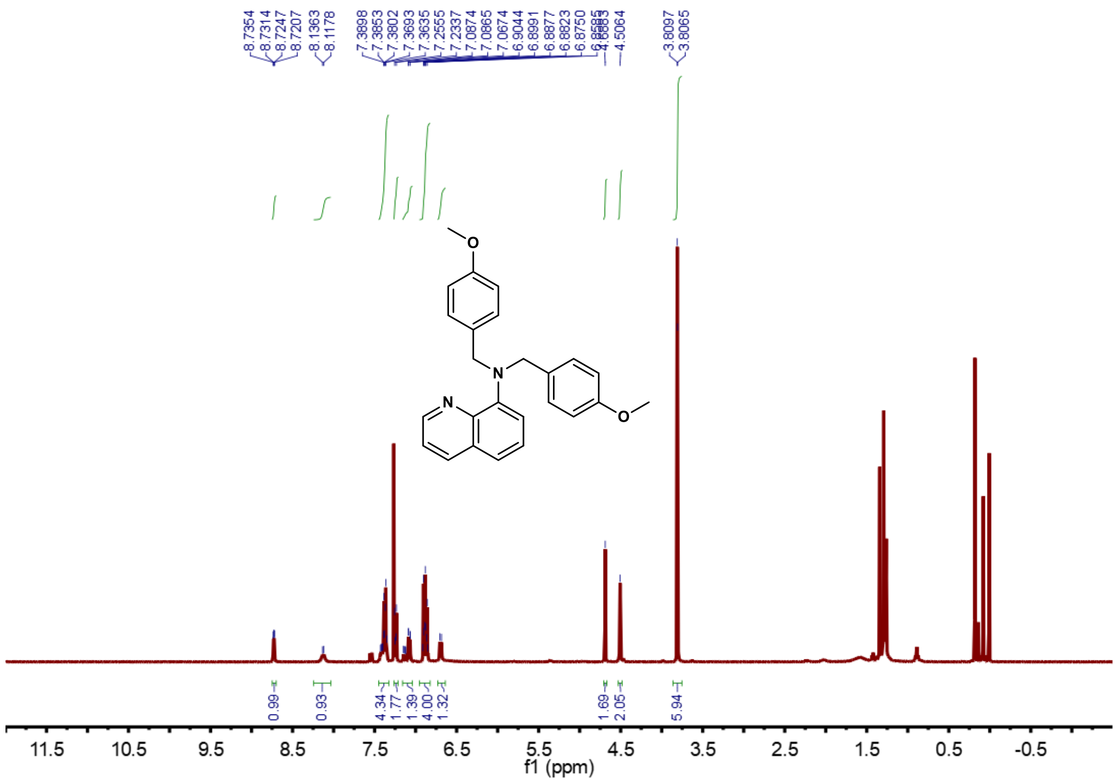

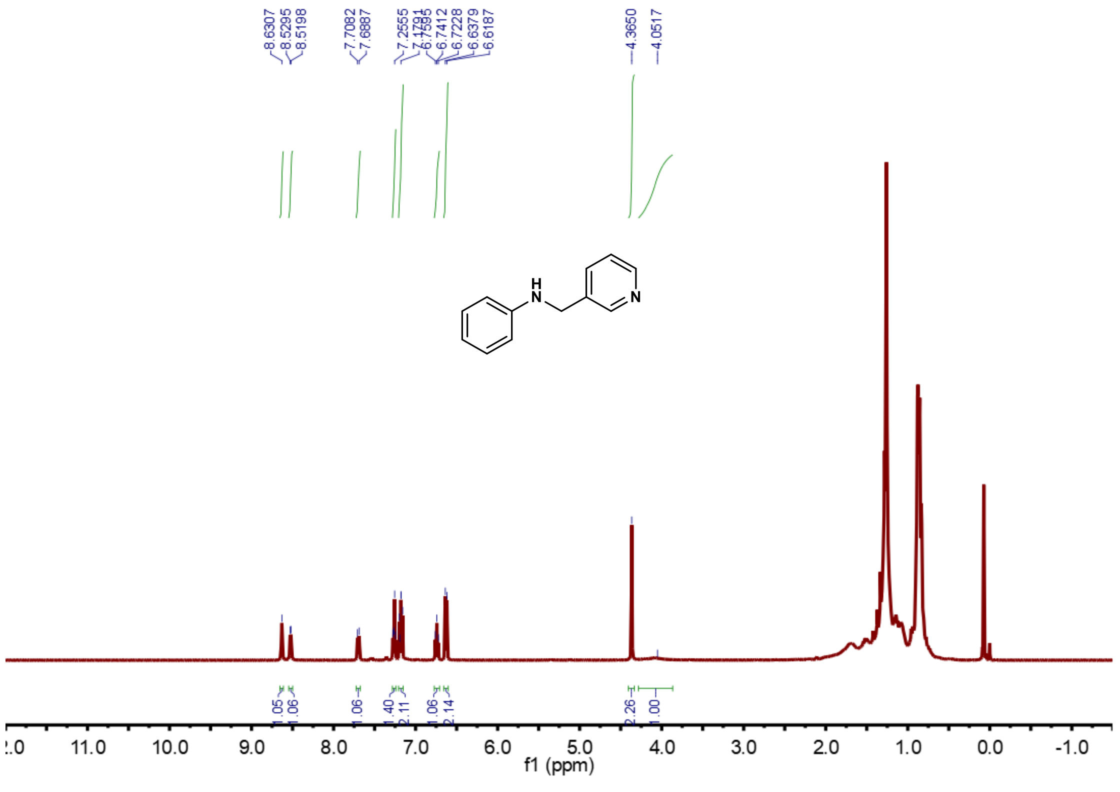

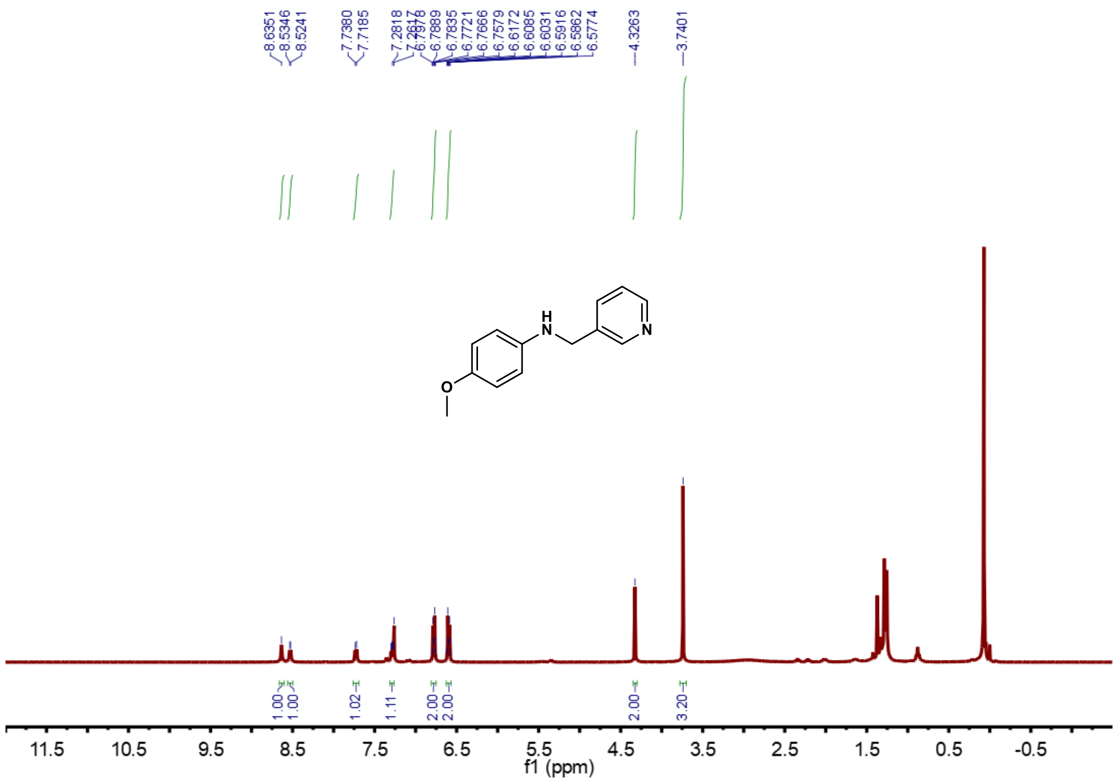

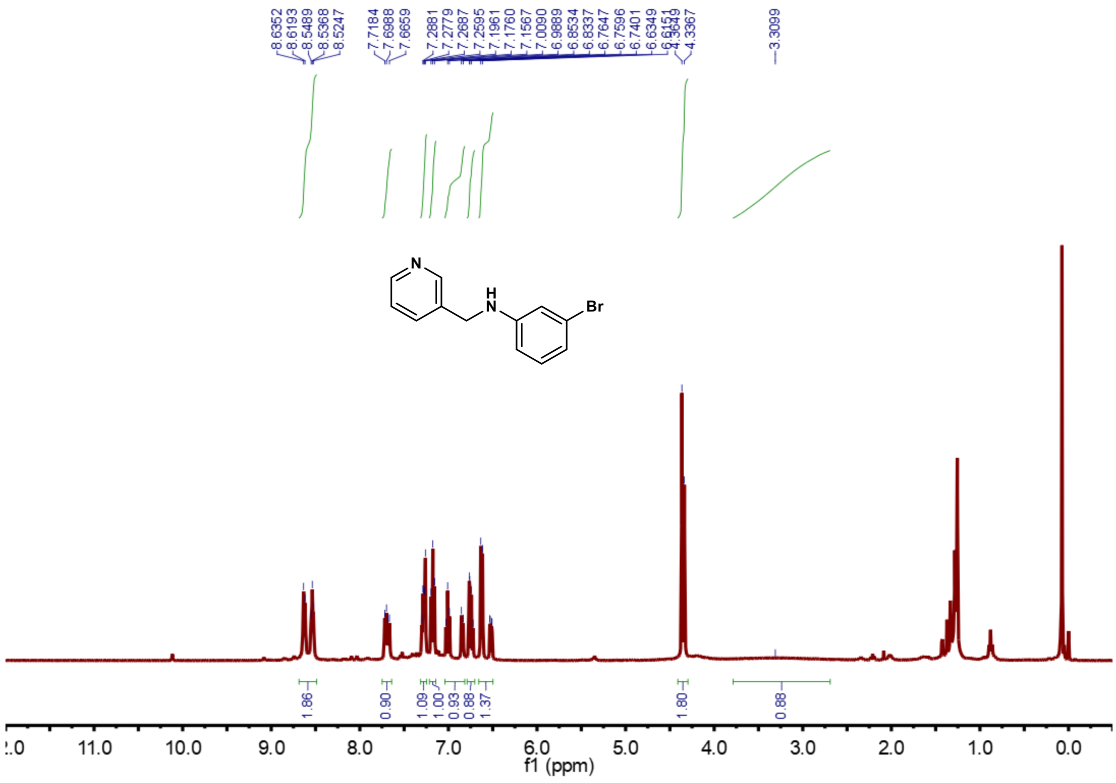

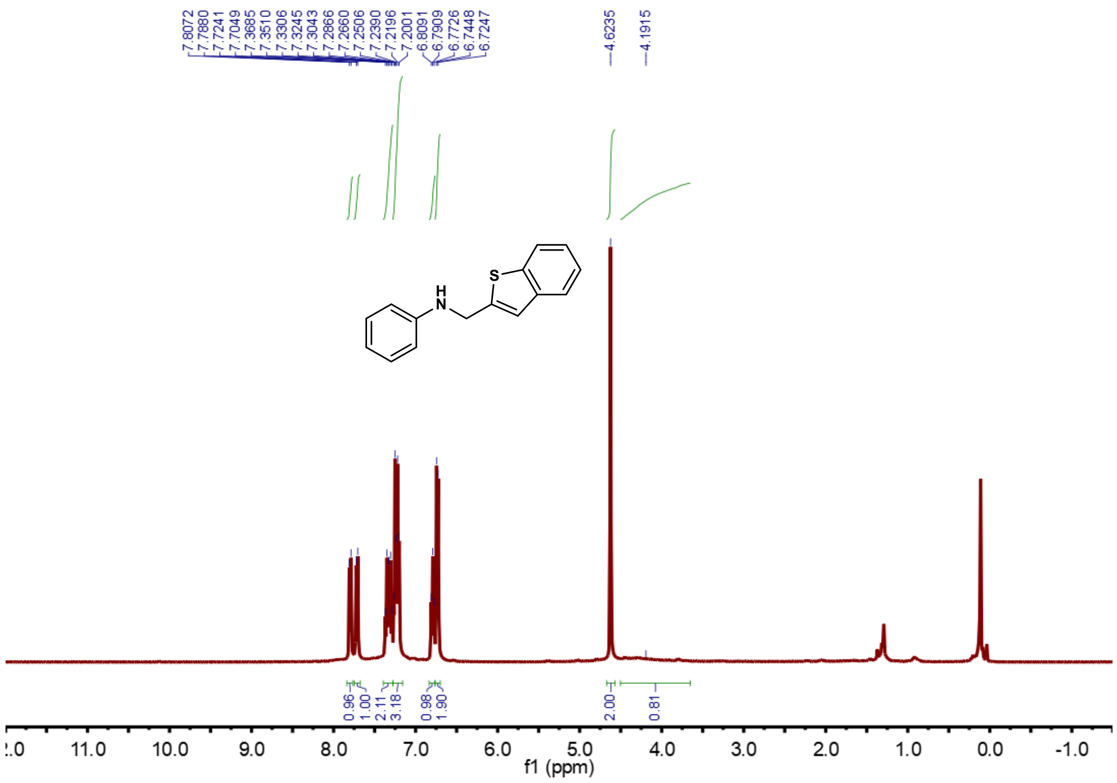
f1 (ppm)

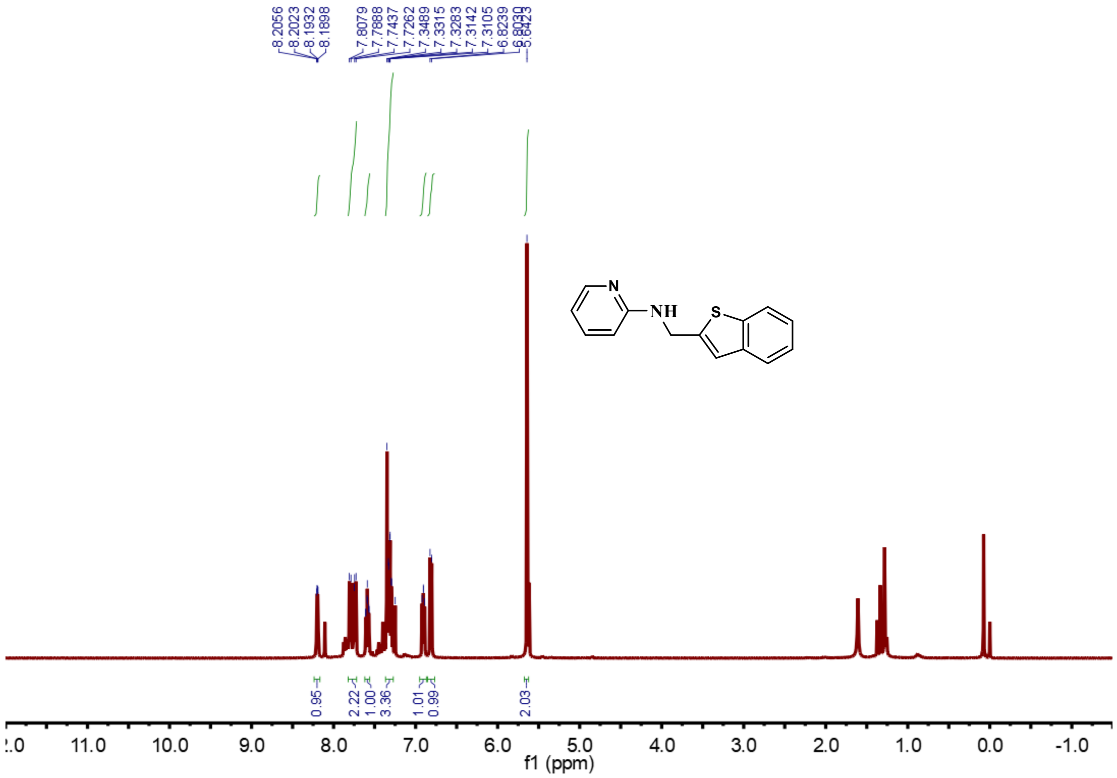
f1 (ppm)

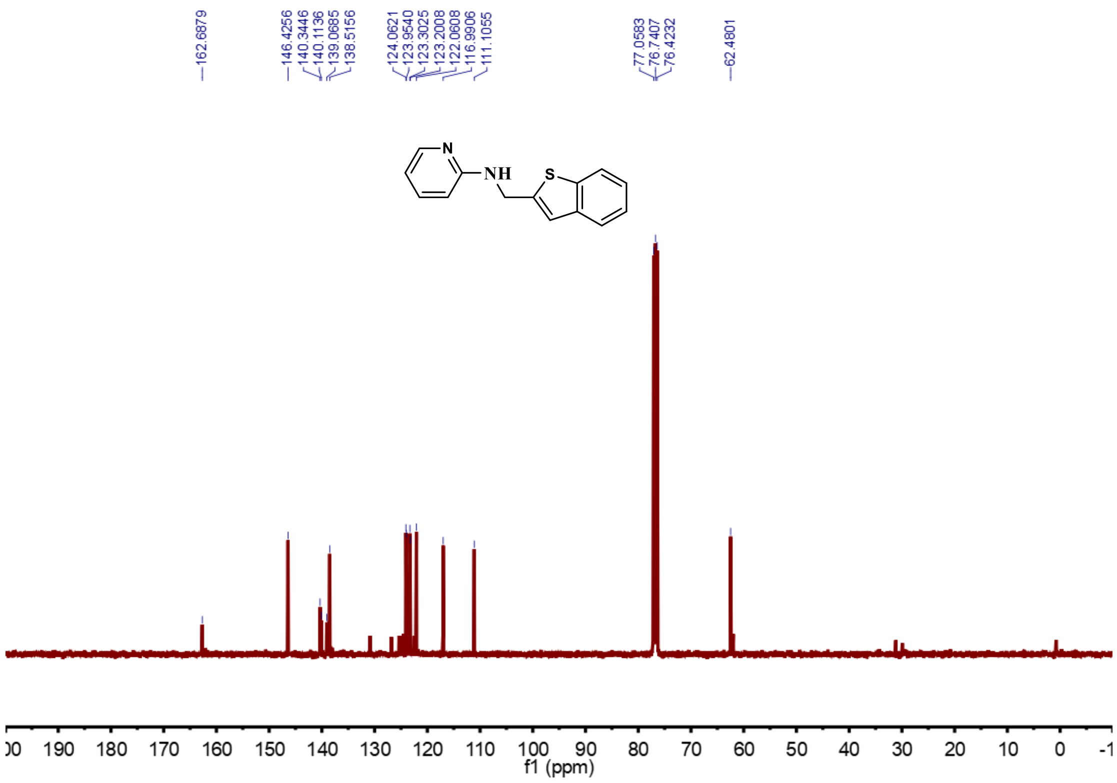

162.6879
146.4256
140.3446
140.1136
139.0685
138.5156
124.0621
123.9540
123.3025
123.2008
122.0608
116.9906
111.1055
77.0583
76.7407
76.4232
62.4801
N
NH
S
f1 (ppm)

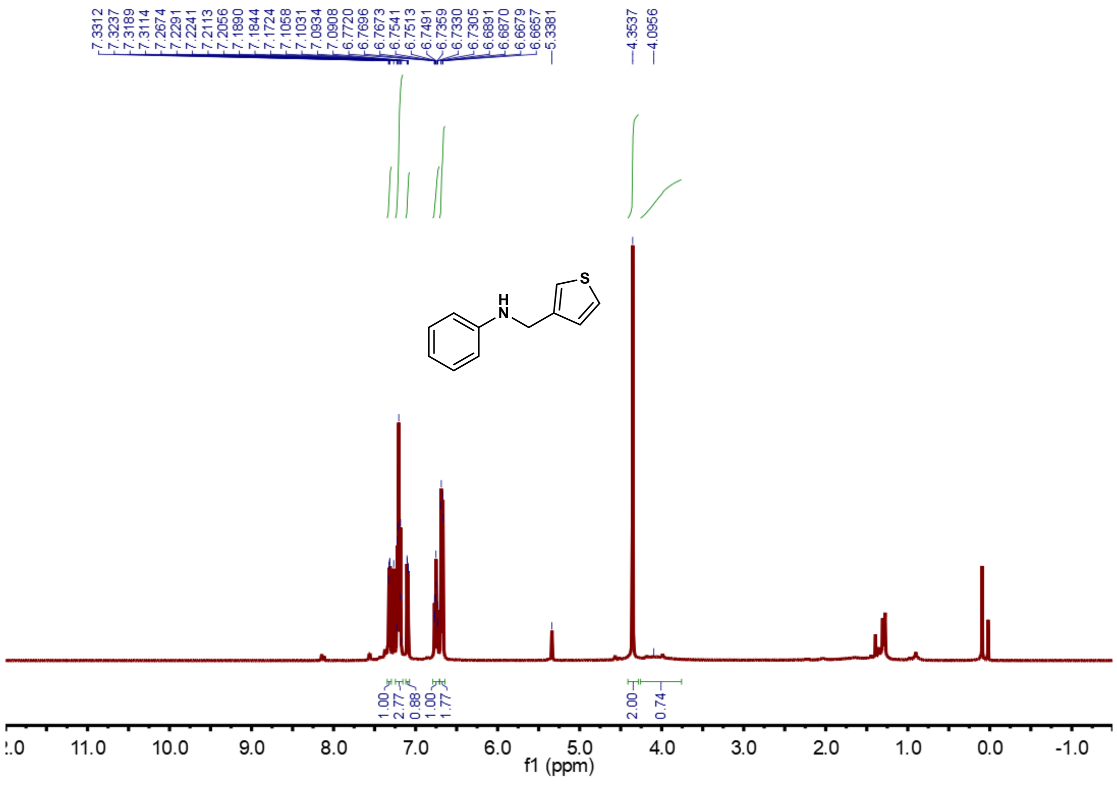

7.3312
7.3237
7.3189
7.3114
7.2674
7.2291
7.2241
7.2113
7.2056
7.1890
7.1844
7.1724
7.1058
7.1031
7.0934
7.0908
6.7720
6.7696
6.7673
6.7541
6.7513
6.7491
6.7359
6.7330
6.7305
6.6891
6.6870
6.6679
6.6657
5.3381
4.3537
4.0956
H
N
S
1.00
2.77
0.88
1.00
1.77
2.00
0.74
f1 (ppm)

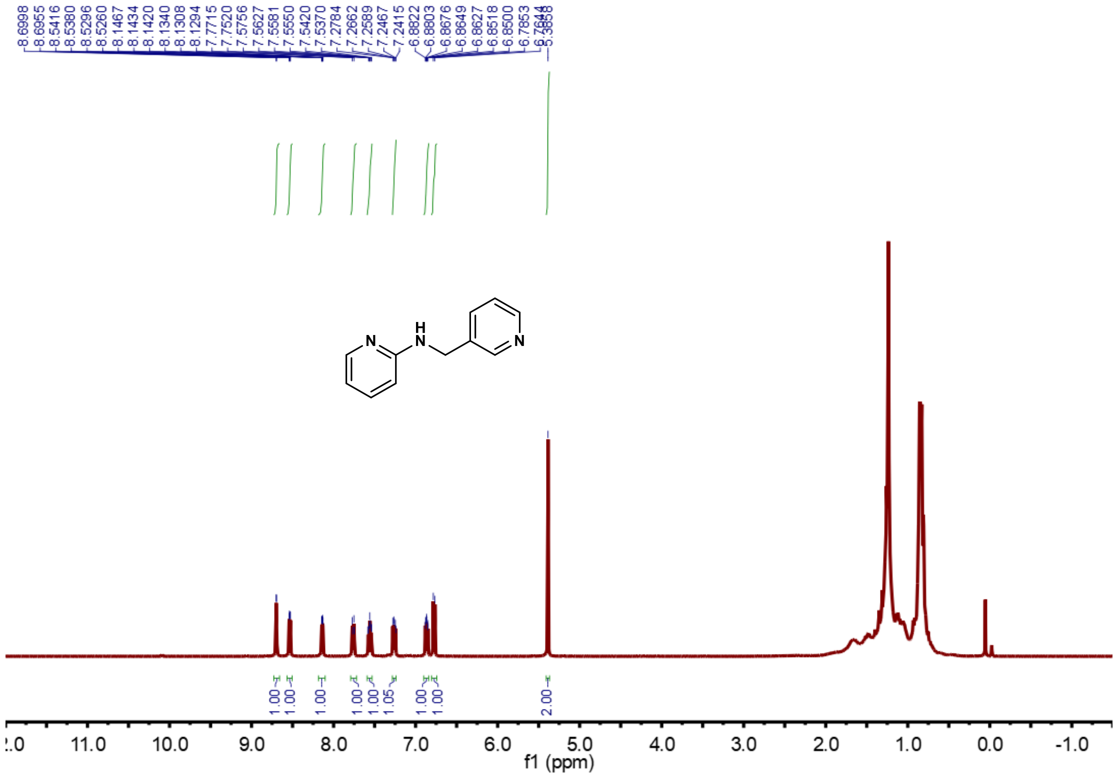

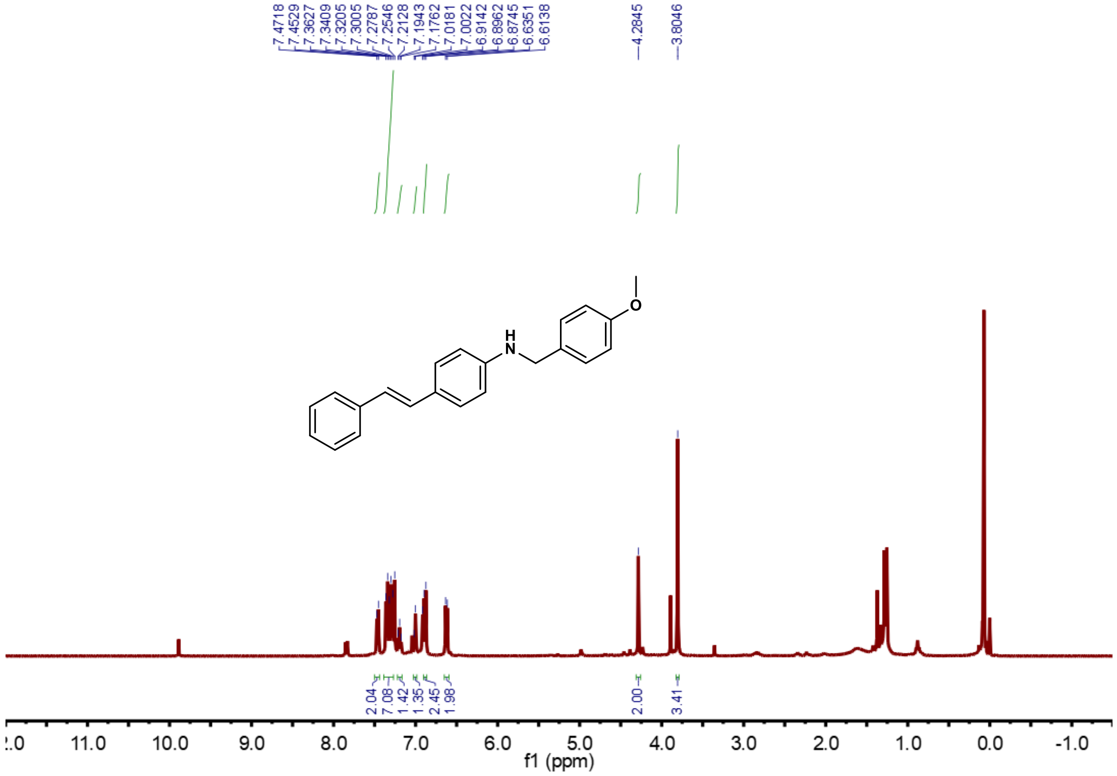

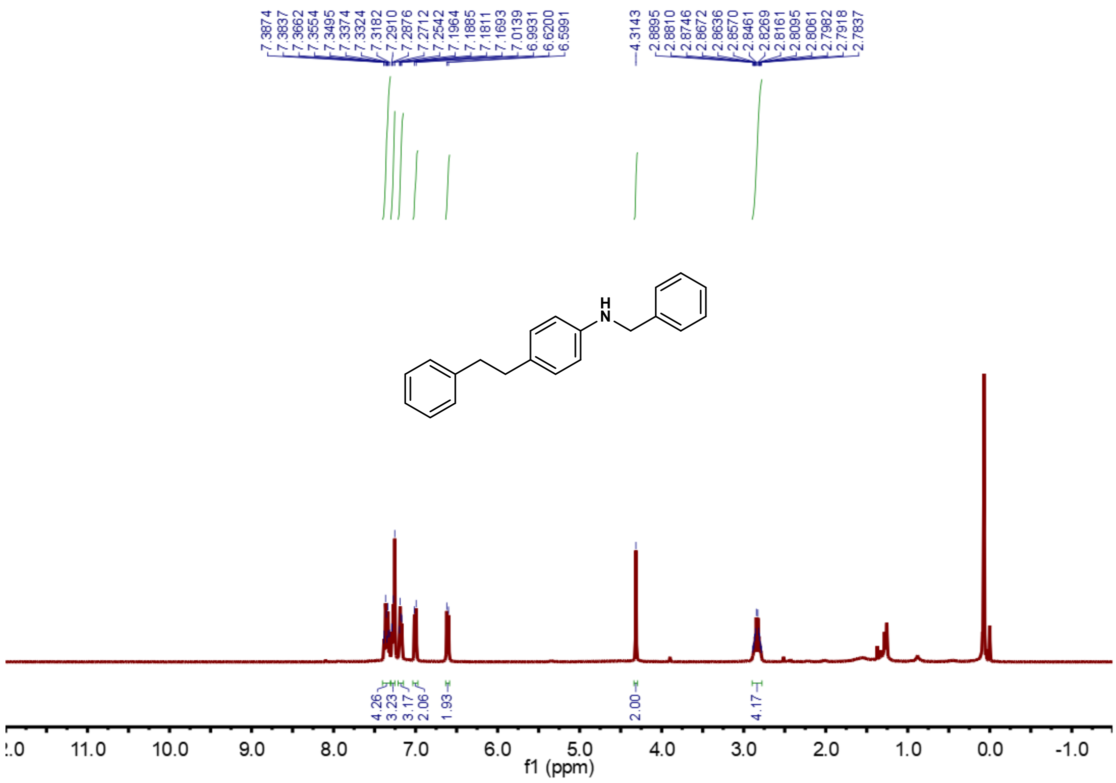
7.3874
7.3837
7.3662
7.3554
7.3495
7.3374
7.3324
7.3182
7.2910
7.2876
7.2712
7.2542
7.1964
7.1885
7.1811
7.1693
7.0139
6.9931
6.6200
6.5991
4.3143
2.8895
2.8810
2.8746
2.8672
2.8636
2.8570
2.8461
2.8269
2.8161
2.8095
2.8061
2.7982
2.7918
2.7837
H
N
4.26
3.23
3.17
2.06
1.93
2.00
4.17
f1 (ppm)

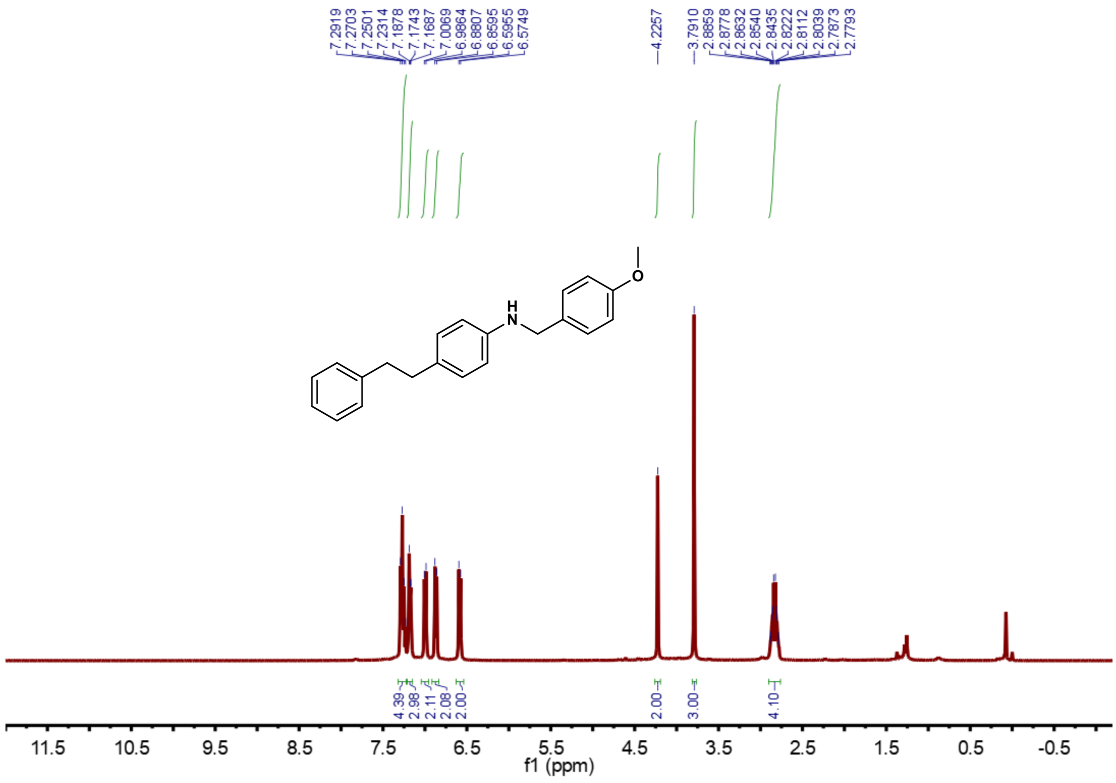
7.2919
7.2703
7.2501
7.2314
7.1878
7.1743
7.1687
7.0069
6.9864
6.8807
6.8595
6.5955
6.5749
4.2257
3.7910
2.8859
2.8778
2.8632
2.8540
2.8435
2.8222
2.8112
2.8039
2.7873
2.7793
H
N
O
4.39
2.98
2.11
2.08
2.00
2.00
3.00
4.10
f1 (ppm)

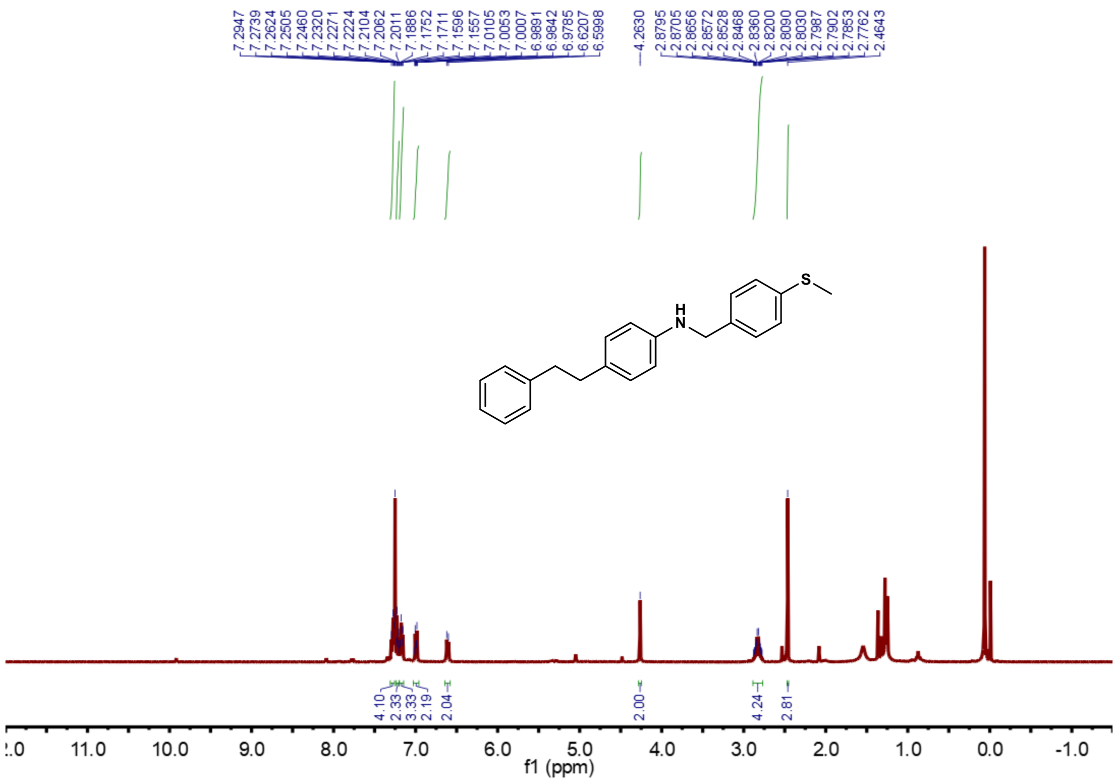
7.2947
7.2739
7.2624
7.2505
7.2460
7.2320
7.2271
7.2224
7.2104
7.2062
7.2011
7.1886
7.1752
7.1711
7.1596
7.1557
7.0105
7.0053
7.0007
6.9891
6.9842
6.9785
6.6207
6.5998
4.2630
2.8795
2.8705
2.8656
2.8572
2.8528
2.8468
2.8360
2.8200
2.8090
2.8030
2.7987
2.7902
2.7853
2.7762
2.4643
H
N
S
4.10
2.33
3.33
2.19
2.04
2.00
4.24
2.81
11.0
10.0
9.0
8.0
7.0
6.0
5.0
4.0
3.0
2.0
1.0
0.0
-1.0
f1 (ppm)

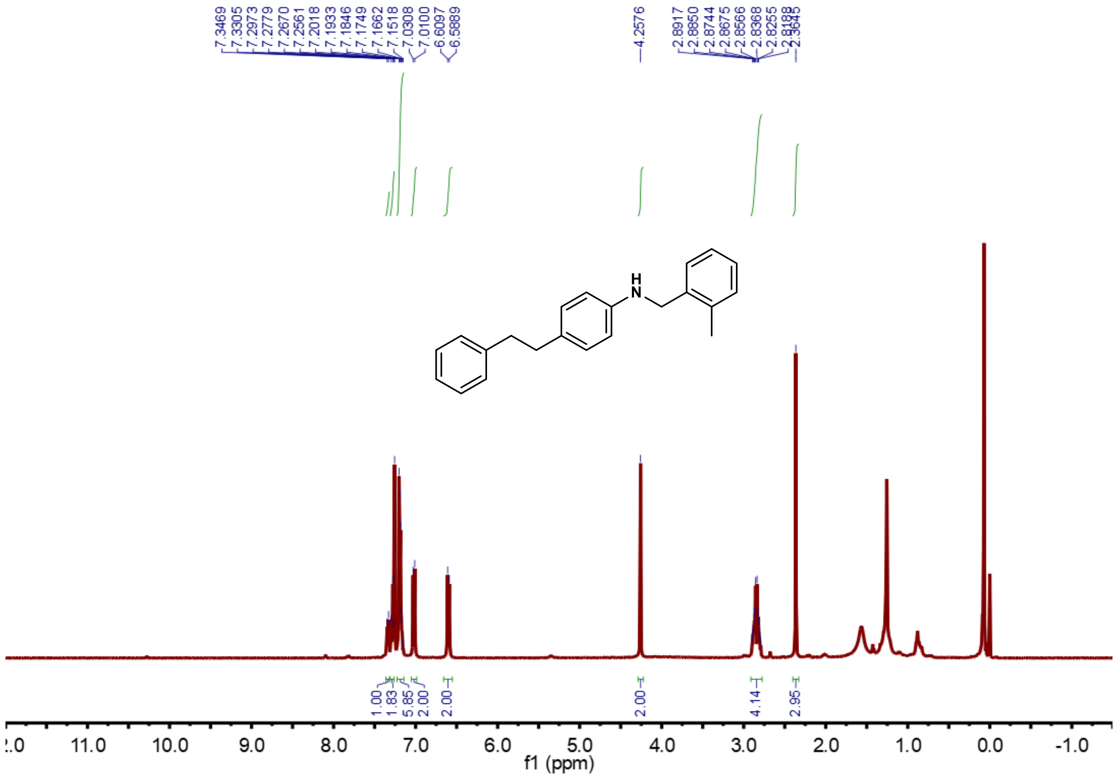
7.3469
7.3305
7.2973
7.2779
7.2670
7.2561
7.2018
7.1933
7.1846
7.1749
7.1662
7.1518
7.0308
7.0100
6.6097
6.5889
4.2576
2.8917
2.8850
2.8744
2.8675
2.8566
2.8368
2.8255
2.3645
H
N
1.00
1.83
5.85
2.00
2.00
2.00
4.14
2.95
11.0
10.0
9.0
8.0
7.0
6.0
5.0
4.0
3.0
2.0
1.0
0.0
-1.0
f1 (ppm)

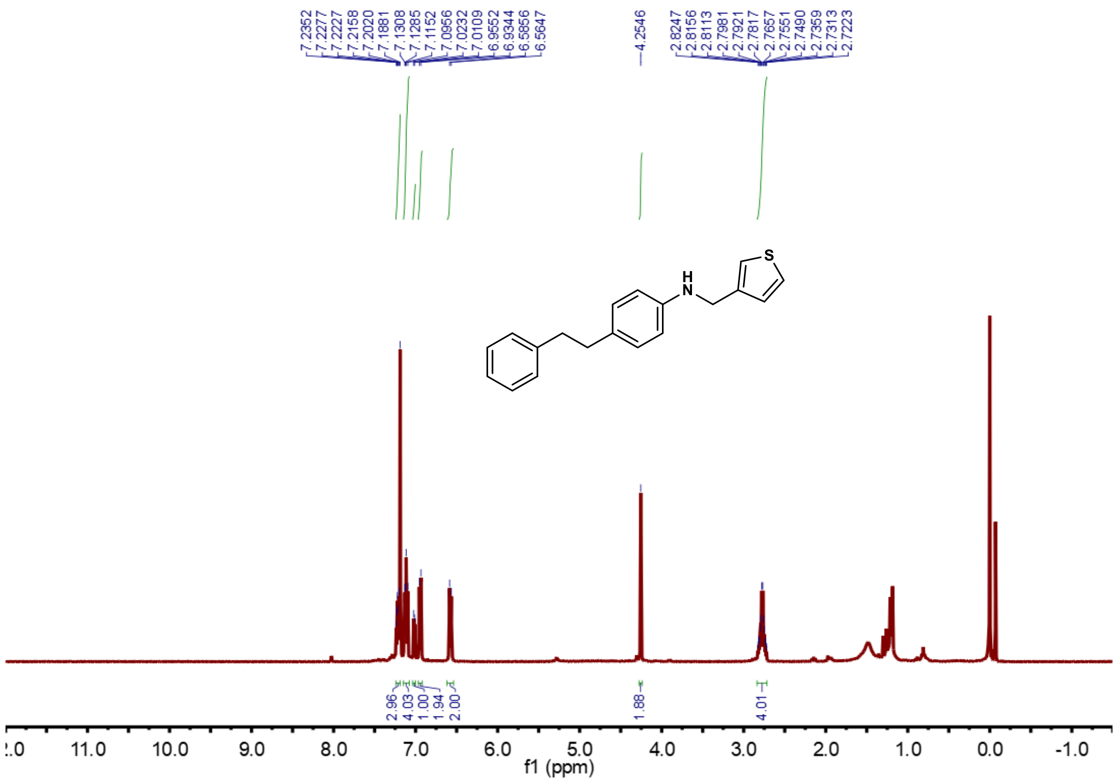
7.2352
7.2277
7.2227
7.2158
7.2020
7.1881
7.1308
7.1285
7.1152
7.0956
7.0232
7.0109
6.9552
6.9344
6.5856
6.5647
4.2546
2.8247
2.8156
2.8113
2.7981
2.7921
2.7817
2.7657
2.7551
2.7490
2.7359
2.7313
2.7223
S
H
N
2.96
4.03
1.00
1.94
2.00
1.88
4.01
11.0
10.0
9.0
8.0
7.0
6.0
5.0
4.0
3.0
2.0
1.0
0.0
-1.0
f1 (ppm)

S5. Supplementary References

1. Ma, X., Su, C. & Xu, Q. N-alkylation by hydrogen autotransfer reactions. *Top. Curr. Chem.* **374,** 27 (2016).
2. Liu, H. et al. Atomic-level asymmetric tuning of the $Co_1–N_3P_1$ catalyst for highly efficient N-alkylation of amines with alcohols. *J. Am. Chem. Soc.* **146**, 20518-20529 (2024).
3. Wang, L., Neumann, H. & Beller, M. Palladium-catalyzed methylation of nitroarenes with methanol. *Angew. Chem. Int. Ed.* **58**, 5417-5421 (2019).
4. González-Lainez, M., Jiménez, M. V., Passarelli, V. & Pérez-Torrente, J. J. Effective N-methylation of nitroarenes with methanol catalyzed by a functionalized NHC-based iridium catalyst: a green approach to N-methyl amines. *Catal. Sci. Technol.* **10**, 3458-3467 (2020).
5. Salarinejad, N., Dabiri, M. & Movahed, S. K. A novel core@double shell magnetic nitrogen-doped carbon nanotubes as a support for palladium nanoparticles: a highly efficient magnetic catalyst in the direct reductive coupling of nitroarenes and alcohols. *Catal. Commun.* **172,** 106529 (2022).
6. Chen, J., Huang, S., Lin, J. & Su, W. Recyclable palladium catalyst for facile synthesis of imines from benzyl alcohols and nitroarenes. *Appl. Catal. Gen.* **470,** 1-7 (2014).
7. Song, T., Duan, Y., Chen, X. & Yang, Y. Switchable access to amines and imines from reductive coupling of nitroarenes with alcohols catalyzed by biomass-derived cobalt nanoparticles. *Catalysts* **9,** 116 (2019).
8. Tang, C.-H. et al. Direct one-pot reductive N-alkylation of nitroarenes by using alcohols with supported gold catalysts. *Chem. Eur. J.* **17,** 7172-7177 (2011).
9. Cui, X., Zhang, C., Shi, F. & Deng, Y. Au/Ag–Mo nano-rods catalyzed reductive coupling of nitrobenzenes and alcohols using glycerol as the hydrogen source. *Chem. Commun.* **48,** 9391-9393 (2012).
10. Liu, D. et al. Direct reductive coupling of nitroarenes and alcohols catalysed by Co–N–C/CNT@AC. *Green Chem.* **21,** 2129-2137 (2019).
11. Mandi, U. et al. Mesoporous polyacrylic acid supported silver nanoparticles as an efficient catalyst for reductive coupling of nitrobenzenes and alcohols using glycerol as hydrogen source. *J. Colloid Interface Sci.* **472,** 202-209 (2016).
12. Liu, H., Khuan Chuah, G. & Jaenicke, S. Alumina-entrapped Ag catalyzed nitro compounds coupled with alcohols using borrowing hydrogen methodology. *Phys. Chem. Chem. Phys.* **17,** 15012-15018 (2015).
13. Peng, Q., Zhang, Y., Shi, F. & Deng, Y. Fe_2O_3-supported nano-gold catalyzed one-pot synthesis of N-alkylated anilines from nitroarenes and alcohols. *Chem. Commun.* **47,** 6476-6478 (2011).
14. Zhang, H., Wang, J., Liu, M. & Liu, D. Cu/Al_2O_3 catalyst prepared by a double hydrolysis method for a green, continuous and controlled N-methylation reaction of nitroarenes with methanol. *Appl. Surf. Sci.* **526,** 146708 (2020).
15. Jamil, M. A. R. et al. N-Methylation of amines and nitroarenes with methanol using heterogeneous platinum catalysts. *J. Catal.* **371,** 47-56 (2019).
16. Goyal, V. et al. Commercial Pd/C-catalyzed N-methylation of nitroarenes and amines using methanol as both C1 and H_2 source. *J. Org. Chem.* **84,** 15389-15398 (2019).
17. Tao, Y. et al. Highly active Ni nanoparticles on N-doped mesoporous carbon with tunable

selectivity for the one-pot transfer hydroalkylation of nitroarenes with EtOH in the absence of H_2. *ChemCatChem* **13,** 4243-4250 (2021).

18. Fu, A., Liu, Q., Jiang, M. & Xu, G. Selective N-monomethylation of amines and nitroarenes using methanol over an encapsulated iridium nanocatalyst. *Asian J. Org. Chem.* **8,** 487-491 (2019).
19. Chen, Y. et al. Highly efficient MgF_2 supported Co catalyst for cyclization reaction of o-nitroaniline and benzyl-alcohol. *Mol. Catal.* **545,** 113186 (2023).
20. Sankar, M. et al. Supported bimetallic nano-alloys as highly active catalysts for the one-pot tandem synthesis of imines and secondary amines from nitrobenzene and alcohols. *Catal. Sci. Technol.* **6,** 5473-5482 (2016).
21. Selvam, K., Sakamoto, H., Shiraishi, Y. & Hirai, T. One-pot synthesis of secondary amines from alcohols and nitroarenes on TiO_2 loaded with Pd nanoparticles under UV irradiation. *New J. Chem.* **39,** 2467-2473 (2015).
22. Song, Y. et al. One-pot synthesis of secondary amine via photoalkylation of nitroarenes with benzyl alcohol over Pd/monolayer $H_{1.07}Ti_{1.73}O_4{\cdot}H_2O$ nanosheets. *J. Catal.* **361,** 105-115 (2018).
23. Wang, J., Jiang, J. & Li, Z. Efficient one-pot syntheses of secondary amines from nitro aromatics and benzyl alcohols over Pd/NiTi-LDH under visible light. *Dalton Trans.* **52,** 16935-16942 (2023).
24. Hao, M. & Li, Z. Efficient visible light initiated one-pot syntheses of secondary amines from nitro aromatics and benzyl alcohols over Pd@NH_2-UiO-66(Zr). *Appl. Catal. B Environ.* **305,** 121031 (2022).
25. Jiang, H., Sheng, M., Li, Y., Kong, S. & Bian, F. Photocatalytic one-pot multidirectional N-alkylation over Pt/D-TiO_2/Ti_3C_2: Ti_3C_2-based short-range directional charge transmission. *Appl. Organomet. Chem.* **35,** e6291 (2021).
26. Zhang, L., Zhang, Y., Deng, Y. & Shi, F. Light-promoted N, N-dimethylation of amine and nitro compound with methanol catalyzed by Pd/TiO_2 at room temperature. *RSC Adv.* **5,** 14514-14521 (2015).
27. Delley, B. From molecules to solids with the DMol3 approach. *J. Chem. Phys.* **113,** 7756-7764 (2000).
28. Perdew, J. P., Burke, K. & Ernzerhof, M. Generalized gradient approximation made simple. *Phys. Rev. Lett.* **77,** 3865-3868 (1996).
29. Delley, B. Hardness conserving semilocal pseudopotentials. *Phys. Rev. B.* **66,** 155125 (2002).
30. Govind, N., Petersen, M., Fitzgerald, G., King-Smith, D. & Andzelm, J. A generalized synchronous transit method for transition state location. *Comput. Mater. Sci.* **28**, 250-258 (2003).
31. Kresse, G. & Hafner, J. Ab initio molecular dynamics for liquid metals. *Phys. Rev. B.* **47,** 558-561 (1993).
32. Kresse, G. & Furthmüller, J. Efficiency of ab-initio total energy calculations for metals and semiconductors using a plane-wave basis set. *Comput. Mater. Sci*. **6,** 15-50 (1996).
33. Blochl, P., Blöchl, E. & Blöchl, P. E. Projected augmented-wave method. *Phys. Rev. B Condens. Matter* **50,** 17953-17979 (1994).
34. Kresse, G. & Joubert, D. From ultrasoft pseudopotentials to the projector augmented-wave method. *Phys. Rev. B.* **59,** 1758-1775 (1999).

35. Grimme, S., Antony, J., Ehrlich, S. & Krieg, H. A consistent and accurate ab initio parametrization of density functional dispersion correction (DFT-D) for the 94 elements H-Pu. *J. Chem. Phys*. **132**, 154104 (2010).
36. Malig, T. C., Yu, D. & Hein, J. E. A revised mechanism for the kinugasa reaction. *J. Am. Chem. Soc.* **140,** 9167-9173 (2018).
37. Fertig, R., Irrgang, T., Freitag, F., Zander, J. & Kempe, R. Manganese-catalyzed and base-switchable synthesis of amines or imines via borrowing hydrogen or dehydrogenative condensation. *ACS Catal.* **8,** 8525-8530 (2018).
38. Bains, A. K., Kundu, A., Yadav, S. & Adhikari, D. Borrowing hydrogen-mediated N-alkylation reactions by a well-defined homogeneous nickel catalyst. *ACS Catal.* **9,** 9051-9059 (2019).
39. Wong, C. M., Peterson, M. B., Pernik, I., McBurney, R. T. & Messerle, B. A. Highly efficient Rh(I) homo- and heterogeneous catalysts for C–N couplings via hydrogen borrowing. *Inorg. Chem.* **56,** 14682-14687 (2017).
40. Xu, Z., Yu, X., Sang, X. & Wang, D. BINAP-copper supported by hydrotalcite as an efficient catalyst for the borrowing hydrogen reaction and dehydrogenation cyclization under water or solvent-free conditions. *Green Chem.* **20,** 2571-2577 (2018).
41. Babu, R., Sukanya Padhy, S., Kumar, R. & Balaraman, E. Catalytic amination of alcohols using diazo compounds under manganese catalysis through hydrogenative N-alkylation reaction. *Chem. Eur. J.* **29,** e202302007 (2023).
42. Su, W. et al. Catalytic N-alkylation of amines with aldehydes by a molecular Mo oxide catalyst. *Eur. J. Inorg. Chem.* **26,** e202200751 (2023).
43. Chakraborty, S. et al. Zn(II)-catalyzed selective N-alkylation of amines with alcohols using redox noninnocent azo-aromatic ligand as electron and hydrogen reservoir. *J. Org. Chem.* **88,** 771-787 (2023).
44. Zhu, G., Duan, Z.-C., Zhu, H., Ye, D. & Wang, D. Selective C-C bonds formation, N-alkylation and benzo [d] imidazoles synthesis by a recyclable zinc composite. *Chin. Chem. Lett.* **33,** 266-270 (2022).
45. Fu, M.-X., Lin, J.-H. & Xiao, J.-C. Desulfurization of thiols for nucleophilic substitution. *Org. Lett.* **26,** 6065-6069 (2024).
46. Song, D. et al. A recyclable ciprofloxacin polymer ligand for copper-catalyzed coupling of (hetero) aryl halide aminations. *New J. Chem.* **46,** 19100-19103 (2022).
47. Li, S., Rajeshkumar, T., Liu, J., Maron, L. & Zhou, X. La-catalyzed decarbonylation of formamides and its applications. *Org. Lett.* **25,** 163-168 (2023).
48. Boumekla, Y. et al. Calcium-catalysed synthesis of amines through imine hydrosilylation: an experimental and theoretical study. *Org. Biomol. Chem.* **21,** 1038-1045 (2023).
49. Yang, W., Wei, L., Yi, F. & Cai, M. Magnetic nanoparticle-supported phosphine gold (i) complex: a highly efficient and recyclable catalyst for the direct reductive amination of aldehydes and ketones. *Catal. Sci. Technol.* **6,** 4554-4564 (2016).
50. Qian, C.-W., Li, X., Xiang, W. & Gu, M.-Q. Diazonium salt as a versatile, efficient and mild catalyst for reductive aminations of carbonyls and syntheses of bis (indolyl) methanes. *Tetrahedron* **151,** 133789 (2024).
51. Bastick, K. A. C. & Watson, A. J. B. Pd-catalyzed homologation of arylboronic acids as a platform for the diversity-oriented synthesis of benzylic C–X bonds. *Synlett.* **34,** 2097-2102

(2023).
52. Vidal, F., McQuade, J., Lalancette, R. & Jäkle, F. ROMP-boranes as moisture-tolerant and recyclable lewis acid organocatalysts. *J. Am. Chem. Soc.* **142,** 14427-14431 (2020).
53. Chung, H. & Chung, Y. K. Cobalt–rhodium heterobimetallic nanoparticle-catalyzed N-alkylation of amines with alcohols to secondary and tertiary amines. *J. Org. Chem.* **83,** 8533-8542 (2018).
54. Wei, D., Bruneau-Voisine, A., Valyaev, D. A., Lugan, N. & Sortais, J.-B. Manganese catalyzed reductive amination of aldehydes using hydrogen as a reductant. *Chem. Commun.* **54,** 4302-4305 (2018).
55. Babu, R., Sukanya Padhy, S., Kumar, R. & Balaraman, E. Catalytic amination of alcohols using diazo compounds under manganese catalysis through hydrogenative N-alkylation reaction. *Chem. Eur. J.* **29,** e202302007 (2023).
56. Bauri, S., Donthireddy, S. N. R., Illam, P. M. & Rit, A. Effect of ancillary ligand in cyclometalated Ru(II)–NHC-catalyzed transfer hydrogenation of unsaturated compounds. *Inorg. Chem.* **57,** 14582-14593 (2018).
57. Nandy, A. & Sekar, G. Dibenziodolium salts as halogen bond donor catalysts for the reduction of quinolines, one-pot reductive amination, and addition reaction with indoles. *Eur. J. Org. Chem.* **2022,** e202200982 (2022).
58. Konishi, H., Tanaka, H. & Manabe, K. Pd-catalyzed selective synthesis of cyclic sulfonamides and sulfinamides using $K_2S_2O_5$ as a sulfur dioxide surrogate. *Org. Lett.* **19,** 1578-1581 (2017).
59. Sakai, N. et al. Indium-catalyzed reduction of secondary amides with a hydrosiloxane leading to secondary amines. *Tetrahedron Lett.* **56,** 6448-6451 (2015).
60. Kallmeier, F., Fertig, R., Irrgang, T. & Kempe, R. Chromium-catalyzed alkylation of amines by alcohols. *Angew. Chem. Int. Ed.* **59,** 11789-11793 (2020).
61. Huang, M. et al. Ruthenium(ii) complexes with N-heterocyclic carbene–phosphine ligands for the N-alkylation of amines with alcohols. *Org. Biomol. Chem.* **19,** 3451-3461 (2021).
62. Guin, A. K., Pal, S., Chakraborty, S., Chakraborty, S. & Paul, N. D. N-alkylation of amines by C1–C10 aliphatic alcohols using a well-defined Ru(II)-catalyst. a metal–ligand cooperative approach. *J. Org. Chem.* **88,** 5944-5961 (2023).
63. Zhang, C., Zhan, Z., Lei, M. & Hu, L. Ullmann-type C–N coupling reaction catalyzed by CuI/metformin. *Tetrahedron* **70,** 8817-8821 (2014).
64. Kolesnikov, P. N., Yagafarov, N. Z., Usanov, D. L., Maleev, V. I. & Chusov, D. Ruthenium-catalyzed reductive amination without an external hydrogen source. *Org. Lett.* **17,** 173-175 (2015).
65. Kumar, R. et al. Catalytic hydroboration and reductive amination of carbonyl compounds by HBpin using a zinc promoter. *Chem. Eur. J.* **17,** e202200013 (2022).
66. Behera, S. et al. Application of bertagnini's salts in a mechanochemical approach toward aza-heterocycles and reductive aminations via imine formation. *Adv. Synth. Catal.* **366,** 2035-2043 (2024).
67. Sadekov, I. D., Maksimenko, A. A., Maslakov, A. G. & Minkin, V. I. Chemical consequences of intramolecular Te ← N coordination in tellurium-containing aromatic azomethine derivatives. *J. Organomet. Chem.* **391,** 179-188 (1990).
68. Li, H.-M., Xu, C., Wang, Z.-Q. & Fu, W.-J. A half sandwich ruthenium(II) complex with N-

4,6-dimethyl-2-pyrimidinylimidazole: synthesis, crystal structure and application in N-alkylation of amines with alcohols. *Bull. Korean Chem. Soc.* **36,** 2557-2560 (2015).

69. Chaudhary, P. et al. An efficient metal-free method for the denitrosation of aryl N-nitrosamines at room temperature. *Adv. Synth. Catal.* **360,** 556-561 (2018).
70. Lei, Q. et al. Fast reductive amination by transfer hydrogenation "on water". *Chem. Eur. J.* **19,** 4021-4029 (2013).
71. Yu, X., Liu, C., Jiang, L. & Xu, Q. Manganese dioxide catalyzed N-alkylation of sulfonamides and amines with alcohols under air. *Org. Lett.* **13,** 6184-6187 (2011).
72. Li, X. et al. Efficient and practical catalyst-free-like dehydrative N-alkylation of amines and sulfinamides with alcohols initiated by aerobic oxidation of alcohols under air. *Tetrahedron* **72,** 264-272 (2016).
73. Wei, D. et al. N-alkylation of amines with alcohols catalyzed by manganese(II) chloride or bromopentacarbonylmanganese(I). *J. Org. Chem.* **86,** 2254-2263 (2021).
74. Budén, M. E., Dorn, V. B., Gamba, M., Pierini, A. B. & Rossi, R. A. Electron-transfer-mediated synthesis of phenanthridines by intramolecular arylation of anions from N-(ortho-halobenzyl)arylamines: regiochemical and mechanistic analysis. *J. Org. Chem.* **75,** 2206-2218 (2010).
75. Mondal, S., Pal, S., Khanra, S., Chakraborty, S. & Paul, N. D. Co-catalyzed metal-ligand cooperative approach for N-alkylation of amines and synthesis of quinolines via dehydrogenative alcohol functionalization. *Eur. J. Inorg. Chem.* **26,** e202300263 (2023).
76. Li, J. et al. Preparation of a novel cadmium-containing coordination polymer and catalytic application in the synthesis of N-alkylated aminoquinoline derivatives via the borrowing hydrogen approach. *Dalton Trans.* **53,** 5064-5072 (2024).
77. Wang, C. et al. Insight into O_2-promoted base-catalyzed N-alkylation of amines with alcohols. *Eur. J. Org. Chem.* **2015,** 2972-2977 (2015).
78. Borthakur, I., Maji, M., Joshi, A. & Kundu, S. Reductive alkylation of azides and nitroarenes with alcohols: a selective route to mono- and dialkylated amines. *J. Org. Chem.* **87,** 628-643 (2022).
79. Nguyen, Q. P. B. & Kim, T. H. S-benzyl isothiouronium chloride as a recoverable organocatalyst for the direct reductive amination of aldehydes. *Tetrahedron Lett.* **52,** 5004-5007 (2011).
80. Samec, J. S. M., Mony, L. & Bäckvall, J.-E. Efficient ruthenium catalyzed transfer hydrogenation of functionalized imines by isopropanol under controlled microwave heating. *Can. J. Chem.* **83,** 909-916 (2005).
81. Huang, M. et al. Room temperature N-heterocyclic carbene manganese catalyzed selective N-alkylation of anilines with alcohols. *Chem. Commun.* **55,** 6213-6216 (2019).
82. Wu, D., Bu, Q., Guo, C., Dai, B. & Liu, N. Cooperative catalysis of molybdenum with organocatalysts for distribution of products between amines and imines. *Mol. Catal.* **503,** 111415 (2021).
